# Plant-to-plant communication triggered by systemin primes anti-herbivore resistance in tomato

**DOI:** 10.1038/s41598-017-15481-8

**Published:** 2017-11-14

**Authors:** Mariangela Coppola, Pasquale Cascone, Valentina Madonna, Ilaria Di Lelio, Francesco Esposito, Concetta Avitabile, Alessandra Romanelli, Emilio Guerrieri, Alessia Vitiello, Francesco Pennacchio, Rosa Rao, Giandomenico Corrado

**Affiliations:** 1 Dipartimento di Agraria, Università degli Studi di Napoli Federico II, Via Università 100, 80055 Portici, NA Italy; 2 Istituto per la Protezione Sostenibile delle Piante, CNR, Via Università 133, Portici, NA Italy; 3 Istituto di Biostrutture e Bioimmagini (CNR), via Mezzocannone 16, 80134 Napoli, Italy; 4 Dipartimento di Farmacia, Università degli Studi di Napoli Federico II, Via Domenico Montesano, 49, 80131 Napoli, NA Italy

## Abstract

Plants actively respond to herbivory by inducing various defense mechanisms in both damaged (locally) and non-damaged tissues (systemically). In addition, it is currently widely accepted that plant-to-plant communication allows specific neighbors to be warned of likely incoming stress (defense priming). Systemin is a plant peptide hormone promoting the systemic response to herbivory in tomato. This 18-aa peptide is also able to induce the release of bioactive Volatile Organic Compounds, thus also promoting the interaction between the tomato and the third trophic level (e.g. predators and parasitoids of insect pests). In this work, using a combination of gene expression (RNA-Seq and qRT-PCR), behavioral and chemical approaches, we demonstrate that systemin triggers metabolic changes of the plant that are capable of inducing a primed state in neighboring unchallenged plants. At the molecular level, the primed state is mainly associated with an elevated transcription of pattern -recognition receptors, signaling enzymes and transcription factors. Compared to naïve plants, systemin-primed plants were significantly more resistant to herbivorous pests, more attractive to parasitoids and showed an increased response to wounding. Small peptides are nowadays considered fundamental signaling molecules in many plant processes and this work extends the range of downstream effects of this class of molecules to intraspecific plant-to-plant communication.

## Introduction

Plants have developed multiple defense strategies in a continuous co-evolution with pests. Plant defense mechanisms can be expressed constitutively or induced upon injury^1^. On the basis of the mode of action, defense traits are also distinguished in direct and indirect^1,2^. The former includes, for instance, physical barriers and metabolites that directly interfere with pests, while the latter involves any plant trait that attracts natural enemies of pests, such as predators and parasitoids^1,2^.

Systemin is an 18 amino acid (aa) hormone that was firstly identified as a limited range mobile inducer in the wound response in tomato^3^. This peptide is released from a 200 aa precursor, called prosystemin, which is encoded by a single-copy inducible gene^4^. The overexpression of prosystemin cDNA in tomato generates a systemic signal that activates defense genes^5^. It has been proposed that systemin and jasmonic acid (JA) interact through an amplification loop to propagate a long-distance wound signal^6,7^. Although systemin was isolated by exploiting its ability to strongly induce protease inhibitors (PIs)^6^, further studies indicated that this peptide stimulates a wider transcriptome reprogramming, affecting the expression of genes involved in different hormone-regulated pathways^8^. The constitutive accumulation of prosystemin increases the resistance of plants not only against lepidopteran larvae but also against some fungi and aphids^5,8,9^. Moreover, prosystemin overexpressing plants are more attractive to natural enemies of pests^10,11^ because of the release of various bioactive Volatile Organic Compounds (VOCs)^10^, known to be typically used by predators and parasitoids of phytophagous insects to locate their prey^12^.

The constitutive increase of indirect defenses in absence of pests could be of little use from an applied point of view, because the attraction of natural enemies not associated with the presence of potential hosts results in dispersion of natural enemies due to lack of motivation^13^. Therefore, it is also interesting to assess whether the induction of systemin- can prime the adjacent plants and make them able to mount a more effective defense reaction. Manipulating plant response to biotic stressors by tuning the systemin molecular network may open the way to novel sustainable strategies for plant protection.

Priming is a physiological process through which the functions and activities of an unstressed plant are dedicated to support a more rapid and robust response to a probable future challenge^14,15^. Priming may be trigged by different cues such as pathogens, insect pests, molecules of microbial origin, synthetic substances and abiotic stress^16^. Molecular mechanisms linked to priming are diverse and involve chromatin modification for faster activation of defense genes and epigenetic memory^17^. Activation of proteins involved in signal transduction, receptor accumulation and increased levels of some defense compounds also represent common events in priming^17,18^. Priming should not be limited to the expression of defense-related genes, as it may involve other plant responses^14^. Many studies on priming anti-herbivory defense have primarily focused on Herbivore Induced Plant Volatile (HIPVs), since they are also associated to intra-plant, co-specific and interspecific communication^15^. However, information on plant-endogenous signals that can trigger plant-to-plant communication is in comparison more limited. Moreover, differences between priming signal pathways and direct induced defences as well as their mechanistic similarities have not been fully elucidated.

Since systemin regulates direct and indirect defenses in tomato^5,10^, the aim of this work was to study if systemin can trigger plant-to-plant communication. Specifically, we wanted to address whether systemin overexpression or exogenous application is able to prime a defense response in unchallenged neighboring plants, and whether this alerted state improves resistance against herbivorous pests and plant response to wounding. Moreover, we aimed to provide insights into the molecular aspects that characterize the primed state in tomato.

## Results

### Systemin affects the transcriptome of receiver plants

**Figure 1:**
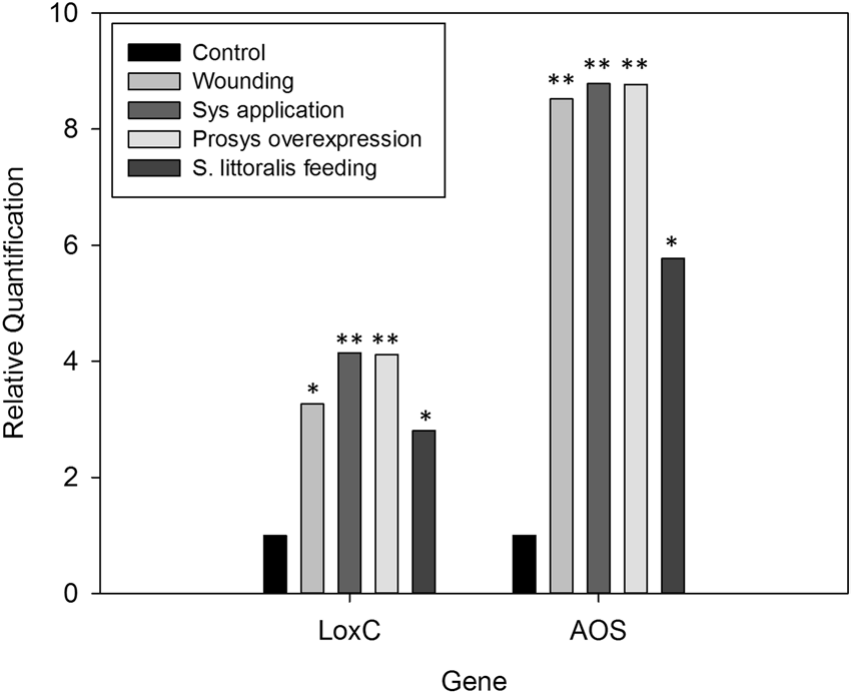
Analysis of the systemin treatment on tomato plants. Relative gene expression by real-time PCR of genes involved in the wounding response of tomato, lipooxygenase C (LoxC) and allene oxide synthase (AOS) following elicitation with the systemin peptide. Quantities are shown relative to the calibrator control condition, set as 1 (untreated plants). Wounding: tomato leaves six hour following mechanical wounding; Sys application: leaves after application (24 h) of the systemin peptide (S1, Table 1); Prosys overexpressing: leaves from transgenic plants constitutively expressing the prosystemin cDNA (S2, Table 1); S. littoralis feeding: leaves following one hour of *Spodoptera littoralis* feeding (S3, Table 1). For each experimental condition asterisks indicate statistically significant differences compared to control condition (*p < 0.05; **p < 0.01; t-test).

To study the effect of the systemin peptide on plant-to-plant communication, we elicited tomato plants by spotting a systemin solution on the abaxial face of fully expanded leaves. Before plant treatment, the stability of the peptide was analyzed for up to 48 hours. The HPLC profiles and mass spectra indicated that the peptide is stable in the tested conditions (Supplementary Figure 1). To validate the molecular effects of the systemin application on leaf surface, we analyzed the level of expression of two genes involved in tomato wound response, the *Lipoxygenase C* (*LoxC*) and the *Allene Oxidase Synthase* (*AOS*). The foliar application of the systemin peptide induced the expression of the two selected genes, similarly to wounding, larval feeding and the overexpression of the prosystemin cDNA as shown in Fig. 1.

Subsequently, the effect of the exposition to systemin-elicited (S1) or mock-treated plants (S4) on receiving plants (R-S1 and R-S4, respectively) was analyzed by RNA-Seq (Table 1 summarizes the different experimental set-ups employed in this study and their code). The six datasets (three biological replicates per two conditions) were mapped against the tomato reference genome (SL2.50). The mean input and its standard deviation was 64,398,103.67 and 62,598,242.67 reads per sample, before and after quality control and trimming, respectively, with an average trimming of 2.78%. The mean mapping rate to the tomato genome was 93.15 ± 0.79%, which indicated that a small portion of reads were not counted in the RNA-Seq reads mapping process (Supplementary Table 1).

**Table 1:** Plant material.

Source description (code)	Receiver description (code)	Code of receiver plants following wounding
Systemin treated plants (S1)	Tomato plants exposed to S1 (R-S1)	WR-S1
Prosystemin over-expressing plants (S2)	Tomato plants exposed to S2 (R-S2)	WR-S2
Plants chewed by *S*. *littoralis* larvae (S3)	Tomato plants exposed to S3 (R-S3)	WR-S3
Untreated tomato plants (S4)	Tomato plants exposed to S4 (R-S4)	WR-S4

**Figure 2:**
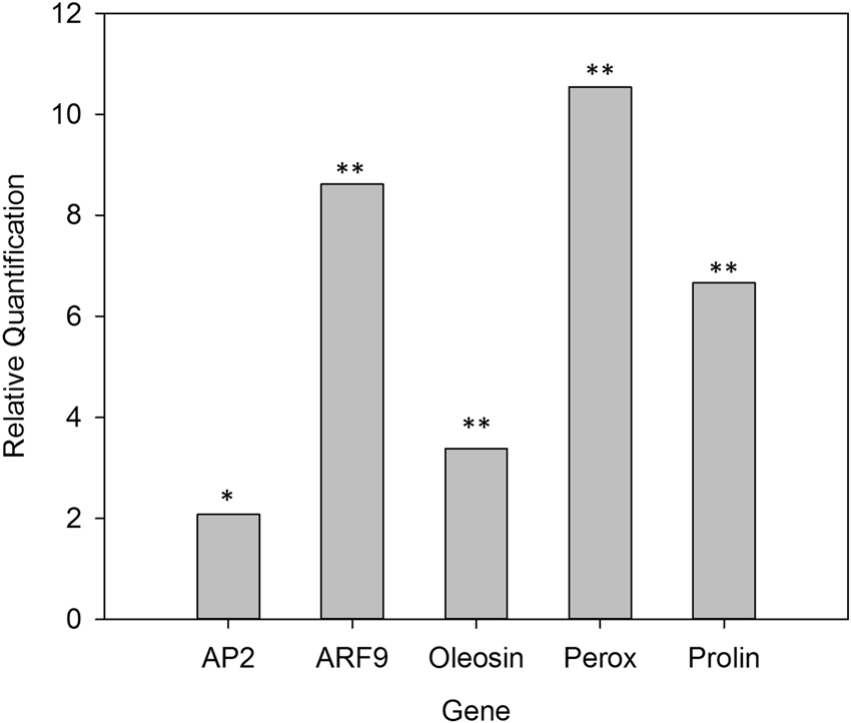
A comparison between wounding response and the systemin dependent primed state in tomato. The gene expression analysis of five wound-responsive DEGs (Supplementary Figure 2) was carried out by comparing tomato leaves six hours following wounding with leaves of the R-S1 plants. Quantities are shown relative to the R-S1 condition (Table 1). AP2: Ap2-like ethylene-responsive transcription factor; ARF 9: Auxin response factor 9; Knotted: Homeobox protein knotted-1; Oleosin: Oleosin 1; Perox: Peroxidase 3; PD: Proline dehydrogenase mitochondrial. Asterisks indicate statistically significant differences compared to R-S1 condition (*p < 0.05; **p < 0.01; t-test).

Differential analysis of RNA-Seq data between receivers of systemin-elicited or untreated plants identified 537 up-regulated and 581 down-regulated transcripts (Supplementary Tables 2 and 3). The real-time RT-PCR validation of nine differentially expressed genes (DEGs) is reported in the Supplementary Table 4. The expression of six DEGs following wounding was performed to verify their responsiveness to this stimulus. The real-time PCR analysis indicated that five out six selected DEGs are induced by wounding (Supplementary Figure 2), implying that wound response does not fully overlap with the primed state. Moreover, priming of these five wound-inducible DEGs by wounding or systemin treatment was found to be quantitatively different. The comparison between the level of expression in mechanically wounded leaves and in leaves of the R-S1 plants indicated that all the selected genes were expressed at significantly higher level following wounding (Fig. 2).

**Figure 3:**
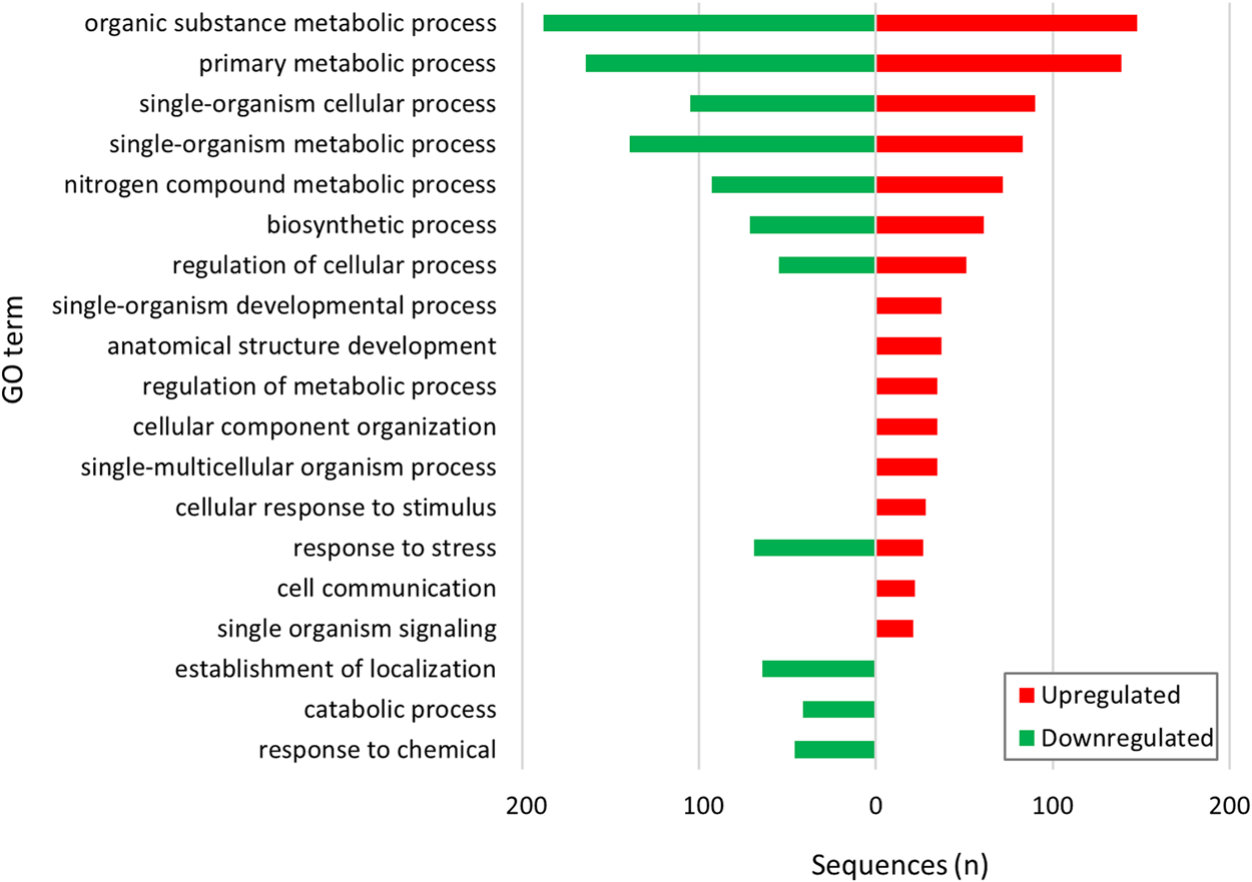
GOs distribution of differentially expressed genes in receiver plants exposed to systemin-treated plants. Gene Ontology (GO) terms associated to up-regulated (red bars) and down-regulated (green bars) genes based on the “Biological Process” ontological domain (sequence cut-off: 5%).

DEGs’ functional annotation and subsequent data mining was based predominantly on the Gene Ontology (GO) vocabulary (Supplementary Tables 2 and 3). The “Biological Process” level 3 ontology terms associated to DEGs indicated that exposition to systemin-treated plants induced an alteration of different processes (Fig. 3). “Cell communication” and “single organism signalling” (resp. “catabolic process” and “response to chemical”) were among the GO-terms present mainly in the overexpressed sequences (resp. under-expressed). DEGs that were annotated into GO-categories related to stress response and signaling included those coding for kinases (e.g. serine threonine-protein kinase, NSP-interacting kinase 2, Pto-like serine/threonine kinase protein resistance protein, ATP binding/serine-threonine kinase, serine threonine-protein kinase wag1) and receptor-like kinases (RLKs) (e.g. Receptor-like protein kinase 2, leucine-rich repeat receptor-like protein kinase, Receptor-like serine threonine-protein kinase ale2, LRR receptor-like serine/threonine-protein kinase 2 C, LRR receptor-like serine threonine-protein kinase fei 2, leucine-rich repeat receptor- serine threonine-protein kinase bam3). Among the overexpressed genes related to epigenetic control of gene expression, two Histone-lysine N-methyltransferases were upregulated, along with two genes coding for the Histones H4 and H3, respectively. Exposition to S1 plants altered in receiver plants some herbivory-related responses, such as genes that can directly affect phytophagous pests (e.g. Peroxidase, Proteinase inhibitor II, Metallocarboxy proteinase inhibitor and Polyphenol oxidase genes) and genes connected to VOCs biosynthesis (geraniol-8-hydroxylase). Other functional categories of DEGs connected to plant reaction to stress were “response to external and endogenous stimuli” and “response to abiotic and biotic stimuli”. Genes coding for members of different families of transcription factors (TF) (e.g. ERF, bZIP and MYB) such as those typically regulating plant responses to stress (e.g. WRKY and NAC-domain proteins) were included in these categories^19^. Furthermore, 16 salicylic acid (SA) responsive genes were down-regulated, such as those coding for different classes of pathogenesis-related proteins (osmotin, β-glucosidase 13, pathogenesis-related protein 4, pathogenesis-related protein STH2, subtilisin and chitinases). Among genes related to phytohormone pathways, ten associated to ethylene were down-regulated (e.g. 1-aminocyclopropane-1-carboxylate oxidase 1 and 4 (ACO) homologs and ethylene-responsive factors).

The identification of “Molecular Functions” categories that show statistically significant differences between the primed and control state was carried out by a Gene Set Enrichment Analysis (GSEA), for upregulated and downregulated DEGs (Supplementary Tables 5 and 6, respectively). The vast majority and the more statistically significant GO-enriched terms for the overexpressed genes were related to molecular transducer activities, such as signaling, kinase and receptor activities. On the other hand, the most enriched GO-term for the down-regulated sequences were related to transporter activities relative to different types of organic and inorganic molecules.

For biological interpretation of higher-level systemic functions, we mapped the enzymatic activities of the annotated DEGs to the KEGG reference pathway maps. The KEGG analysis indicated that several pathways were differentially affected in R-S1 plants (Supplementary Table 7). The higher number of DEGs belonged to the “Starch and sucrose metabolism” reference pathway, followed by “Biosynthesis of antibiotics”. The latter includes a number of reactions that in plants are also associated to terpenoid biosynthesis, the shikimate pathway and other secondary metabolites. Other reference pathways with a high number of DEGs were the “Glutathione metabolism” and the “Phenylpropanoid biosynthesis”. Glutathione acts as an anti-oxidant in response to Reactive Oxygen Species and it is expected that plants, as part of their response to stress, produce glutathione^20^. Phenylpropanoids are a large class of compounds and are involved in structural support as well as plant defense^21^. KEGG analysis also indicated that four down-regulated genes are related to the fatty acid degradation (3-hydroxyacyl-dehydrogenase, isomerase, dehydrogenase (NAD+), Long-chain-fatty-acid coA ligase) and phenylalanine metabolism.

### Priming caused by prosystemin overexpressing transgenic plants and insect-damaged plants

**Figure 4:**
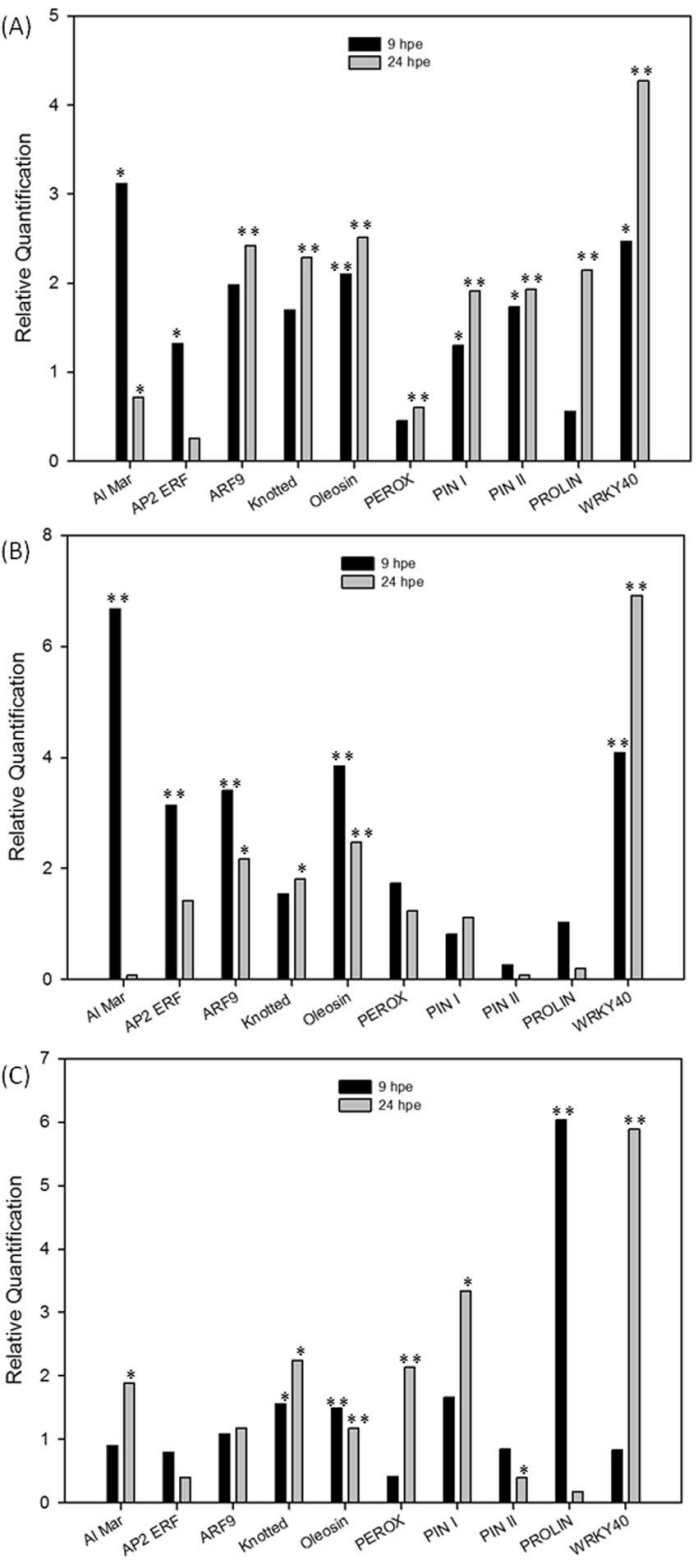
Relative quantification of defense transcripts upon exposition to different source plants. Expression analysis of defense genes by Real Time RT-PCR in receiver plants R-S1 (**A**), R-S2 (**B**) and RS-3 (**C**) 9 and 24 hours following exposition (hpe). Relative quantities (RQ) are calibrated on R-S4 plants and indicated on a linear scale. Asterisks indicate statistically significant differences compared to control condition at the same time point (*p < 0.05; **p < 0.01; t-test).

To evaluate the biological impact of the systemin elicitation, we also analyzed receivers exposed to transgenic source plants overexpressing the systemin precursor (S2). In addition, we analyzed the tomato response to the exposition of source plants in which the prosystemin expression, along with other responses, was activated by phytophagous larvae (S3) (Supplementary Figure 3). The gene expression analysis by real-time PCR was performed on ten DEGs associated to stress phytohormones or defense response. Leaves of receiver plants (R-S1, R-S2 and R-S3) were harvested 9 and 24 h after exposure. The three types of source plants were able to increase the expression of the selected defense gene in receivers, indicating that not only systemin exogenous treatment but also the transgenic overexpression of its precursor influences plant-to-plant communication (Fig. 4). Although quantitative differences were present in the expression level of the different types of receivers, the strongest similarity was observed between R-S1 and R-S2 plants. Overall, the overexpression in R-S1 plants was lower compared to plants exposed to prosystemin overexpressing transgenics. The weakest activation was noticed in receivers exposed to plants injured by one hour of larval feeding. In R-S1, R-S2 and R-S3 plants, WRKY40 was the gene with the highest overexpression at the latest time-point.

### Systemin increases resistance to phytophagous larvae in receiver plants

To test whether systemin can improve insect resistance in receivers, we assessed the performance of R-S1, R-S2 and R-S3 plants against *Spodoptera littoralis*, using a no-choice feeding bioassay. Experimental larvae were singly isolated in a multi-well plastic tray, covered by transparent lids for continuous inspection, and offered with leaf disks. This allowed an easy monitoring of growth and survival of the experimental larvae, under standardized and controlled conditions. A set of control naïve receivers (R-S4) was used as control in each experiment.

**Figure 5:**
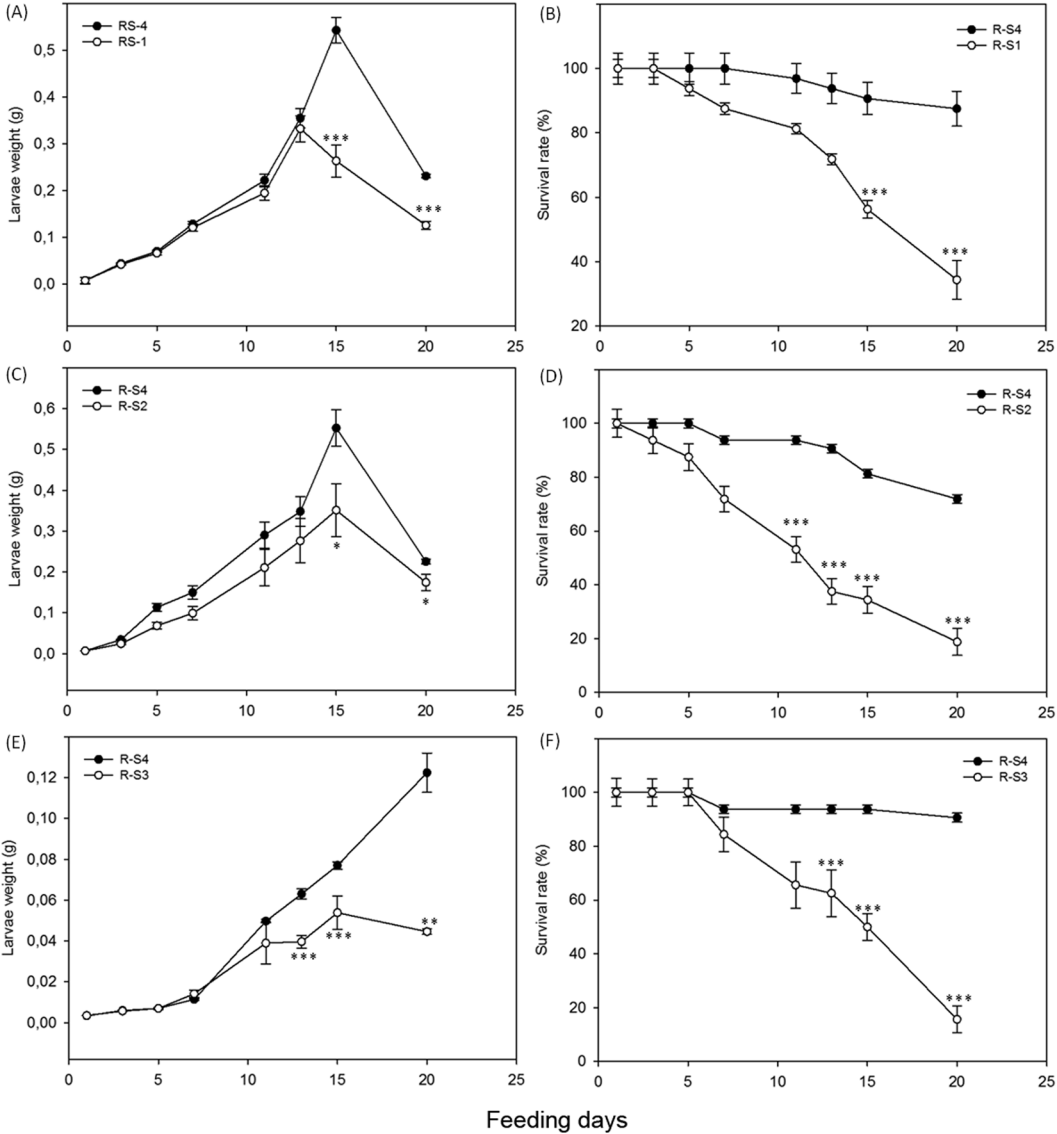
Increased resistance against larvae of receiver plants. Weight (mean ± s.e.m.) and survival rate of *Spodoptera littoralis* larvae fed on R-S1, R-S2, R-S3 (white dots) or R-S4 leaves (black dots). Asterisks indicate statistically significant differences compared to the control condition (R-S4) at each time point (*p < 0.05, **p < 0.01, ***p < 0.001; Logrank test).

The weight (Fig. 5A) and survival rate (Fig. 5B) of *S*. *littoralis* feeding on R-S1 plants were significantly reduced compared to the control (R-S4). Similar results were obtained for larvae fed on plants exposed to prosystemin overexpressing plants. Starting from the fifth day, the weight of larvae fed on R-S2 leaves was significantly lower than the control (Fig. 5C). The survival rate was also reduced throughout the bioassay (Fig. 5D). After 20 days of feeding, the survival rate was 19% for larvae fed on R-S2 leaves and 72% for larvae fed on controls (R-S4). Weight gain was reduced for *S*. *littoralis* fed on R-S3 leaves (Fig. 5F). The survival rate reached 50% on R-S3 leaves in comparison to 94% for controls at 15 days of feeding and, at the end of the bioassay, no live larva was found for the R-S3 leaves, compared to an 86% survival rate for the control leaves (Fig. 5E).

### Systemin increases indirect defense in receiver plants

**Figure 6:**
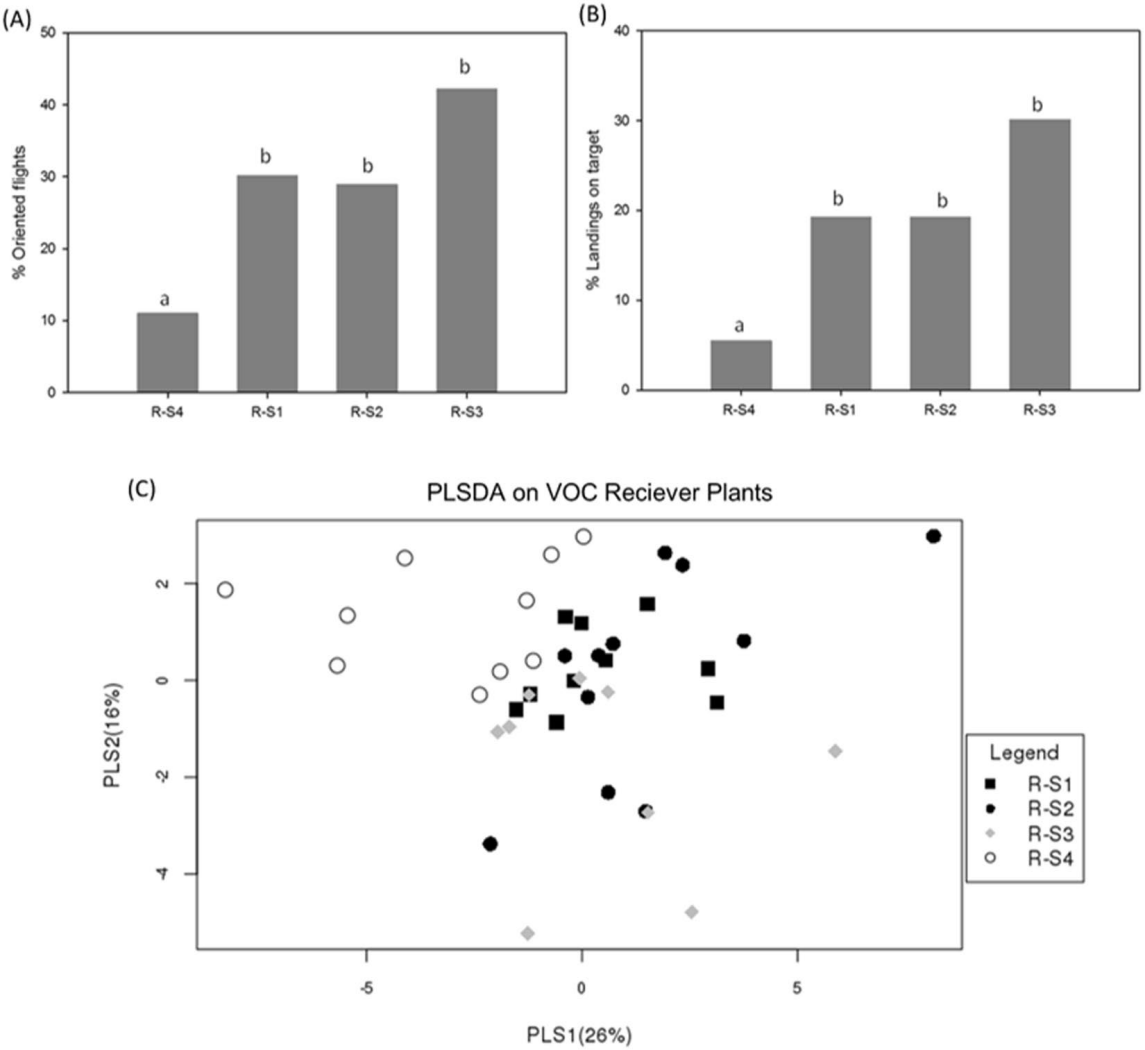
Increased indirect defense of receiver plants. Flight response (**A**) percentages of oriented flights; (**B**) landing on target) of naïve *Aphidius ervi* females to the different receiver plants (R-S1, R-S2, R-S3 or R-S4). Different letters indicate statistically different groups (*p* < 0.05; one-way ANOVA): (**C**) PSLD analysis on the Volatile Organic Compounds emitted by the different receiver plants.

We also evaluated the possible increase of indirect defenses elicited by systemin. To this aim, we analyzed the behavioral response of the aphid parasitoid *Aphidius ervi* to the different receivers using a wind-tunnel assay. R-S1, R-S2 and R-S3 plants were significantly more attractive to the parasitoid than R-S4 plants in terms of both oriented flights (Fig. 6A) and landings on target (Fig. 6B). To investigate whether the observed increase in attractiveness relates to variation in the release of volatile compounds, we compared the VOCs emitted by the different receiver plants. The different source plants were able to induce qualitative and quantitative changes in the volatile blends of receivers (Table 2). Specifically, β-ocimene was detected only in R-S1, R-S2 and R-S3 plants. On the other hand, 2-ethylhexanal and benzene, 1-ethyl-3-methyl- were emitted only by naïve plants. PLSDA analysis on VOCs illustrated the overall differences between receiver of naïve or of source plants (Fig. 6C). Collectively, these data indicate that exposition to systemin-treated sources increases plant’s indirect defense and selectively enhances the release of organic compounds with a known role in tri-trophic interactions.

**Table 2:** Headspace Volatile Organic Compounds collected from receiver plants.

Compound	Mean values (pg g^−1^ fresh weight) ± s.e.			
	R-S1	R-S2	R-S3	R-S4
2,4-Dimethyl-1-heptene	21.1185 ± 4.4608	12.1636 ± 2.2963	13.8323 ± 2.597	13.3111 ± 3.3908
Ethylbenzene	2.3097 ± 0.3994	2.3475 ± 0.4796	2.3484 ± 0.3575	2.7257 ± 0.6377
p-Xylene	8.6292 ± 1.4987	6.998 ± 1.2153	6.624 ± 1.2566	8.2506 ± 2.1974
Amyl acetate	0.489 ± 0.0766	0.5146 ± 0.14	0.4247 ± 0.0796	0.4807 ± 0.1049
2-Ethylhexanal^†^	—	—	—	0.3531
4-Methylnonane	1.9951 ± 0.6047	1.0561 ± 0.1576	0.727 ± 0.2447	1.1947 ± 0.4201
Benzene, 1-ethyl-3-methyl-^†^	—	—	—	0.0557
benzaldehyde	1.999 ± 0.75	2.0219 ± 0.6277	2.4066 ± 0.5362	3.8278 ± 1.5466
1-heptanol	1.0527 ± 0.4114	0.9293 ± 0.3244	1.1244 ± 0.3817	1.3657 ± 0.4278
6-Methyl-5-hepten-2-one ^†^	0.0279	—	—	—
Benzene, 1,3,5-trimethyl-^*^	1.6267 ± 0.4142	1.006 ± 0.2542	0.5463 ± 0.2498	0.8002 ± 0.2682
1,4-dichlorobenzene	7.667 ± 1.5971	6.0373 ± 1.2119	6.136 ± 1.177	7.622 ± 2.2909
β-Ocimene*	0.2265 ± 0.1035	0.1609 ± 0.0959	0.0489 ± 0.0443	—
2-ethyl-1-hexanol	8.5359 ± 1.6822	7.1676 ± 1.4032	7.1111 ± 1.4065	9.9573 ± 3.0689
acetophenone*	1.438 ± 0.4395	1.2966 ± 0.1957	0.9845 ± 0.1998	1.3795 ± 0.387
p-tolualdehyde	3.7601 ± 0.9613	2.3816 ± 0.3119	1.7538 ± 0.4484	2.706 ± 0.7754
Methyl benzoate	0.3758 ± 0.1115	0.1327 ± 0.0602	0.1875 ± 0.0656	0.3088 ± 0.1149
nonanal	1.0921 ± 0.2091	1.267 ± 0.2963	1.2065 ± 0.2345	1.4374 ± 0.491
camphor	0.7695 ± 0.1488	0.6473 ± 0.1606	0.5334 ± 0.0855	0.6584 ± 0.2237
naphthalene	37.4862 ± 6.8839	27.2688 ± 4.7471	26.2957 ± 4.6487	31.9383 ± 9.1619
1-dodecene	4.1405 ± 0.7282	3.1478 ± 0.6093	3.1018 ± 0.6402	3.6768 ± 1.2184
decanal	1.0878 ± 0.2345	0.7909 ± 0.2322	0.7063 ± 0.1913	1.0781 ± 0.3643
Benzaldehyde, 2,4-dimethyl-	0.7173 ± 0.1489	0.6024 ± 0.1355	0.4959 ± 0.094	0.6487 ± 0.1646
4-vinylphenol	0.0297 ± 0.028	0.1929 ± 0.0677	0.0925 ± 0.0925	0.0226 ± 0.0226
Ethanol, 2-phenoxy-	1.0697 ± 0.3511	0.6518 ± 0.2817	0.3234 ± 0.1734	1.2439 ± 0.4482
benzothiazole	1.3984 ± 0.2395	1.517 ± 0.3732	0.7183 ± 0.1567	1.2738 ± 0.3447
α-pinene	0.4563 ± 0.0895	0.4042 ± 0.0983	0.4061 ± 0.0754	0.4701 ± 0.1301
limonene	0.4216 ± 0.0817	0.3408 ± 0.0709	0.3464 ± 0.065	0.3879 ± 0.1136
linalool	0.785 ± 0.1143	0.651 ± 0.1388	0.6061 ± 0.1115	0.7579 ± 0.2429

Asterisks indicate significant differences among treatments (Kruskal-Wallis One Way Analysis of Variance on Ranks); a Latin cross denotes that the compound was exclusively detected in one type of receiver. See Table 1 for the plant code description.

### Expression analysis of the wound response of systemin-primed plants

To examine whether the observed resistance to larvae is based on an improved response to incoming stress in primed plants, we quantified the expression of 11 genes after elicitation of plant defense by mechanical wounding. Immediately after 24 hours of exposition, leaves of the different receiver plants (R-S1, R-S2, R-S3 and R-S4) were mechanically wounded. The damaged plants were named WR-S1, WR-S2, WR-S3 and WR-S4. The gene expression analysis was carried out by harvesting leaves at 3, 6 and 24 hours after damage. To evaluate differences in response to wounding, expression data were calibrated on wounded naïve plants (WR-S4).

**Figure 7:**
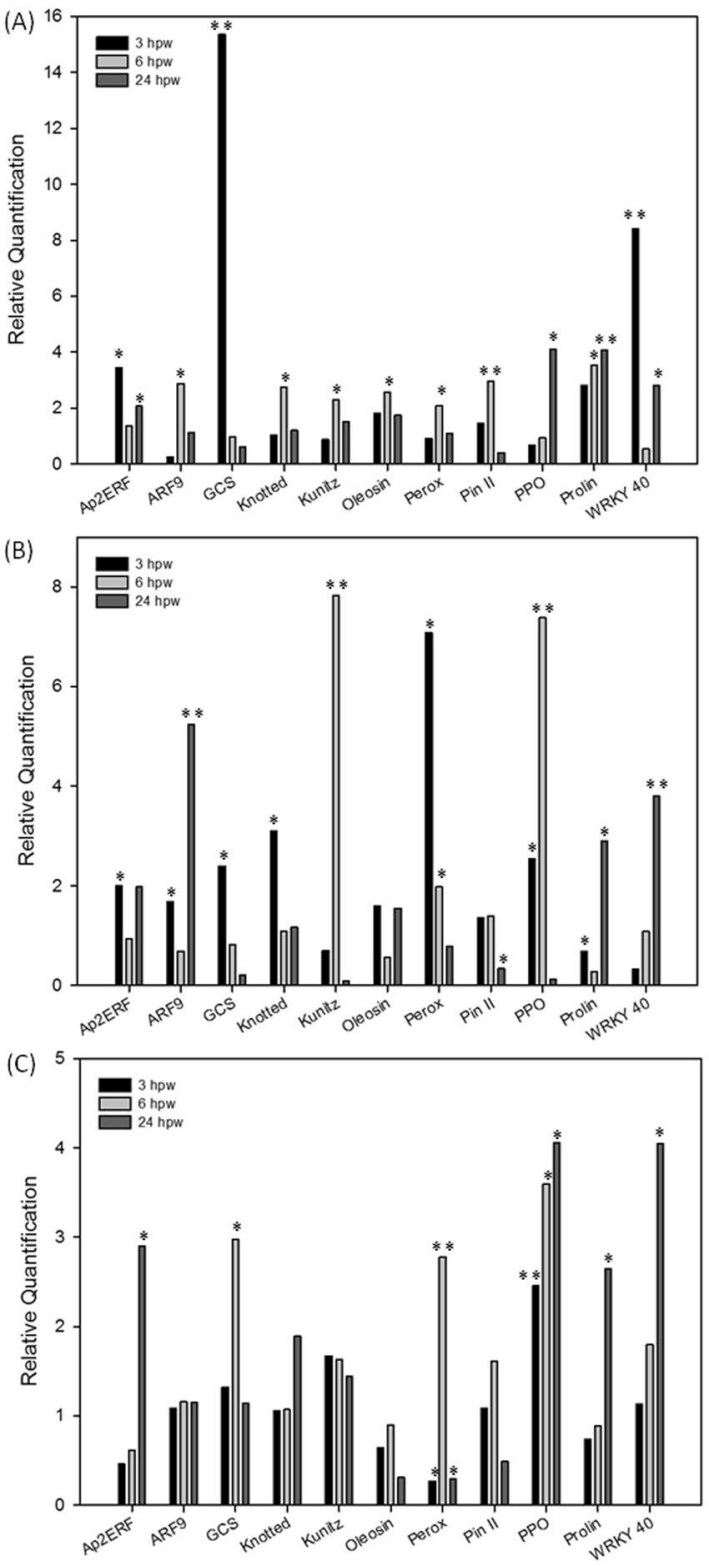
Effect of the exposition to different volatile sources on response to wounding. Time-course expression analysis of defense transcripts by Real Time RT-PCR in receiver plants R-S1 (**A**), R-S2 (**B**) and R-S3 (**C**) 3, 6 and 24 hours following the wounding treatment (WR-S1, WR-S2, WR-S3, respectively). Relative quantities (RQ) are calibrated on WR-S4 samples. Asterisks indicate statistically significant differences compared to control condition (WR-S4) at the same time point (*p < 0.05; **p < 0.01; t-test).

The response to wounding of receivers of the prosystemin overexpressing plants (WR-S2) was significantly higher for 10 out of the 11 genes analyzed in at least one time-point (Fig. 7B). Early genes involved in direct responses to stress (such as peroxidase) were up-regulated 3 hours post wounding and late genes as PPO, Kunitz proteinase inhibitor, prolin and WRKY40 were induced after 6 and/or 24 hours. Germacrene-C-synthase, involved in indirect defenses, was also overexpressed compared to wounded naïve plants (Fig. 7B). The analyzed genes were also up-regulated in wounded plants upon exposition to systemin-treated sources (Fig. 7A). The weakest response, in terms of number of DEGs and expression level, was present in wounded receivers of insect-damaged plants (WR-S3) (Fig. 7C). It is interesting that while most genes were ovexpressed in WR-S3 at the latest time points, receivers of systemin treated or prosystemin overexpressing plants were, in comparison, able to mount a more rapid response.

Overall, the data indicated that the exposure to the three types of sources promotes a stronger activation of direct and indirect defenses compared to control (WR-S4) and that systemin application, overexpression or herbivory induction, can generate for some genes a faster response to wounding in primed plants.

## Discussion

In this study, we used gene expression, behavioral and chemical approaches to determine the effect of the systemin-triggered priming against herbivory in tomato. By using different types of source plants, we demonstrated that systemin, besides activating a systemic defense response^5,6^, is an effective trigger of a priming response in neighboring unchallenged plants. The experimental data showed that systemin can initiate this process, which, is most probably governed by volatile signal molecules generated by systemin dependent metabolic pathways. The identification of the specific active components inducing this response is a very interesting research issue worth of future consideration. The effect of systemin in the activation of a priming response is consistent with the proposed role of systemin as DAMP signal that acts as a broad indicator of tissue integrity^8,22,23^.

The RNA-Seq analysis allowed recognizing the molecular mechanisms of plant-to-plant priming against herbivorous pests at an unprecedented resolution. Many studies have focused on few defensive traits, without addressing the general defensive state of naïve versus primed plants^24^. In tomato, priming involves cellular proteins that are expected to play a central role in signal perception and amplification^25^. For example, RLKs play crucial roles in development and adaptation to stress, including functions for broad-spectrum and elicitor-initiated defense responses. The accumulation of their transcripts is also considered a feature of plant immunity against pathogens^17^. Interestingly, most of the overexpressed kinases were leucine-rich repeat (LRR) proteins, which are known to be used for the detection of biotic stress^26^. The up-regulation of several classes of kinases is consistent with a multiple receptors model of recognition of priming signals^14^. However, mitogen-activated protein kinases involved in systemin-mediated defense responses against herbivores^27^ were not differentially expressed suggesting that the effect of the systemin in plant-to-plant communication is different from an increase of direct defense. Taking into account the number of overexpressed transcripts annotated as kinases (around 10% of overexpressed DEGs) as well as the GSEA result, the data imply that a combination of signaling mechanisms and gene expression patterns characterizes the priming response in tomato. Such attribute may be necessary to translate the complex chemical signals involved in plant-to-plant communication, as in the case of plant immunity. Moreover, the accumulation in primed cells of various classes of receptors and signaling proteins should provide an advantage to cope a future biotic stress.

Once a stress activates receptors, the plant signal transduction cascade navigates the signal by a series of biochemical events, typically culminating in the transport of activated kinases to the nucleus to activate the TFs required for the cellular response. The high number of DEGs putatively involved in regulation of signaling pathways is aligned to the high number of DEGs annotated as TFs (around 5% of up- and 5% of down-regulated genes), because their abundant presence is a requirement for a faster response to biotic stress^28^. Among the overexpressed TFs, five WRKY members (23, 48, 70, 42, 40) were up-regulated in R-S1 and, among the tested genes, WRKY40 was overexpressed in the different types of receivers.

The primed state should be also dictated by epigenetic mechanisms that influence chromatin structure, because they represent a cellular memory in plant adaptation to stress^28^. Among the genes whose products can chemically modify histone tails, only two SET-domain containing histone lysine N-methyltransferases were up-regulated in primed plants. It is tempting to speculate that, in tomato, lysine methylation is an epigenetic mark of priming for insect resistance, similarly to Arabidopsis^29,30^. The data pave the way to future studies to identify genes involved in the transcriptional memory for a faster defense response to herbivory by, for instance, capturing DNA targets for selected histone modifications across the entire genome of primed plants.

Plants treated with Systemin also altered in receiver plants the expression of genes that can directly affect phytophagous pests, mainly proteinase inhibitors and polyphenol oxidases. Their number is lower compared to the tomato response to the endogenous overproduction of prosystemin^8^, indicating that the primed state dictated by the systemin is different from a systemic activation of defense response that occurs in transgenic plants. According to the cost-benefit theory, the primed state should have lower costs than the direct activation of defenses^31,32^, mainly because plant-to-plant priming improves stress response but it does not necessarily confer resistance^14^. For instance, in maize, herbivore-induced plant volatiles did not significantly increase the expression of the trypsin inhibitor in leaves of receiver plants^33^.

The possible biological relevance of the systemin-triggered priming was evaluated using three different types of source plants. An increase of both direct and indirect resistance in receiver plants was observed, indicating that systemin-induced priming can influence organisms of the first (neighboring plants), second (phytophagous pests) and third (parasitoids) trophic level. The qualitative and quantitative differences in VOCs emissions were confirmed by PLSDA analysis and explain the effect on parasitoid attraction. Compared to R-S4, all receiver plants, after 24 hours of exposition to the treated sources, showed an increase in β-ocimene, a VOC known for its role in plant defense^34,35^ and attractiveness towards *A*. *ervi* ^36^. The 2,4-dimethyl-1-heptene was also a dominant compound released by the different receiver plants. This volatile is a powerful elicitor of indirect defense in inter-specific plant-to-plant communication involving tomato and *A*. *ervi* ^37^. Moreover, it was found in the VOCs associated to the host preference by the parasitoid *Microplitis mediator* in *Brassica oleracea* plants^38^. Consistent with the biological effect of the different source plants, the analysis of the wound response of WR-S1, WR-S2 and WR-S3 indicated a faster and more robust increase of gene expression in comparison with wounded naïve receivers (WR-S4). Taking into account the magnitude of the Relative Quantification, receivers exposed to transgenic plants constitutively expressing the systemin precursor displayed the strongest activation.

Priming with defense elicitors is of growing interest in sustainable agriculture, especially for the predicted lower metabolic costs imposed to plants, in comparison to the constitutive elicitation of resistance traits. While a number of natural compounds are considered potential enhancers of plant priming, many have been identified because involved in the improvement of plant performance against phytophatogenic fungi or bacteria^39^. For instance, pre-treatment of maize with the ZmPep1, and of Arabidopsis with flg22 or AtPep1 peptides, increased resistance against microbial pathogens^40,41^. To our knowledge, systemin is the first non-volatile natural compound that significantly increases direct and indirect defense against insect pests in neighboring unchallenged plants^14,42^. In addition to the direct application of the peptide, transgenic plants overexpressing the prosystemin resulted useful for increasing insect resistance in non-transgenic neighbors. For these reasons, the systemin peptide has an interesting potential for Integrated Pest Management approaches and sustainable crop production^42^. Moreover, pre-treatment with systemin could increase tolerance against other biotic or abiotic stresses^8,43^.

In conclusion, our work demonstrated that the up-regulation of genes involved in signaling perception and transcription is a major component of the priming state in tomato. We provided a detailed view of the transcriptomic changes due to plant-to-plant communication, which led to a more comprehensive understanding of priming for insect resistance in a crop. Systemin appears to trigger changes that specifically prime a subset of genes in neighboring plants and, unlike the within-plant communication, it does not induce a large number of genes that can directly deter herbivory. The observed transcriptional differences were associated to a more rapid, robust defense response to wounding, and with a significant increase of direct and indirect defense against larvae.

Nowadays, small peptides are considered fundamental signaling molecules in many plant processes^44^ and our work extends the range of downstream effects of this class of molecules to intraspecific plant-to-plant communication. The observed enhancement of plant defense may allow a more efficient use of systemin for the development of pest control strategies based on the manipulation of plant physiology.

## Methods

### Peptide synthesis

The systemin peptide was obtained by solid phase synthesis following standard protocols^45^ on the Rink Amide MBHA resin, (loading 0.65 mmol/g). Purification of the peptides was carried out by RP-HPLC on a semipreparative column (Jupiter 10 µ, Proteo 90 Å, 250 × 10 mm) using a gradient of acetonitrile (0.1% trifluoroacetic acid, TFA) in water (0.1% TFA) from 5 to 50% in 30 min at 5 ml/min. Peptides were characterized by mass spectrometry. Sequences and mass spectrometry data follow.

Systemin sequence: AVQSKPPSKRDPPKMQTD. Mass calculated (Da): 2009.3 Mass spectrum fragmentation data (Da): 670.94 [M + 3 H]^3+^; 1005.60 [M + 2 H]^2+^.

### Stability assay

The systemin peptide was dissolved in acetic acid/sodium acetate buffer 10 mM at pH 5 and in phosphate buffer 10 mM pH 7 at a 249 µM concentration. Forty-five µg of peptide were withdrawn after 6, 24 and 48 hours of incubation and analyzed by LC-MS using a gradient of acetonitrile (0.1% TFA) in water (0.1% TFA) from 5 to 50% in 30 min at 0.8 ml/min on a Phenomenex Jupiter 4 µ Proteo 90 Å (150 × 4.60 mm) column.

### Plant material and treatments

Tomato seeds (*Solanum lycopersicum* L. cv. ‘Red Setter’) were surface-sterilized with sodium hypochlorite, rinsed, washed with 70% ethanol and rinsed five times with sterile distilled water. Seeds were germinated on wet sterile paper in the dark in an environmental chamber at 24 °C. Upon root emergence, plantlets were transferred to sterile soil in an environmental chamber at 26 ± 2 °C with a photoperiod of 16/8 h light/dark.

We used the following experimental setups to study the role of systemin in communication between source (S) and receiver (R) plants. Source plants 1 (S1) were obtained by foliar application of the systemin peptide. Four weeks-old tomato plants were treated with a 100 pM Sys solution in 1X PBS buffer. Fifteen spots of two µl of peptide solution were gently placed on the abaxial surface of fully expanded healthy leaves (a mock treatment without peptide was used for the controls). Source plants 2 (S2) were transgenic tomatoes overexpressing the prosystemin^8^. In these plants, the prosystemin cDNA is expressed under the control of the 35 S RNA promoter. Tomato plants attacked by phytophagous pests were the third type of source plants (S3). Chewed plants were obtained by allowing *Spodoptera littoralis* (Noctuidae) fourth instar larvae to feed on four weeks-old plants for one hour. Source plants 4 (S4) were untreated tomato plants.

Soon after the above mentioned treatments, receivers (R) and source plants (S1, S2, S3 or S4) were left in a 1:1 ratio in air-tight transparent vessels, where no contact between the two set of plants was allowed. Receiver plants were named R-S1, R-S2, R-S3 or R-S4, respectively (Table 1). We used three biological replicates per experimental thesis for a total of 12 receiver plants exposed for 24 hours to each source. Vessels remained closed during the whole exposition.

For wounding experiments, following the exposition to source plants for 24 hours, R-S1, R-S2, R-S3 and R-S4 plants were injured (approximately a 2 cm-long wound) with a sterile needle on the abaxial surface of the third fully expanded leaf. Wounded samples were named WR-S1, WR-S2, WR-S3 and WR-S4 according to the type of receiver plants (Table 1). Samples were collected 3, 6 and 24 hours upon wounding for the gene expression analysis by Real Time RT-PCR. Experiments were carried out in triplicate, using three plants per experimental thesis.

### RNA-seq

Total RNA was extracted using Plant RNeasy mini kit (Qiagen) according to manufacturer’s protocol. Samples were analyzed with the 2100 Bioanalyzer system (Agilent Technologies) for sizing, quantitation and quality control of RNA. Only samples with a 260/280 nm absorbance >1.8 and a 260/230 nm absorbance> 2 were processed for NGS. Three biological replicates were used for tests and controls. Eight μg of total RNA for each sample were used for library preparation and paired-end sequencing with an Illumina HiSeq. 2500 platform as previously described^46^.

Quality control on raw data was performed using FastQC software. Filtering of adapters, primers and low quality sequences was performed using Trimmomatic. Reads were mapped on tomato genome using the Spliced Transcripts Alignment to a Reference (STAR) software. Statistics for unmapped, poorly and accurately mapped reads were obtained using SAMSTAT software. Reads count was performed by using FeatureCounts. Differentially expressed genes (DEGs) were identified and clustered according to expression profiles using the ‘EdgeR’ package of the R statistical software, filtering DEGs with a fold change (FC) >|1.5| and a p-value> 0.01. Validation of selected DEGs was carried out by Real Time RT-PCR (qRT-PCR).

### Functional annotation

Functional annotation (e.g. statements describing gene functions using GO-terms) of differentially expressed genes was carried out by sequence similarity analysis^47^. Briefly, a BlastX similarity search against the nr NCBI protein database was performed to retrieve a maximum of 20 homologous hits per query. GO-term mapping and annotation were retrieved using NCBI as well as non-redundant reference protein database (PSD, UniProt, Swiss-Prot, TrEMBL, RefSeq, GenPept, PDB Full Gene Ontology DB). Additional annotations (e.g. the recovery of implicit “Biological Process” and “Cellular Component” GO-terms from “Molecular Function” annotations) were implemented using ANNEX. For the “Biological Process” (BP) domain, a leaner overview of the ontology content was achieved by using the GO-plant slim list. Completion of the functional annotation with protein domain information was obtained with InterProScan 5.0. We then removed first level annotations and filtered the GO terms by taxa (Taxonomy ID: 3398, Magnoliophyta (syn: Angiospermae), Genbank common name: flowering plants). Mapping of enzymatic activities into molecular pathways was acquired from the KEGG database. Gene Set Enrichment Analysis was carried out with AgriGO^48^ using as background reference the ITAG 2.4 annotation (FDR < 0.05, Hypergeometric test with Bonferroni correction; minimum number of mapping entries: 5).

### Real Time RT-PCR

The isolation of total RNA from leaves of four-week old plants, the synthesis of the first strand cDNA and real-time PCRs were performed as already reported^49^. Gene expression analysis was carried out using two technical replicates for each of the three biological replicates per samples. Relative quantification of gene expression was carried out using the 2^−ΔΔCt^ method. The statistical significance was evaluated using the two-tailed Student’s t-test for independent samples. The housekeeping gene Elongation Factor 1 alpha (EF-1α) was used as an endogenous reference gene for the normalization of the expression levels of the target genes. Primers and their main features are reported in the Supplementary Table 8.

### Lepidoptera bioassay

*Spodoptera littoralis* (Noctuidae) larvae were grown in an environmental chamber at 25 ± 2 °C, 70 ± 5% RH and fed with an artificial diet composed by 41.4 gl^−1^ wheat germ, 59.2 gl^−1^ brewer’s yeast and 165 gl^−1^ corn meal, supplemented with 5.9 gl^−1^ ascorbic acid, 1.8 gl^−1^ methyl 4-hydroxybenzoate and 29.6 gl^−1^ agar. About 60 newly hatched 1st instar larvae were maintained on this artificial diet and allowed to attain the second instar. Then, experimental groups of 15 uniformly grown 2nd instar larvae were assembled in triplicate and used to assess the larval weight and survival rate as affected by different plant lines. This was done in a no-choice feeding bioassay, by isolating the experimental larvae in a multiwell plastic tray (Bio-Ba-8, Color-Dec, Italy) covered by perforated transparent lids (Bio-Cv-1, Color-Dec Italy) for inspection. Each well was bottom lined with a 2% agar solution (w/v) to create a moist environment required to maintain turgor in the tomato leaf discs employed. These were daily replaced as needed to adequately meet the increasing food requirement of growing larvae. Plastic trays were kept at 28 °C 16:8 hr light/dark photoperiod. Weight and mortality rate of experimental larvae were daily recorded until they attained the pupal stage. Mature larvae (6th instar) were transferred for pupation into plastic boxes containing vermiculite. This set-up was replicated in two independent experiments.

### Flight behavior bioassay

Flight behavior of the parasitoid wasp *Aphidius ervi* (*Braconidae*) was measured as relative attractiveness towards tomato plants in a single-choice wind tunnel bioassay^10^. One hundred and fifty females were tested for each experimental thesis and controls, by releasing them individually in the odour plume 35 cm downwind from the target. Each female was only used once. Parasitoids were observed for a maximum time of 10 min, and flight behavior data were recorded and analyzed with the aid of event-recording software (the Observer, Noldus Information Technology, Wageningen, the Netherlands). Behavioral experiments were conducted on several days, and targets were presented in a random order to reduce the effect of temporal variability on the results. Ten receiver plants per experimental thesis were tested. The percentage of response (oriented flights, landings on the target) to each target was calculated. The number of parasitoids responding to each target was compared to the others by one-way ANOVA.

### Volatile collection and identification

Glassware, silicon, and Teflon connections were heat-sterilized at 100 °C overnight before use. Volatiles from receiver and control plants were collected by an airtight entrainment system immediately after the wind-tunnel assay. One plant was placed in a bell jar (20 l) sealed with Parafilm and connected to a circulation pump whose flow was adjusted at 200 ml per minute. Before re-entering the pump, the air flow passed through an adsorbent trap made of Tenax (SKC, Eighty-Four, PA, USA), connected to the system by a Teflon-capped glass plug. In order to reduce stress to the plant in the system, each collection lasted 3 hr. Air entrainment volatiles were separated by an integrated system including thermal desorber (Tekmar TD-800), gas chromatograph (column: RTX-200, 60 m, 0.25 mm, 0.25 μm, carrier gas: He), and mass spectrometer. The resulting peaks were compared with 36 available standards and compound’s database (National Institute of Standards and Technology, USA). The mean quantities of these compounds collected from 10 plants of each test and control were analysed by One-way ANOVA. PLSDA analysis was performed on the volatile emission of receiver plants.

## Electronic supplementary material

Supplementary information
