# Supplementary material for: Plant-to-plant communication triggered by systemin primes anti-herbivore resistance in tomato
Source: Sci Rep. 2017 Nov 14;7:15522. doi: 10.1038/s41598-017-15481-8 (PMC5686165; doi:10.1038/s41598-017-15481-8)
Supplement: Supplementary file 1 — Supplementary information [file 41598_2017_15481_MOESM1_ESM.pdf]

SUPPLEMENTARY INFORMATION

Plant-to-plant communication triggered by systemin primes anti-herbivore resistance in tomato

Mariangela Coppola[1], Pasquale Cascone[2], Valentina Madonna[1], Ilaria Di Lelio[1], Francesco Esposito[1], Concetta Avitabile[3], Alessandra Romanelli[4], Emilio Guerrieri[2], Alessia Vitiello[1], Francesco Pennacchio[1], Rosa Rao[1,*] & Giandomenico Corrado[1,*]

[1]Dipartimento di Agraria, Università degli Studi di Napoli Federico II, Via Università 100, 80055 Portici, NA, Italy. [2]Istituto per la Protezione Sostenibile delle Piante, CNR, Portici, NA, Italy. [3]Istituto di Biostrutture e Bioimmagini (CNR), via Mezzocannone 16, 80134 Napoli. [4]Dipartimento di Farmacia, Università degli Studi di Napoli Federico II, Via Università 100, 80055 Portici, NA, Italy.

*Correspondence and requests for materials should be addressed to R.R. (email: rao@unina.it) or G.C. (email: giandomenico.corrado@unina.it)

Supplementary Table 1. RNA-seq raw data processing. Three biological replicates (pt) were used for R-S1 and R-S4 plants. See Table 1 for plant code.

Sample	Number of raw reads (sum of pairs)	Number of trimmed reads (sum of pairs)	% Trimmed	Number of Mapped reads	% of Mapping	% of Unique Mapping	Unmapped Reads	% Unmapped reads
R-S4 pt 1	30484562	29676046	97.35	27286820	91.95	98.4	7385	0.02
R-S4 pt 2	62711700	60810566	96.97	56404664	92.75	98.5	13823	0.02
R-S4 pt 3	70072920	68379904	97.58	63844120	93.37	98.4	12858	0.02
R-S1 pt 1	72317900	70590274	97.61	65565860	92.88	98.3	23305	0.03
R-S1 pt 2	76132340	74167434	97.42	69792384	94.10	98.4	19037	0.03
R-S1 pt 3	74669200	71965232	96.38	67566640	93.89	98.4	17347	0.02
Total	386388622	375589456		350460488			93755	
Average	64398103.67	62598242.67	97.22	58410081.33	93.16	98.4	15625.83	0.02
Standard deviation	17268970.45	16768742.77	0.47	15917364.23	0.80	0.06	5520.70	0.00

Supplementary Table 2. Real Time RT-PCR confirmation of the RNA-seq analysis. The data of qRT-PCR are means of three independent biological replicates (each in two technical replicates).

Gene ID	Description	log_2 fold change	
		RNA-seq	Real Time RT-PCR
Solyc04g074900	40S ribosomal protein	1.96	1.55
Solyc08g082630	Auxin response factor 9	1.88	1.28
Solyc04g077210	Knotted-like homeobox protein	3.77	1.19
Solyc03g119820	Oleosin 1	2.41	1.33
Solyc05g046020	Peroxidase 3	1.97	-0.74
Solyc02g089620	Proline dehydrogenase 2	1.88	1.10
Solyc03g033350	Proteinase Inhibitor I	1.36	0.93
Solyc03g020030	Proteinase Inhibitor II	1.96	0.95
Solyc02g078690	Serine carboxypeptidase	2.31	1.96
Solyc09g015770	WRKY 40	1.24	2.09

Supplementary Table 3. List of differentially up-regulated genes identified by RNA-seq upon exposition of receiver plants to Sys-treated plants.

The table reports the locus id, the gene description, the log_2 of the fold change (log2FC), the FDR of the differential expression, the Best Blast Hit Descriptor and the GO IDs.

Locus	Gene Description (ITAG 2.5)	Log2FC	FDR	Best Blast Hit Descriptor	GO ID
Solyc07g064380.2	Serine/threonine-protein phosphatase 7 long form homolog V1 *--- PPP7L_ARATH); contains Interpro domain(s) IPR019557 Aminotransferase-like, plant mobile domain	5.45	3.06E-05	Serine threonine-protein phosphatase 7 long form homolog	
Solyc10g054900.1	Proline-rich protein (AHRD V1 ***- Q9M6T7_NICGL); Interpro domain(s) IPR010616 Protein of unknown function DUF1210	4.99	2.46E-03	Proline-rich protein 4	
Solyc07g040960.1	Os07g0175100 protein (Fragment) (AHRD V1 *---	4.3	2.68E-04	Nuclease harbi1	GO:0004623; GO:0006644; GO:0016042
Solyc10g018190.1	1-aminocyclopropane-1-carboxylate oxidase (AHRD V1 **-- ACCO_MUSAC); Interpro domain(s) IPR005123 Oxoglutarate and iron-dependent oxygenase	4.21	1.12E-03	Hyoscyamine 6-dioxygenase	GO:0016491
Solyc04g077210.2	Knotted-like homeobox protein (AHRD V1 ***- contains Interpro domain(s) IPR005541 KNOX2	3.77	1.52E-08	Homeobox protein knotted-1	GO:0003677; GO:0045449
Solyc06g062560.1	Phosphatase (AHRD V1 ***- D2V497_NAEGR); contains domain(s) IPR016965 Pyridoxal phosphate phosphatase, PHOSPHO2	3.76	1.83E-04	Inorganic pyrophosphatase 1	GO:0016791
Solyc01g090890.2	Xenotropic and polytropic retrovirus receptor (AHRD **-- B2GU54_XENTR); contains Interpro domain(s) IPR004331 SPX, N-terminal	3.4	8.46E-05	Spx domain-containing protein 3	
Solyc01g091590.2	SRC2 protein (AHRD V1 ***- A7XAJ8_9CONI); Interpro domain(s) IPR000008 C2 calcium-dependent membrane targeting	3.34	1.34E-03	Bon1-associated protein 2	GO:0005515; GO:0005543
Solyc08g067230.2	MADS box transcription factor (AHRD V1 A7LK41_POPDE); contains Interpro domain(s) IPR002100 Transcription factor, MADS-box	3.22	2.88E-03	Type ii mads-box transcription partial	GO:0003700; GO:0005634; GO:0006355
Solyc01g094380.2	Glycosyltransferase CAZy family GT90 (Fragment) (AHRD **-- D8SQN9_SELML); contains Interpro domain(s) IPR008539 Protein of unknown function DUF821, CAP10-like	2.93	9.37E-05	O-glucosyltransferase rumi homolog	
Solyc04g074420.1	Phi-1 protein (Fragment) (AHRD V1 **-- contains Interpro domain(s) IPR006766 Phosphate-induced protein 1 conserved region	2.9	1.24E-05	Unknown Protein	
Solyc03g082430.1	Growth-regulating factor 4 (AHRD V1 *--- contains Interpro domain(s) IPR014977 WRC	2.84	2.50E-04	Growth-regulating factor 7	GO:0005634
Solyc03g093560.1	Ethylene-responsive transcription factor 2 (AHRD V1 B6U860_MAIZE); contains Interpro domain(s) IPR001471 Pathogenesis-related transcriptional factor and ERF, DNA-binding	2.76	8.10E-04	Ethylene-responsive transcription factor 5	GO:0003700; GO:0006355
Solyc06g074030.1	CCR4-NOT transcription complex subunit 7 (AHRD ***- B4FG48_MAIZE); contains Interpro domain(s) IPR006941 Ribonuclease CAF1	2.7	1.26E-04	Probable ccr4-associated factor 1 homolog 11	GO:0003676
Solyc04g074440.1	Os06g0220000 protein (Fragment) (AHRD V1 ***- contains Interpro domain(s) IPR006766 Phosphate-induced protein 1 conserved region	2.57	1.25E-04	Protein exordium	
Solyc10g006700.1	Calcium-binding EF hand family protein (Fragment) V1 **-* D7MEH5_ARALY); contains Interpro domain(s) IPR011992 EF-Hand type	2.47	3.47E-04	Calcium-binding protein pbp1	GO:0005509
Solyc03g119820.1	Oleosin Bn-V (AHRD V1 ***- B6UIA7_MAIZE); Interpro domain(s) IPR000136 Oleosin	2.41	1.06E-03	Oleosin 1	GO:0012511; GO:0016021
Solyc06g035530.2	Gibberellin 20-oxidase-2 (AHRD V1 **** Q9ZPP3_SOLLC); Interpro domain(s) IPR005123 Oxoglutarate and iron-dependent oxygenase	2.37	1.80E-06	Gibberellin 20 oxidase 1	GO:0016491
Solyc01g009160.2	Harpin-induced protein-like (Fragment) (AHRD V1 **-- contains Interpro domain(s) IPR010847 Harpin-induced 1	2.33	2.00E-04	Protein yls9	
Solyc02g078690.1	Serine carboxypeptidase K10B2.2 (AHRD V1 **** contains Interpro domain(s) IPR001563 Peptidase S10, serine carboxypeptidase	2.31	6.02E-07	Serine carboxypeptidase	GO:0004185; GO:0006508
Solyc04g014260.1	Zinc finger-homeodomain protein 1 (Fragment) (AHRD **-- B0LK17_YUCFI); contains Interpro domain(s) IPR006456 ZF-HD homeobox protein Cys/His-rich dimerisation region	2.31	4.46E-05	Zinc-finger homeodomain protein 1	GO:0003677; GO:0045449
Solyc11g010710.1	AP2-like ethylene-responsive transcription factor At1g16060 (AHRD *-*- AP2L1_ARATH); contains Interpro domain(s) IPR001471 Pathogenesis-related transcriptional factor and ERF, DNA-binding	2.31	9.10E-05	Ap2-like ethylene-responsive transcription factor ail6	GO:0003700; GO:0006355

Solyc04g074430.1	Phi-1 protein (Fragment) (AHRD V1 *-*- contains Interpro domain(s) IPR006766 Phosphate-induced protein 1 conserved region	2.19	9.80E-05	Protein exordium	
Solyc04g077980.1	Zinc-finger protein (AHRD V1 ***- Q40899_PETHY); Interpro domain(s) IPR007087 Zinc finger, C2H2-type	2.19	9.14E-04	Zinc finger protein zat10	GO:0005622; GO:0008270
Solyc08g083050.1	Unknown Protein (AHRD V1)	2.17	1.81E-03	Unknown Protein	
Solyc08g066890.2	Unknown Protein (AHRD V1)	2.07	4.04E-04	Bark storage protein a	
Solyc01g066160.1	Unknown Protein (AHRD V1)	2.05	5.38E-04	Unknown Protein	
Solyc02g089170.2	Alpha-1 4-glucan-protein synthase (AHRD V1 **-* contains Interpro domain(s) IPR004901 Alpha-1,4-glucan-protein synthase, UDP-forming	2.04	3.98E-04	Alpha- -glucan-protein synthase	GO:0005794; GO:0007047; GO:0009505; GO:0016758; GO:0030054; GO:0030244
Solyc01g007080.2	Aluminum-activated malate transporter (Fragment) (AHRD V1 Q071L4_AVESA); contains Interpro domain(s) IPR006214 Uncharacterised protein family UPF0005	2.02	1.51E-03	Aluminum-activated malate transporter 8	GO:0010044
Solyc10g055740.1	Lysine/histidine transporter (AHRD V1 ***- B9GQ40_POPTR); Interpro domain(s) IPR013057 Amino acid transporter, transmembrane	1.98	2.59E-03	Lysine histidine transporter-	
Solyc05g046020.2	Peroxidase (AHRD V1 **** Q07446_SOLLC); contains domain(s) IPR002016 Haem peroxidase, plant/fungal/bacterial	1.97	3.32E-08	Peroxidase 3	GO:0005515; GO:0055114; GO:0004601
Solyc03g020030.2	Proteinase inhibitor II (AHRD V1 ***- contains Interpro domain(s) IPR003465 Proteinase inhibitor I20, Pin2	1.96	1.89E-05	Proteinase inhibitor type-2 cevi57	GO:0004867
Solyc04g074900.2	40S ribosomal protein S21 (AHRD V1 D7M5J1_ARALY); contains Interpro domain(s) IPR001931 Ribosomal protein S21e	1.96	2.35E-03	40s ribosomal protein s21	GO:0003735; GO:0005622; GO:0005840; GO:0006412
Solyc04g049920.2	RNA binding protein-like (AHRD V1 **-- contains Interpro domain(s) IPR000504 RNA recognition motif, RNP-1	1.94	5.51E-04	Rna-binding protein 38	GO:0000166
Solyc02g089620.2	Proline dehydrogenase (AHRD V1 **** A1E289_ACTDE); Interpro domain(s) IPR015659 Proline oxidase	1.88	4.36E-09	Proline dehydrogenase mitochondrial	GO:0004657; GO:0005515; GO:0055114
Solyc08g082630.2	Auxin response factor 9 (AHRD V1 D7R605_SOLLC); contains Interpro domain(s) IPR010525 Auxin response factor	1.88	4.96E-10	Auxin response factor 9	GO:0003677; GO:0005634; GO:0009725; GO:0045449
Solyc08g068600.2	Decarboxylase family protein (AHRD V1 ***- contains Interpro domain(s) IPR002129 Pyridoxal phosphate-dependent decarboxylase	1.84	1.99E-04	Histidine decarboxylase	GO:0003824; GO:0030170
Solyc11g013220.1	Os04g0690100 protein (Fragment) (AHRD V1 ***-	1.84	3.01E-05	C2h2-like zinc finger protein	GO:0005622; GO:0008270
Solyc04g074450.1	Phi-1 protein (Fragment) (AHRD V1 ***- contains Interpro domain(s) IPR006766 Phosphate-induced protein 1 conserved region	1.82	1.42E-04	Protein exordium	
Solyc06g007180.2	Asparagine synthase (Glutamine-hydrolyzing) (AHRD V1 **** contains Interpro domain(s) IPR006426 Asparagine synthase, glutamine-hydrolyzing	1.81	2.30E-03	Asparagine synthetase	GO:0004066; GO:0006529; GO:0042803
Solyc02g069280.2	ARGONAUTE 1 (AHRD V1 ***- D6RUV9_TOBAC); Interpro domain(s) IPR003165 Stem cell self-renewal protein Piwi	1.78	1.98E-03	Protein argonaute 2	GO:0003676
Solyc06g062540.2	Phosphatase (AHRD V1 ***- D2V497_NAEGR); contains domain(s) IPR016965 Pyridoxal phosphate phosphatase, PHOSPHO2	1.77	5.22E-04	Inorganic pyrophosphatase 1	GO:0016791
Solyc03g005910.2	GDSL esterase/lipase At1g29670 (AHRD V1 ***- contains Interpro domain(s) IPR001087 Lipase, GDSL	1.73	4.66E-05	Gdsl esterase lipase	GO:0006629; GO:0016788
Solyc07g041640.2	Growth-regulating factor 1 (AHRD V1 *--- contains Interpro domain(s) IPR014977 WRC	1.7	1.10E-04	Growth-regulating factor 1-	GO:0005634
Solyc08g066880.2	5&apos-methylthioadenosine/S-adenosylhomocysteine nucleosidase (AHRD V1 *-*- D6E0Z8_9FIRM); Interpro domain(s) IPR018017 Nucleoside phosphorylase, family 1	1.69	1.49E-03	Bark storage protein a-	GO:0009116
Solyc00g206460.1	Os06g0220000 protein (Fragment) (AHRD V1 **-- contains Interpro domain(s) IPR006766 Phosphate-induced protein 1 conserved region	1.68	3.34E-04	Unknown Protein	
Solyc04g074340.2	UDP-glucuronosyltransferase (AHRD V1 **-* Q5UB81_ARATH); contains domain(s) IPR002213 UDP-glucuronosyl/UDP-glucosyltransferase	1.68	1.96E-03	7-deoxyloganetin glucosyltransferase-	GO:0008152; GO:0016758
Solyc01g103500.2	Unknown Protein (AHRD V1)	1.67	5.80E-04	Extended synaptotagmin-3	
Solyc06g053870.2	Fatty acid elongase 3-ketoacyl-CoA synthase (AHRD **** Q6DUV6_BRANA); contains Interpro domain(s) IPR012392 Very-long-chain 3-ketoacyl-CoA synthase	1.66	1.25E-06	3-ketoacyl- synthase 19	GO:0003824; GO:0008152
Solyc11g042880.1	Harpin-induced protein (AHRD V1 **-- B6TDG9_MAIZE)	1.66	3.28E-05	Unknown protein	
Solyc03g115120.1	Chaperone protein dnaJ (AHRD V1 ***- contains Interpro domain(s) IPR015609 Molecular chaperone, heat shock protein, Hsp40, DnaJ	1.65	1.72E-07	Dnaj homolog subfamily c member 21	GO:0031072

Solyc08g074620.1	Polyphenol oxidase (AHRD V1 ***- Q41428_SOLTU); Interpro domain(s) IPR016213 Polyphenol oxidase, plant	1.65	8.78E-16	Polyphenol oxidase	GO:0004097; GO:0055114
Solyc02g030460.2	Sh4 homologue protein (AHRD V1 *-*- contains Interpro domain(s) IPR017877 MYB-like	1.64	4.53E-04	Methyl- -binding protein 2-like isoform	GO:0003700
Solyc07g055180.2	Receptor protein kinase-like protein (AHRD V1 Q9LJU5_ARATH); contains Interpro domain(s) IPR002290 Serine/threonine protein kinase	1.61	8.12E-05	Serine threonine-protein kinase cdl1	GO:0004672; GO:0005524; GO:0006468
Solyc10g007800.2	Meiosis 5 (AHRD V1 *-*- B6T5X0_MAIZE)	1.6	9.49E-04	Protodermal factor 1	
Solyc04g078460.2	N(4)-(Beta-N-acetylglucosaminyl)-L-asparaginase (AHRD V1 **** ASPG_ELIMR); contains domain(s) IPR000246 Peptidase T2, asparaginase 2	1.58	7.24E-04	Probable isoaspartyl peptidase l-asparaginase 2	GO:0016787
Solyc10g087030.1	cytochrome P450	1.58	3.15E-04	Premnaspirodiene oxygenase	n/a
Solyc00g138060.2	2-oxoglutarate-dependent dioxygenase (AHRD V1 **** B9GL08_POPTR); Interpro domain(s) IPR005123 Oxoglutarate and iron-dependent oxygenase	1.56	9.87E-04	Unknown Protein	GO:0016491
Solyc04g050570.2	GDSL esterase/lipase At5g33370 (AHRD V1 ***- contains Interpro domain(s) IPR001087 Lipase, GDSL	1.56	9.79E-08	Gdsl esterase lipase	GO:0006629; GO:0016788
Solyc08g083240.2	Calmodulin-binding protein family-like (AHRD V1 **-* contains Interpro domain(s) IPR000048 IQ calmodulin-binding region	1.56	5.76E-08	Protein iq-domain 1	GO:0005516
Solyc01g007010.2	U-box domain-containing protein (AHRD V1 ***- contains Interpro domain(s) IPR003613 U box domain	1.55	2.05E-03	E3 ubiquitin-protein ligase pub22	GO:0005488
Solyc10g048190.1	Ubiquinol-Cytochrome c reductase iron-sulfur subunit (AHRD **-- B6B5V9_9RHOB); contains Interpro domain(s) IPR002740 Uncharacterised protein family UPF0310	1.55	1.46E-04	Thymocyte nuclear protein 1	
Solyc10g006660.2	Calcium-binding EF hand family protein (Fragment) V1 **-- D7MEH5_ARALY); contains Interpro domain(s) IPR011992 EF-Hand type	1.54	2.47E-03	Calcium-binding protein pbp1	GO:0005509
Solyc01g007200.2	Unknown Protein (AHRD V1)	1.53	3.92E-04	Unknown protein	
Solyc11g011310.1	Rhamnogalacturonate lyase (AHRD V1 ***- A3XLZ2_LEEBM); Interpro domain(s) IPR010325 Rhamnogalacturonate lyase	1.53	8.24E-05	Probable rhamnogalacturonate lyase b	GO:0030246
Solyc03g122360.2	Cytochrome P450	1.51	1.29E-04	Cytochrome p450 71a1	n/a
Solyc04g024840.2	GDSL esterase/lipase 1 (AHRD V1 **** contains Interpro domain(s) IPR001087 Lipase, GDSL	1.51	1.25E-03	Unknown Protein	GO:0006629; GO:0016788
Solyc10g074440.1	Endochitinase (Chitinase) (AHRD V1 **** Q43184_SOLTU); Interpro domain(s) IPR016283 Glycoside hydrolase, family 19	1.51	7.74E-10	Endochitinase (Chitinase)	GO:0008061
Solyc01g089910.2	Flotillin domain protein (AHRD V1 ***- contains Interpro domain(s) IPR001107 Band 7 protein	1.5	2.65E-03	Flotillin-like protein 6	
Solyc01g106910.2	Unknown Protein (AHRD V1)	1.49	1.73E-05	Unknown protein	
Solyc07g055750.2	Strictosidine synthase-like protein (AHRD V1 ***- contains Interpro domain(s) IPR004141 Strictosidine synthase	1.49	1.15E-03	Strictosidine synthase 1	GO:0004064; GO:0005515; GO:0009058
Solyc02g033040.2	F-box family protein (AHRD V1 ***- contains Interpro domain(s) IPR001810 Cyclin-like F-box	1.48	3.78E-04	F-box kelch-repeat protein	
Solyc02g078850.1	Unknown Protein (AHRD V1)	1.47	9.28E-08	Shematrin-like protein 2	
Solyc01g098620.2	Unknown Protein (AHRD V1)	1.46	8.76E-04	Unknown protein	
Solyc08g066220.2	Decarboxylase family protein (AHRD V1 *-*- contains Interpro domain(s) IPR002129 Pyridoxal phosphate-dependent decarboxylase	1.46	3.04E-04	Histidine decarboxylase	GO:0003824; GO:0030170
Solyc04g009040.2	Receptor like kinase, RLK	1.45	1.97E-04	Probable lrr receptor-like serine threonine-protein kinase	GO:0005515; GO:0019199; GO:0006468
Solyc03g034390.1	Lipid transfer protein (AHRD V1 --*- contains Interpro domain(s) IPR013770 Plant lipid transfer protein and hydrophobic protein, helical	1.44	1.67E-04	Non-specific lipid-transfer protein 2	GO:0006869
Solyc04g081080.1	Receptor like kinase, RLK	1.44	2.13E-04	Probable lrr receptor-like serine threonine-protein kinase	GO:0004675; GO:0006468
Solyc09g098010.2	Cytochrome P450	1.44	2.26E-03	Geraniol 8-hydroxylase	GO:0004497; GO:0005506; GO:0020037
Solyc09g072750.2	Unknown Protein (AHRD V1)	1.43	7.32E-08	Repetitive proline-rich cell wall protein 2	

Solyc03g114560.2	Strictosidine synthase family protein (AHRD V1 D7LVM7_ARALY); contains Interpro domain(s) IPR004141 Strictosidine synthase	1.42	1.27E-05	Strictosidine synthase 3	GO:0004064; GO:0005515; GO:0009058
Solyc06g071640.2	Alliinase (Fragment) (AHRD V1 ***- Q94EJ8_ALLFI); Interpro domain(s) IPR006948 Allinase, C-terminal	1.42	6.09E-04	Tryptophan aminotransferase-related protein 2	GO:0016846
Solyc10g083170.1	2,5-dichloro-2,5-cyclohexadiene-1,4-diol dehydrogenase (AHRD V1 **** LINC_PSEPA); Interpro domain(s) IPR002347 Glucose/ribitol dehydrogenase	1.42	5.99E-05	Secoisolariciresinol dehydrogenase	GO:0008152; GO:0016491
Solyc03g007690.1	ABC transporter G family member 8 V1 ***- AB8G_ARATH); contains Interpro domain(s) IPR013525 ABC-2 type transporter	1.41	7.95E-06	Abc transporter g family member 8	GO:0005524; GO:0016887
Solyc07g052980.2	Xyloglucan endotransglucosylase/hydrolase 5 (AHRD V1 **** contains Interpro domain(s) IPR016455 Xyloglucan endotransglucosylase/hydrolase	1.41	4.34E-05	Xyloglucan endotransglucosylase hydrolase protein 9	GO:0004553; GO:0005975
Solyc09g082790.2	DNA repair and recombination protein RAD51 V1 **-- A9CT16_ENTBH); contains Interpro domain(s) IPR011940 Meiotic recombinase Dmc1	1.41	1.06E-05	Meiotic recombination protein dmc1 homolog	GO:0006259
Solyc12g013700.1	Aluminum-induced protein-like protein (AHRD V1 ***-	1.41	1.55E-03	Stem-specific protein tsjt1	
Solyc04g077170.2	EPIDERMAL PATTERNING FACTOR-like protein 2 (AHRD ***- EPFL2_ARATH)	1.4	2.76E-05	Epidermal patterning factor-like protein 2	
Solyc05g005670.1	U-box domain-containing protein (AHRD V1 *--- contains Interpro domain(s) IPR011989 Armadillo-like helical	1.4	1.89E-03	U-box domain-containing protein 19	GO:0005488
Solyc01g066640.2	Os04g0405500 protein (Fragment) (AHRD V1 *-*-	1.38	6.92E-06	Uncharacterized serine-rich	
Solyc01g079980.2	Xylanase inhibitor (Fragment) (AHRD V1 **-- contains Interpro domain(s) IPR001461 Peptidase A1	1.38	2.85E-04	Basic 7s globulin	GO:0004190; GO:0006508
Solyc06g061230.2	Unknown Protein (AHRD V1); contains Interpro IPR009632 Putative metallocarboxypeptidase inhibitor	1.38	5.92E-05	Metallocarboxypeptidase inhibitor	
Solyc06g072310.2	Homeobox-leucine zipper protein PROTODERMAL FACTOR 2 V1 **** PDF2_ARATH); contains Interpro domain(s) IPR002913 Lipid-binding START	1.38	2.08E-06	Homeobox-leucine zipper protein hdg11	GO:0003677; GO:0045449
Solyc11g072980.1	3-ketoacyl-CoA synthase 6 (AHRD V1 ***- contains Interpro domain(s) IPR012392 Very-long-chain 3-ketoacyl-CoA synthase	1.38	2.44E-05	3-ketoacyl- synthase 3	GO:0003824; GO:0008152
Solyc07g056460.2	Glutathione S-transferase-like protein (AHRD V1 **** contains Interpro domain(s) IPR004046 Glutathione S-transferase, C-terminal	1.37	4.28E-04	lactoperoxidase	GO:0004364
Solyc03g033350.2	Aspartyl protease family protein (AHRD V1 D7LTT7_ARALY)	1.36	2.32E-03	Aspartic proteinase- protein 1	
Solyc04g071030.1	U-box domain-containing protein (AHRD V1 ***- contains Interpro domain(s) IPR003613 U box domain	1.36	1.17E-04	U-box domain-containing protein 28	GO:0005488
Solyc01g057320.1	Kinesin-like protein (AHRD V1 ***- Q75LL2_ORYSJ); Interpro domain(s) IPR001752 Kinesin, motor region	1.35	5.92E-05	Phragmoplast orienting kinesin-1	GO:0003777; GO:0005524; GO:0007018
Solyc01g111350.2	Nodulin family protein (AHRD V1 ***- contains Interpro domain(s) IPR010658 Nodulin-like	1.35	2.50E-04	Probable transporter mch1	
Solyc04g077140.2	Unknown Protein (AHRD V1); contains Interpro IPR010410 Protein of unknown function DUF1005	1.34	4.08E-08	Formin-like protein 18	
Solyc09g064230.1	Phosphoribosylanthranilate transferase (Fragment) (AHRD V1 *--- contains Interpro domain(s) IPR013583 Phosphoribosyltransferase C-terminal, plant	1.34	2.16E-03	Multiple c2 and transmembrane domain-containing protein 1	
Solyc07g007860.1	Proline rich protein (Fragment) (AHRD V1 P93274_MALDO); contains Interpro domain(s) IPR013770 Plant lipid transfer protein and hydrophobic protein, helical	1.32	2.68E-06	Kda proline-rich	GO:0006869
Solyc07g056400.1	Ribosomal protein S6 kinase alpha-6 (AHRD *-** KS6A6_HUMAN); contains Interpro domain(s) IPR002290 Serine/threonine protein kinase	1.32	1.13E-03	Serine threonine-protein kinase wag1	GO:0006468; GO:0004711
Solyc09g006010.2	Pathogenesis related protein PR-1 (AHRD V1 Q9SC15_SOLTU); contains Interpro domain(s) IPR001283 Allergen V5/Tpx-1 related	1.32	1.94E-08	Pathogenesis-related leaf protein 4	GO:0005576
Solyc01g091010.2	YABBY-like transcription factor CRABS CLAW-like protein V1 **-* Q6SRZ7_ANTMA); contains Interpro domain(s) IPR006780 YABBY protein	1.31	3.90E-04	Axial regulator yabby 1	GO:0003677; GO:0005634
Solyc09g072630.2	LRR receptor-like serine/threonine-protein kinase, RLP	1.31	1.41E-03	Piriformospora indica-insensitive protein 2	GO:0005515
Solyc00g156980.2	Choline dehydrogenase (AHRD V1 **-- A5L8M4_9GAMM); Interpro domain(s) IPR008671 Lycopene beta and epsilon cyclase	1.3	7.41E-08	Unknown Protein	GO:0016117; GO:0016705
Solyc01g095750.2	Long-chain-fatty-acid-CoA ligase (AHRD V1 **** B2WS80_ARAHA); Interpro domain(s) IPR000873 AMP-dependent synthetase and ligase	1.3	2.13E-03	Long chain acyl- synthetase 4	GO:0003824; GO:0008152
Solyc03g113970.2	Calmodulin binding protein (AHRD V1 **** contains Interpro domain(s) IPR012416 Calmodulin binding protein-like	1.3	2.29E-03	Calmodulin binding isoform 2	GO:0005516

Solyc09g005630.2	Os03g0291800 protein (Fragment) (AHRD V1 **-- contains Interpro domain(s) IPR004253 Protein of unknown function DUF231, plant	1.3	1.36E-04	Protein trichome birefringence	
Solyc01g008600.2	Zinc finger CCCH domain-containing protein 53 V1 ***- C3H53_ORYSJ); contains Interpro domain(s) IPR000571 Zinc finger, CCCH-type	1.29	1.63E-06	Zinc finger ccch domain-containing protein 53	GO:0000166
Solyc04g074310.2	RNA-binding protein 68390-68829 (AHRD V1 *--- contains Interpro domain(s) IPR000504 RNA recognition motif, RNP-1	1.29	4.12E-06	Rna-binding protein 38	GO:0000166
Solyc05g051290.2	High mobility group family (AHRD V1 B9H3A3_POPTR); contains Interpro domain(s) IPR017956 AT hook, DNA-binding, conserved site	1.29	1.74E-06	Hmg-y-related protein a	GO:0000786; GO:0003677; GO:0005634; GO:0006334
Solyc10g007830.1	LRR receptor-like serine/threonine-protein kinase, RLP	1.29	6.72E-04	Protein too many mouths	GO:0005515
Solyc12g019480.1	Formin 3 (AHRD V1 *-*- D0QAN4_ARATH); Interpro domain(s) IPR015425 Actin-binding FH2	1.29	1.14E-04	Formin-like protein 2	GO:0030036
Solyc02g080330.2	Cytochrome P450	1.28	7.14E-07	Cytochrome p450 77a1	n/a
Solyc07g049430.2	GDSL esterase/lipase At2g42990 (AHRD V1 ***- contains Interpro domain(s) IPR001087 Lipase, GDSL	1.28	1.51E-05	Gdsl esterase lipase	GO:0006629; GO:0016788
Solyc07g054950.1	Cyclin D2 (AHRD V1 **** Q71FH0_WHEAT); Interpro domain(s) IPR015451 Cyclin D	1.28	5.52E-06	Cyclin-d6-1	GO:0005634; GO:0004693
Solyc08g008020.1	Phosphoribosylanthranilate transferase (Fragment) (AHRD V1 *--- contains Interpro domain(s) IPR013583 Phosphoribosyltransferase C-terminal, plant	1.28	5.92E-05	Multiple c2 and transmembrane domain-containing protein 1	
Solyc10g084290.1	Interactor of constitutive active ROPs 3 V1 ***- ICR3_ARATH)	1.27	1.86E-05	Interactor of constitutive active rops chloroplastic-like isoform	
Solyc12g056190.1	Nuclear transport factor 2 (NTF2)-like protein V1 ***- Q67TV5_ORYSJ)	1.27	1.45E-03	Nuclear transport factor 2 family protein	
Solyc01g066910.2	PVR3-like protein (AHRD V1 ***- B6TYX6_MAIZE); Interpro domain(s) IPR003612 Plant lipid transfer protein/seed storage/trypsin-alpha amylase inhibitor	1.26	5.74E-08	Lipid-transfer protein dir1	GO:0006869
Solyc01g111740.2	Bzip-like transcription factor-like (AHRD V1 ***- contains Interpro domain(s) IPR006867 Protein of unknown function DUF632	1.26	1.98E-04	Unknown Protein	
Solyc03g006800.1	TCP family transcription factor (AHRD V1 A6MCZ2_9ORYZ); contains Interpro domain(s) IPR005333 Transcription factor, TCP	1.26	2.73E-04	Transcription factor tcp9	GO:0003700
Solyc09g062970.1	Unknown Protein (AHRD V1)	1.26	4.43E-06	Unknown Protein	
Solyc03g113910.2	Gibberellin-regulated protein 2 (AHRD V1 *--- contains Interpro domain(s) IPR003854 Gibberellin regulated protein	1.25	1.14E-03	Gibberellin-regulated protein 12	
Solyc10g008440.2	Expansin B1 (AHRD V1 ***- C8CC40_RAPSA); Interpro domain(s) IPR007117 Pollen allergen/expansin, C-terminal	1.25	3.86E-04	Expansin beta isoform 1	GO:0005576
Solyc03g116740.2	Genomic DNA chromosome 3 P1 clone (AHRD V1 ***- Q9LVZ7_ARATH)	1.24	9.90E-06	Phosphatidylcholine:diacylglycerol cholinephosphotransferase 1	GO:0004142
Solyc09g015770.2	WRKY transcription factor 6 (AHRD V1 A7UGD3_SOLTU); contains Interpro domain(s) IPR003657 DNA-binding WRKY	1.24	1.88E-03	Probable wrky transcription factor 70	GO:0003700; GO:0005634; GO:0043565; GO:0045449
Solyc09g092720.2	Unknown Protein (AHRD V1); contains Interpro IPR010800 Glycine rich	1.24	6.09E-04	Glycine-rich protein 3 short isoform	
Solyc11g069960.1	Receptor like kinase, RLK	1.24	8.91E-10	Probable leucine-rich repeat receptor-like protein kinase	GO:0006468
Solyc01g111950.2	Receptor-like kinase (AHRD V1 *-*- A7VM42_MARPO); Interpro domain(s) IPR002290 Serine/threonine protein kinase	1.23	5.09E-04	Receptor-like cytosolic serine threonine-protein kinase rbk1 isoform	GO:0004674; GO:0006468
Solyc10g080430.1	Phosphoribosylanthranilate transferase (Fragment) (AHRD V1 *--- contains Interpro domain(s) IPR013583 Phosphoribosyltransferase C-terminal, plant	1.23	2.42E-03	Multiple c2 and transmembrane domain-containing protein 1	GO:0005509
Solyc12g010800.1	BZIP transcription factor family protein (AHRD ***- D7LW03_ARALY); contains Interpro domain(s) IPR011700 Basic leucine zipper	1.23	1.97E-05	Basic leucine zipper 61	GO:0003700; GO:0005634; GO:0006355; GO:0043565; GO:0046983
Solyc02g087510.2	Ribosomal protein S6 kinase alpha-2 (AHRD *-** KS6A2_HUMAN); contains Interpro domain(s) IPR002290 Serine/threonine protein kinase	1.22	1.79E-03	Serine threonine-protein kinase d6pkl2	GO:0006468; GO:0004711
Solyc03g121600.2	Choline dehydrogenase (AHRD V1 ***- B0WJG6_CULQU); Interpro domain(s) IPR012132 Glucose-methanol-choline oxidoreductase	1.22	8.97E-06	Protein hothead-like isoform	GO:0016614
Solyc04g005600.1	MYB transcription factor (AHRD V1 *--- contains Interpro domain(s) IPR015495 Myb transcription factor	1.22	4.81E-05	Protein odorant1	GO:0045449

Solyc02g085490.1	Os12g0581300 protein (Fragment) (AHRD V1 *-*- contains Interpro domain(s) IPR006873 Protein of unknown function DUF620	1.21	5.79E-05	Spindle assembly abnormal protein 6	
Solyc06g060860.1	Unknown Protein (AHRD V1)	1.21	1.73E-03	Atp-dependent caseinolytic protease crotonase family protein	
Solyc08g080590.2	Osmotin 81 (Fragment) (AHRD V1 **-- contains Interpro domain(s) IPR001938 Thaumatin, pathogenesis-related	1.21	6.78E-06	Osmotin-like protein	
Solyc09g098490.2	Epsin 2 (AHRD V1 *-*- Q5NCM7_MOUSE); Interpro domain(s) IPR001026 Epsin, N-terminal	1.21	4.50E-05	Clathrin interactor epsin 2 isoform	GO:0005515; GO:0005488
Solyc02g080610.2	Ankyrin repeat domain-containing protein 13C-A (AHRD **** C0H9Z7_SALSA); contains Interpro domain(s) IPR002110 Ankyrin	1.2	3.85E-05	Ankyrin repeat domain-containing protein 13c	GO:0030165
Solyc02g090960.1	Rapid alkalinization factor 3 (AHRD V1 Q6TF27_SOLCH); contains Interpro domain(s) IPR008801 Rapid ALkalinization Factor	1.2	2.75E-06	Protein ralf	GO:0004871
Solyc06g065340.1	Unknown Protein (AHRD V1)	1.2	2.31E-05	Unknown Protein	
Solyc06g072290.2	Protein Kinase interacting protein (AHRD V1 B6TI58_MAIZE); contains Interpro domain(s) IPR011684 KIP1-like	1.2	1.56E-04	Golgin candidate 5	
Solyc07g056360.1	Unknown Protein (AHRD V1)	1.2	1.42E-03	Transcription factor par1	
Solyc10g008930.1	Glutaredoxin (AHRD V1 ***- B9H2P7_POPTR); contains domain(s) IPR011905 Glutaredoxin-like, plant II	1.2	5.20E-05	Glutaredoxin-c9	GO:0045454; GO:0005515
Solyc10g080870.2	Cytochrome P450	1.2	1.00E-05	Cytochrome p450 86b1	GO:0004497; GO:0005506; GO:0020037
Solyc12g043030.1	Sulfate transporter (AHRD V1 ***- A1YKF8_BRASY)	1.2	5.21E-04	Probable sulfate transporter	GO:0008271; GO:0008272; GO:0016020
Solyc01g097110.1	Fanconi anemia group D2 protein (AHRD *-*- FACD2_HUMAN)	1.19	9.49E-05	Fanconi anemia group d2 protein homolog	
Solyc04g063390.2	Chaperone protein dnaJ 10 (AHRD V1 B4G111_MAIZE); contains Interpro domain(s) IPR003095 Heat shock protein DnaJ	1.19	1.06E-05	Chaperone protein dnaj 10	GO:0031072
Solyc06g007890.2	Gibberellin regulated protein (AHRD V1 **-- contains Interpro domain(s) IPR003854 Gibberellin regulated protein	1.19	1.38E-04	Gibberellin-regulated protein 5	GO:0005515
Solyc08g061910.2	Unknown Protein (AHRD V1)	1.19	1.08E-05	Trihelix transcription factor gt-2	
Solyc01g107730.2	Cyclin (AHRD V1 ***- Q1XGF1_TOBAC); contains domain(s) IPR006671 Cyclin, N-terminal	1.18	2.84E-04	D-type cyclin family 3 subgroup 2	GO:0005634; GO:0005516; GO:0046982
Solyc09g010960.2	WRKY transcription factor 17 (AHRD V1 Q7E0X2_ORYSJ); contains Interpro domain(s) IPR003657 DNA-binding WRKY	1.18	2.02E-03	Probable wrky transcription factor 49	GO:0003700; GO:0005634; GO:0043565; GO:0045449
Solyc01g089850.2	Cyclin-dependent protein kinase regulator Pho80 (AHRD **** B6QI37_PENMQ); contains Interpro domain(s) IPR012389 Negative regulatory factor PREG	1.17	6.56E-05	Cyclin-u4-1	GO:0005515; GO:0016538
Solyc02g078400.2	Allantoinase (AHRD V1 **** Q6S4R9_ROBPS); contains domain(s) IPR017593 Allantoinase	1.17	4.83E-07	Allantoinase	GO:0016810
Solyc08g075240.2	Glucose-methanol-choline oxidoreductase (AHRD V1 ***- D6TF66_9CHLR); Interpro domain(s) IPR012400 Long-chain fatty alcohol dehydrogenase	1.17	4.75E-05	Long-chain-alcohol oxidase fao4a	GO:0009055; GO:0046577; GO:0050660
Solyc12g087940.1	Aspartic proteinase nepenthesin-1 (AHRD V1 **-- contains Interpro domain(s) IPR001461 Peptidase A1	1.17	3.50E-05	Aspartic proteinase nepenthesin-1	GO:0004190; GO:0006508
Solyc01g091230.2	Receptor like kinase, RLK	1.16	5.17E-05	Lrr receptor-like serine threonine-protein kinase gso1	GO:0005515; GO:0004675
Solyc03g115870.2	Thioredoxin 2 (AHRD V1 ***- Q5ZF47_PLAMJ); Interpro domain(s) IPR015467 Thioredoxin, core	1.16	1.23E-03	Thioredoxin- chloroplastic	GO:0045454
Solyc04g074410.1	Os06g0220000 protein (Fragment) (AHRD V1 ***- contains Interpro domain(s) IPR006766 Phosphate-induced protein 1 conserved region	1.16	3.16E-04	Protein exordium	
Solyc06g065970.1	Cortical cell-delineating protein (AHRD V1 **-- contains Interpro domain(s) IPR013770 Plant lipid transfer protein and hydrophobic protein, helical	1.16	1.83E-04	14 kda proline-rich	GO:0006869
Solyc08g081790.1	Dirigent protein (AHRD V1 ***- Q9SDR7_FORIN); Interpro domain(s) IPR004265 Plant disease resistance response protein	1.16	2.71E-03	Disease resistance response protein 206	
Solyc01g068140.2	10-deacetylbaccatin III-10-O-acetyl transferase-like (AHRD V1 **-* contains Interpro domain(s) IPR003480 Transferase	1.15	1.19E-04	3 -n-debenzoyl-2 -deoxytaxol n-benzoyltransferase	GO:0016747

Solyc03g093610.1	Ethylene responsive transcription factor 1b (AHRD **** C0J9I8_9ROSA); contains Interpro domain(s) IPR001471 Pathogenesis-related transcriptional factor and ERF, DNA-binding	1.15	4.39E-04	Ethylene-responsive transcription factor 1	GO:0003700; GO:0006355
Solyc03g118370.2	Serine carboxypeptidase K10B2.2 (AHRD V1 **** contains Interpro domain(s) IPR001563 Peptidase S10, serine carboxypeptidase	1.15	6.21E-08	Serine carboxypeptidase	GO:0004185; GO:0006508
Solyc04g013200.1	Unknown Protein (AHRD V1)	1.15	2.45E-04	Unknown Protein	
Solyc06g007170.2	Os06g0207500 protein (Fragment) (AHRD V1 **-- contains Interpro domain(s) IPR004253 Protein of unknown function DUF231, plant	1.15	1.37E-04	Protein pmr5	
Solyc07g064990.2	S-adenosyl-L-methionine salicylic acid carboxyl methyltransferase-like protein V1 **** Q8LAR1_ARATH); contains Interpro domain(s) IPR005299 SAM dependent carboxyl methyltransferase	1.15	6.48E-05	Indole-3-acetate o-methyltransferase 1	GO:0008168
Solyc08g078940.1	Cortical cell-delineating protein (AHRD V1 **-- contains Interpro domain(s) IPR013770 Plant lipid transfer protein and hydrophobic protein, helical	1.15	4.85E-07	14 kda proline-rich	GO:0006869
Solyc11g011000.1	Cysteine-rich repeat secretory protein 60 (AHRD ***- CRR60_ARATH); contains Interpro domain(s) IPR002902 Protein of unknown function DUF26	1.15	4.92E-04	Cysteine-rich repeat secretory protein 60	
Solyc01g088400.2	CER1 (AHRD V1 **-- B6TFH3_MAIZE); contains domain(s) IPR006694 Fatty acid hydroxylase	1.14	1.18E-03	Protein eceriferum 1	GO:0005506; GO:0005783; GO:0006633; GO:0016491; GO:0055114
Solyc06g008770.1	Cc-nbs-lrr, resistance protein	1.14	2.45E-03	Nbs-lrr resistance protein	GO:0006952
Solyc07g018290.2	AP2-like ethylene-responsive transcription factor At1g16060 (AHRD *-*- AP2L1_ARATH); contains Interpro domain(s) IPR001471 Pathogenesis-related transcriptional factor and ERF, DNA-binding	1.14	4.19E-05	Ap2-like ethylene-responsive transcription factor ail5	GO:0003700; GO:0006355
Solyc10g055730.1	N-hydroxycinnamoyl/benzoyltransferase 4 (AHRD V1 ***- Q00M86_SOYBN); Interpro domain(s) IPR003480 Transferase	1.14	1.00E-03	Uncharacterized acetyltransferase	GO:0016747
Solyc02g071870.2	Receptor like kinase, RLK	1.13	8.14E-04	Probable lrr receptor-like serine threonine-protein kinase rfk1	GO:0004702; GO:0005515
Solyc03g118770.2	WUSCHEL-related homeobox-containing protein 4 (AHRD V1 C0LAL8_9MAGN); contains Interpro domain(s) IPR001356 Homeobox	1.13	1.23E-03	Wuschel-related homeobox 1	GO:0006355
Solyc05g053550.2	Chalcone synthase (AHRD V1 ***- C5IWS6_NICAL); Interpro domain(s) IPR011141 Polyketide synthase, type III	1.13	1.22E-05	Chalcone synthase	GO:0008415
Solyc06g084080.2	Guanylate-binding family protein (AHRD V1 ***- contains Interpro domain(s) IPR015900 Guanylate-binding protein-like, C-terminal	1.13	6.01E-04	Guanylate-binding protein 4 isoform	GO:0003924; GO:0005525
Solyc11g005710.1	WD-40 repeat family protein (AHRD V1 D7MSZ0_ARALY); contains Interpro domain(s) IPR020472 G-protein beta WD-40 repeat, region	1.13	1.38E-04	Wd repeat-containing protein 44	
Solyc02g072490.2	Os01g0841200 protein (Fragment) (AHRD V1 *--- contains Interpro domain(s) IPR004348 Protein of unknown function DUF246, plant	1.12	1.85E-03	O-fucosyltransferase family protein isoform 1	
Solyc09g061930.2	Receptor like kinase, RLK	1.12	4.05E-05	Kinase-like protein tmkl1	GO:0005515; GO:0004674
Solyc10g005400.2	Inositol oxygenase (AHRD V1 **** B9XK04_9BACT); Interpro domain(s) IPR007828 Protein of unknown function DUF706	1.12	8.97E-04	Inositol oxygenase 1	GO:0005506; GO:0005737; GO:0019310; GO:0050113; GO:0055114
Solyc03g115200.2	Glucan endo-1 3-beta-glucosidase 1 (AHRD V1 B6TW10_MAIZE); contains Interpro domain(s) IPR012946 X8	1.11	5.93E-04	Plasmodesmata callose-binding protein 3	
Solyc03g117560.2	Blue copper-like protein (AHRD V1 **-- contains Interpro domain(s) IPR003245 Plastocyanin-like	1.11	8.39E-05	Lamin-like protein	GO:0009055; GO:0005515
Solyc06g005980.2	Anthranilate synthase component I-1 (AHRD V1 B4F8P4_MAIZE); contains Interpro domain(s) IPR019999 Anthranilate synthase component I, C-terminal	1.11	1.67E-03	Anthranilate synthase alpha subunit chloroplastic-like	GO:0009058
Solyc06g060360.2	Universal stress protein family protein (AHRD ***- A8IXG1_BRACM); contains Interpro domain(s) IPR006016 UspA	1.11	2.28E-04	Adenine nucleotide alpha hydrolases-like superfamily protein	GO:0006950
Solyc06g073560.2	Isovaleryl-CoA dehydrogenase (AHRD V1 **** Q0MX57_BETVU); Interpro domain(s) IPR009100 Acyl-CoA dehydrogenase/oxidase, middle and N-terminal	1.11	1.04E-03	Isovaleryl- mitochondrial	GO:0003995
Solyc11g008830.1	LOB domain protein (AHRD V1 *-*- contains Interpro domain(s) IPR004883 Lateral organ boundaries, LOB	1.11	6.15E-04	Lob domain-containing protein 6	
Solyc02g079370.2	Cyclin-D6-1 (AHRD V1 **** CCD61_ORYSJ); contains domain(s) IPR015451 Cyclin D	1.1	1.15E-04	Cyclin-d6-1	n/a
Solyc04g082270.2	CM0216.210.nc protein (AHRD V1 ***- B0BLH4_LOTJA); Interpro domain(s) IPR004320 Protein of unknown function DUF241, plant	1.1	8.61E-04	Unknown Protein	

Solyc09g065660.2	Heat stress transcription factor A3 (AHRD ***- D1M7W9_SOLLC); contains Interpro domain(s) IPR000232 Heat shock factor (HSF)-type, DNA-binding	1.1	1.46E-04	Heat stress transcription factor a-7a	GO:0003700; GO:0005634; GO:0006355; GO:0043565
Solyc12g015690.1	Fasciclin-like arabinogalactan protein 10 (AHRD V1 A9XTL5_GOSHI); contains Interpro domain(s) IPR000782 FAS1 domain	1.1	7.66E-05	Fasciclin-like arabinogalactan protein 1	
Solyc01g006320.2	Non-race specific disease resistance protein 1-like b (AHRD V1 ***- D2CFI3_COFAR); contains Interpro domain(s) IPR010847 Harpin-induced 1	1.09	1.49E-04	Protein ndr1	GO:0005515; GO:0004871
Solyc01g099650.2	Formin 3 (AHRD V1 *-*- D0QAN4_ARATH); Interpro domain(s) IPR015425 Actin-binding FH2	1.09	2.10E-04	Formin-like protein 11	GO:0030036
Solyc02g094190.2	Nodulin family protein (AHRD V1 ***- contains Interpro domain(s) IPR010658 Nodulin-like	1.09	7.95E-04	Probable transporter mch1	
Solyc04g077490.2	AP2-like ethylene-responsive transcription factor At1g16060 (AHRD *-*- AP2L1_ARATH); contains Interpro domain(s) IPR001471 Pathogenesis-related transcriptional factor and ERF, DNA-binding	1.09	3.79E-05	Ap2-like ethylene-responsive transcription factor ant	GO:0003700; GO:0006355
Solyc07g018070.2	Heat shock protein-related (Fragment) (AHRD V1 B7ZEI4_ARATH); contains Interpro domain(s) IPR004176 Clp, N-terminal	1.09	1.78E-05	Heat shock protein	GO:0005515; GO:0019538
Solyc07g049370.2	Glucan endo-1 3-beta-glucosidase A6 (AHRD V1 B6TIF7_MAIZE); contains Interpro domain(s) IPR000490 Glycoside hydrolase, family 17	1.09	3.85E-04	Glucan endo- -beta-glucosidase 12	GO:0003824; GO:0005975; GO:0043169
Solyc11g011300.1	Rhamnogalacturonate lyase (AHRD V1 ***- A3XLZ2_LEEBM); Interpro domain(s) IPR010325 Rhamnogalacturonate lyase	1.09	1.94E-03	Probable rhamnogalacturonate lyase b	GO:0030246
Solyc03g123590.2	Remorin family protein (AHRD V1 ***- contains Interpro domain(s) IPR005516 Remorin, C-terminal region	1.08	9.40E-04	Dna binding	GO:0003677
Solyc04g080490.2	Zinc finger-homeodomain protein 1 (Fragment) (AHRD *--- B0LK17_YUCFI); contains Interpro domain(s) IPR006456 ZF-HD homeobox protein Cys/His-rich dimerisation region	1.08	4.79E-06	Zinc-finger homeodomain protein 5	GO:0003677; GO:0045449
Solyc06g068550.2	Aspartic proteinase nepenthesin-1 (AHRD V1 **-- contains Interpro domain(s) IPR001461 Peptidase A1	1.08	3.38E-05	Protein aspartic protease in guard cell 2	GO:0004190; GO:0006508
Solyc06g076220.2	Expansin-1 (AHRD V1 ***- Q6RX69_PETHY); contains domain(s) IPR002963 Expansin	1.08	1.47E-05	Expansin-a6	GO:0009664
Solyc07g008010.2	Myb transcription factor (AHRD V1 *--* contains Interpro domain(s) IPR015495 Myb transcription factor	1.08	7.71E-04	Transcription factor myb82	GO:0003677; GO:0045449
Solyc07g055950.2	Meiosis 5 (AHRD V1 *-*- B6T5X0_MAIZE)	1.08	4.18E-04	Protodermal factor 1	
Solyc01g079580.2	DNAJ heat shock N-terminal domain-containing protein V1 ***- D7LGN0_ARALY); contains Interpro domain(s) IPR011990 Tetratricopeptide-like helical	1.07	1.07E-04	Heat shock protein with tetratricopeptide repeat isoform 1	GO:0031072
Solyc01g107370.2	Gibberellin-regulated family protein (AHRD V1 **-- contains Interpro domain(s) IPR003854 Gibberellin regulated protein	1.07	2.61E-04	Gibberellin-regulated protein	
Solyc03g026040.2	Receptor like kinase, RLK	1.07	3.45E-04	Leucine-rich repeat receptor protein kinase exs	GO:0004675; GO:0006468
Solyc03g119990.2	Hydrolase alpha/beta fold family protein (AHRD **** D7KX74_ARALY); contains Interpro domain(s) IPR000073 Alpha/beta hydrolase fold-1	1.07	3.81E-04	Unknown Protein	GO:0016787
Solyc04g078770.2	Heat stress transcription factor (AHRD V1 D4QAU8_CARPA); contains Interpro domain(s) IPR000232 Heat shock factor (HSF)-type, DNA-binding	1.07	7.96E-04	Heat stress transcription factor b-4	GO:0003700; GO:0005634; GO:0006355; GO:0043565
Solyc06g068880.2	Serine carboxypeptidase 1 (AHRD V1 **** contains Interpro domain(s) IPR001563 Peptidase S10, serine carboxypeptidase	1.07	8.76E-06	Unknown Protein	GO:0004185; GO:0006508
Solyc10g045290.1	Kinase interacting family protein (AHRD V1 D7KCB2_ARALY); contains Interpro domain(s) IPR011684 KIP1-like	1.07	7.42E-04	Intracellular protein transport protein uso1	
Solyc01g088380.1	DNA helicase (AHRD V1 ***- A6ZT72_YEAS7); Interpro domain(s) IPR014808 DNA replication factor Dna2	1.06	3.62E-05	Dna replication atp-dependent helicase nuclease dna2 isoform	GO:0003677; GO:0004003; GO:0005524; GO:0006260
Solyc01g100750.2	Susceptibility homeodomain transcription factor (Fragment) (AHRD *-*- Q8SAA7_ORYSA); contains Interpro domain(s) IPR007493 Protein of unknown function DUF538	1.06	8.90E-04	Unknown Protein	GO:0005515
Solyc02g090480.2	Peptidyl-prolyl cis-trans isomerase D (AHRD V1 PPID_RAT); contains Interpro domain(s) IPR002130 Peptidyl-prolyl cis-trans isomerase, cyclophilin-type	1.06	7.23E-04	Peptidyl-prolyl cis-trans isomerase cyp40	GO:0003755; GO:0006457
Solyc03g006840.2	Genomic DNA chromosome 5 P1 clone (AHRD V1 ***- Q9FIS0_ARATH)	1.06	3.99E-05	Protein longifolia 1	
Solyc03g121170.2	GDSL esterase/lipase At5g22810 (AHRD V1 ***- contains Interpro domain(s) IPR001087 Lipase, GDSL	1.06	1.08E-04	Gdsl esterase lipase apg	GO:0006629; GO:0004091

Solyc08g063090.2	Delta-6-desaturase (AHRD V1 **** D3YN48_9ERIC); contains domain(s) IPR012171 Fatty acid/sphingolipid desaturase	1.06	6.70E-04	Delta -fatty-acid desaturase 2	GO:0020037
Solyc09g061280.2	Cyclin dependent kinase inhibitor (AHRD V1 Q5PXG5_EUPES); contains Interpro domain(s) IPR016701 Cyclin-dependent kinase inhibitor, plant	1.06	2.40E-03	Cyclin-dependent kinase inhibitor 3	GO:0005634; GO:0030332
Solyc09g065240.2	Patatin-like protein 3 (AHRD V1 ***- contains Interpro domain(s) IPR002641 Patatin	1.06	1.28E-03	Probable inactive patatin-like protein 9	GO:0006629
Solyc02g089130.2	COBRA-like protein (AHRD V1 ***- B6E8Y8_9MYRT); Interpro domain(s) IPR017391 COBRA-like	1.05	1.96E-04	Cobra-like protein 4	
Solyc08g005630.2	Glucose-methanol-choline oxidoreductase (AHRD V1 ***- D6TF66_9CHLR); Interpro domain(s) IPR012400 Long-chain fatty alcohol dehydrogenase	1.05	3.50E-06	Long-chain-alcohol oxidase fao4a	GO:0009055; GO:0046577; GO:0050660
Solyc08g075680.2	Unknown Protein (AHRD V1)	1.05	5.44E-04	Coiled-coil domain-containing protein 136 isoform	
Solyc01g007800.2	Plant-specific domain TIGR01568 family protein (AHRD *--- B6SZV1_MAIZE); contains Interpro domain(s) IPR006458 Protein of unknown function DUF623, plant	1.04	4.90E-05	Transcription repressor ofp6	
Solyc01g096070.2	Auxin response factor 9 (AHRD V1 D7R605_SOLLC); contains Interpro domain(s) IPR010525 Auxin response factor	1.04	2.55E-05	Auxin response factor 18	GO:0003677; GO:0005634; GO:0009725; GO:0045449
Solyc01g096450.2	Aspartic proteinase nepenthesin-1 (AHRD V1 **-- contains Interpro domain(s) IPR001461 Peptidase A1	1.04	1.71E-03	Protein aspartic protease in guard cell 2-	GO:0031177
Solyc06g066160.2	Pinoresinol-lariciresinol reductase (AHRD V1 **** Q4R0I0_9ROSI); Interpro domain(s) IPR008030 NmrA-like	1.04	9.24E-04	Bifunctional pinoresinol-lariciresinol reductase 2	GO:0006808; GO:0016564
Solyc09g030450.2	Receptor like kinase, RLK	1.04	1.91E-04	Probable inactive receptor kinase	GO:0004672; GO:0005524; GO:0006468
Solyc10g018780.1	Squamosa promoter binding protein 1 (AHRD *-*- Q1MU24_PHYPA); contains Interpro domain(s) IPR004333 Transcription factor, SBP-box	1.04	2.45E-03	Squamosa promoter-binding-like protein 8	GO:0003677; GO:0005634
Solyc10g084380.1	WRKY transcription factor 42 (Fragment) (AHRD **-- B2KJ82_HORVD); contains Interpro domain(s) IPR003657 DNA-binding WRKY	1.04	1.19E-05	Wrky transcription factor 44	GO:0003700; GO:0005634; GO:0043565; GO:0045449
Solyc01g091540.1	Growth-regulating factor 12 (AHRD V1 *--- contains Interpro domain(s) IPR014977 WRC	1.03	7.00E-04	Growth-regulating factor 9 isoform	GO:0005634
Solyc03g005960.2	Receptor like kinase, RLK	1.03	3.51E-04	Probable lrr receptor-like serine threonine-protein kinase	GO:0004702; GO:0005515; GO:0006468
Solyc03g031420.1	Molybdenum cofactor sulfurase (AHRD V1 ***- contains Interpro domain(s) IPR015424 Pyridoxal phosphate-dependent transferase, major region	1.03	1.12E-03	Molybdenum cofactor sulfurase	GO:0003824; GO:0030170
Solyc05g005320.1	Unknown Protein (AHRD V1)	1.03	2.19E-04	Unknown Protein	
Solyc05g026480.1	WD-40 repeat family protein (AHRD V1 D7LF16_ARALY); contains Interpro domain(s) IPR017986 WD40 repeat, region	1.03	2.20E-03	Transcriptional corepressor leunig-like isoform	
Solyc10g078680.1	Phosphoribosylanthranilate transferase (Fragment) (AHRD V1 *--- contains Interpro domain(s) IPR013583 Phosphoribosyltransferase C-terminal, plant	1.03	4.03E-04	Multiple c2 and transmembrane domain-containing protein 1-like	
Solyc02g080260.2	Homeobox leucine-zipper protein (AHRD V1 ***- contains Interpro domain(s) IPR002913 Lipid-binding START	1.02	1.36E-06	Homeobox-leucine zipper protein meristem l1-like	GO:0003677; GO:0045449
Solyc02g080390.2	Abnormal spindle-like microcephaly-associated protein (Fragment) (AHRD *-*- B6UL97_HYLME); contains Interpro domain(s) IPR016146 Calponin-homology	1.02	1.92E-04	Abnormal spindle- microcephaly-associated protein homolog	
Solyc02g094520.2	Histone-lysine N-methyltransferase (AHRD V1 *-*- B6K768_SCHJY); Interpro domain(s) IPR003105 SRA-YDG	1.02	2.15E-04	Histone-lysine n- h3 lysine-9 specific suvh4	GO:0005634; GO:0008270; GO:0016568; GO:0018024
Solyc04g026110.2	Cc-nbs, resistance protein fragment	1.02	6.06E-06	R2 late blight resistance protein	GO:0006952
Solyc04g080270.2	Genomic DNA chromosome 5 P1 clone (AHRD V1 *-*- Q9FMZ3_ARATH)	1.02	8.82E-04	Dentin sialophospho	
Solyc06g007610.2	Early tobacco anther 1 (AHRD V1 Q84XA0_TOBAC)	1.02	2.04E-05	Lactoylglutathione lyase glyoxalase i family protein	GO:0003824
Solyc08g068670.2	Decarboxylase family protein (AHRD V1 ***- contains Interpro domain(s) IPR002129 Pyridoxal phosphate-dependent decarboxylase	1.02	1.98E-04	Histidine decarboxylase	GO:0003824; GO:0030170
Solyc08g077990.2	Kinase like protein (AHRD V1 *-*- contains Interpro domain(s) IPR002290 Serine/threonine protein kinase	1.02	1.88E-05	Probable serine threonine-protein kinase cdc7	GO:0004674; GO:0006468
Solyc09g007330.2	DNA replication licensing factor MCM6 (AHRD **** B3LHP9_YEAS1); contains Interpro domain(s) IPR001208 DNA-dependent ATPase MCM	1.02	2.09E-04	Probable dna helicase mcm8 isoform	GO:0043140; GO:0006260
Solyc09g090680.2	Cysteine-rich repeat secretory protein 3 (AHRD ***- CRR3_ARATH); contains Interpro domain(s) IPR002902 Protein of unknown function DUF26	1.02	2.60E-03	Cysteine-rich repeat secretory protein 3	GO:0030001; GO:0046872

Solyc11g072840.1	Histone H4 (AHRD V1 ***- B6T0P4_MAIZE); Interpro domain(s) IPR001951 Histone H4	1.02	1.17E-04	Histone h4	GO:0000786; GO:0003677; GO:0005634; GO:0006334
Solyc12g007160.1	Protein ABIL1 (AHRD V1 *--- B6TCZ2_MAIZE)	1.02	1.81E-03	Probable protein abil5	
Solyc05g009100.2	Receptor like kinase, RLK	1.01	4.23E-05	Probable leucine-rich repeat receptor-like protein kinase	GO:0004672; GO:0005524; GO:0006468
Solyc07g054170.2	Expansin B1 (AHRD V1 ***- C8CC40_RAPSA); Interpro domain(s) IPR007117 Pollen allergen/expansin, C-terminal	1.01	1.38E-03	Expansin-b3	GO:0005576
Solyc11g006250.1	GDSL esterase/lipase At5g33370 (AHRD V1 ***- contains Interpro domain(s) IPR001087 Lipase, GDSL	1.01	1.25E-03	Gdsl esterase lipase	GO:0006629; GO:0016788
Solyc11g068890.1	NHL1 (Fragment) (AHRD V1 **-- B0LZK8_ARATH); Interpro domain(s) IPR010847 Harpin-induced 1	1.01	4.81E-04	Protein yls9	
Solyc12g005020.1	Ring H2 finger protein (AHRD V1 D9ZHD8_HYPPE); contains Interpro domain(s) IPR018957 Zinc finger, C3HC4 RING-type	1.01	1.72E-04	Nep1-interacting 1	GO:0005141; GO:0008270
Solyc12g089050.1	Wax synthase isoform 1 (AHRD V1 Q84XY9_VITVI)	1.01	1.03E-03	Acyl- --sterol o-acyltransferase 1	GO:0005515; GO:0008415
Solyc02g092420.2	Os07g0587200 protein (Fragment) (AHRD V1 *--- contains Interpro domain(s) IPR013069 BTB/POZ	1	1.01E-03	Btb poz domain-containing protein	GO:0005515
Solyc03g083510.2	LRR receptor-like serine/threonine-protein kinase, RLP	1	6.66E-05	Probable lrr receptor-like serine threonine-protein kinase	GO:0005515; GO:0004675
Solyc03g097170.2	Cinnamoyl-CoA reductase-like protein (AHRD V1 ***- contains Interpro domain(s) IPR016040 NAD(P)-binding domain	1	4.68E-04	Cinnamoyl- reductase 1	GO:0003824; GO:0005488; GO:0008152
Solyc04g077510.2	Growth regulating factor 1 (Fragment) (AHRD *--- A6MZS4_ORYSI); contains Interpro domain(s) IPR014977 WRC	1	5.25E-04	Growth-regulating factor 1	GO:0005634; GO:0016563; GO:0005515
Solyc05g015840.2	Squamosa promoter-binding protein (AHRD V1 ***- contains Interpro domain(s) IPR004333 Transcription factor, SBP-box	1	4.68E-05	Squamosa promoter-binding-like protein 16	GO:0003677; GO:0005634
Solyc06g072660.2	Dek protein (AHRD V1 *-*- B6SWA1_MAIZE); Interpro domain(s) IPR014876 DEK, C-terminal	1	1.10E-03	Protein dek-like isoform	
Solyc10g008320.1	Unknown Protein (AHRD V1)	1	4.39E-05	Unknown Protein	
Solyc02g070540.2	Os01g0611000 protein (Fragment) (AHRD V1 ***- contains Interpro domain(s) IPR006946 Protein of unknown function DUF642	0.99	8.11E-05	Unknown protein	
Solyc02g072140.1	UNE1-like protein (AHRD V1 ***- B2ZAQ7_GOSRA); Interpro domain(s) IPR006943 Protein of unknown function DUF641, plant	0.99	6.06E-05	Une1-like protein	
Solyc04g076400.2	Vernalization insensitive 3 (Fragment) (AHRD V1 C4PFF9_BRARC)	0.99	1.30E-03	Vin3-like protein 2	GO:0005515
Solyc04g078470.2	Cyclin D3-1 (AHRD V1 ***- Q2ABE7_CAMSI); Interpro domain(s) IPR015451 Cyclin D	0.99	2.21E-04	Cyclin-d3-3-like isoform x2	GO:0005634; GO:0004723
Solyc06g059930.2	Sesquiterpene synthase 1 (AHRD V1 ***-	0.99	1.97E-04	Germacrene c synthase-	n/a
Solyc07g053450.2	BZIP transcription factor family protein (AHRD **** D7LW03_ARALY); contains Interpro domain(s) IPR011700 Basic leucine zipper	0.99	1.32E-03	Basic leucine zipper 61	GO:0003700; GO:0005634; GO:0006355; GO:0043565; GO:0046983
Solyc08g082990.2	Oligopeptide transporter 9 (AHRD V1 **** contains Interpro domain(s) IPR004648 Tetrapeptide transporter, OPT1/isp4	0.99	1.13E-06	Oligopeptide transporter 7	GO:0055085; GO:0005427
Solyc09g018020.2	Expansin (AHRD V1 ***- A5H0F8_CARCN); contains domain(s) IPR002963 Expansin	0.99	2.54E-03	Expansin alpha	GO:0009664
Solyc04g008600.2	Chromosome segregation in meiosis protein 3 V1 *--- C0NYU8_AJECG); contains Interpro domain(s) IPR012923 Replication fork protection component Swi3	0.98	6.45E-04	Timeless-interacting protein	GO:0003676; GO:0008270
Solyc06g072240.1	Histone H4 (AHRD V1 ***- B6T0P4_MAIZE); Interpro domain(s) IPR001951 Histone H4	0.98	1.53E-03	Histone h4	GO:0000786; GO:0003677; GO:0005634; GO:0006334
Solyc09g011000.2	Laccase (AHRD V1 ***- O24042_LIRTU); contains domain(s) IPR011707 Multicopper oxidase, type 3	0.98	6.54E-04	Laccase-17	GO:0016491
Solyc11g006650.1	Heat shock protein 101 (AHRD V1 Q3L1D0_VITVI); contains Interpro domain(s) IPR004176 Clp, N-terminal	0.98	3.39E-04	Double clp-n motif-containing p-loop nucleoside triphosphate hydrolases superfamily	GO:0005515; GO:0019538
Solyc02g085390.2	Uncharacterized ATP-dependent helicase C25A8.01c (AHRD V1 YFC1_SCHPO); contains Interpro domain(s) IPR000330 SNF2-related	0.97	3.54E-04	Atp-dependent dna helicase ddm1	GO:0003677; GO:0005524

Solyc03g122140.2	L-lactate dehydrogenase (AHRD V1 ***- B2WEY8_PYRTR); Interpro domain(s) IPR012133 Alpha-hydroxy acid dehydrogenase, FMN-dependent	0.97	9.62E-04	Peroxisomal -2-hydroxy-acid oxidase glo4-	GO:0010181; GO:0016491
Solyc06g051320.2	Transferase family protein (AHRD V1 **-* contains Interpro domain(s) IPR003480 Transferase	0.97	1.68E-04	Vinorine synthase	GO:0016747
Solyc07g048100.1	BRCA1 C Terminus domain containing protein (AHRD V1 *-*- Q6ATS1_ORYSJ); contains Interpro domain(s) IPR001357 BRCT	0.97	1.72E-03	Unknown protein	GO:0005622
Solyc07g066350.1	Unknown Protein (AHRD V1)	0.97	1.86E-03	Uncharacterized serine-rich protein	
Solyc09g042710.2	Myosin heavy chain-like (AHRD V1 ***- contains Interpro domain(s) IPR008545 Protein of unknown function DUF827, plant	0.97	2.49E-03	Web family protein	
Solyc09g061890.2	Pectate lyase 46388 (AHRD V1 **** contains Interpro domain(s) IPR002022 Pectate lyase/Amb allergen	0.97	7.07E-04	Probable pectate lyase 8	GO:0016829
Solyc10g047530.1	Phototropic-responsive NPH3 family protein (AHRD V1 D7KEU3_ARALY); contains Interpro domain(s) IPR004249 NPH3	0.97	2.40E-03	Root phototropism protein 3	GO:0005515
Solyc00g099580.1	Gamma-glutamyltranspeptidase (AHRD V1 ***- D4Z6K6_SPHJU); contains domain(s) IPR000101 Gamma-glutamyltranspeptidase	0.96	2.81E-03	Unknown Protein	GO:0003677; GO:0045449
Solyc01g102310.2	Unknown Protein (AHRD V1)	0.96	9.61E-06	Grip and coiled-coil domain-containing	
Solyc01g111840.2	MFS-type drug efflux transporter P55 (AHRD *--* MFS55_MYCTU); contains Interpro domain(s) IPR016196 Major facilitator superfamily, general substrate transporter	0.96	5.70E-04	Hippocampus abundant transcript-like protein 1 isoform x2	GO:0055085; GO:0008493
Solyc02g079490.2	Hydroxycinnamoyl-CoA shikimate/quinate hydroxycinnamoyl transferase (AHRD V1 D1GJ94_COFAR); contains Interpro domain(s) IPR003480 Transferase	0.96	3.33E-04	Shikimate o-hydroxycinnamoyltransferase	GO:0016747
Solyc02g086840.2	Kinesin light chain-like protein (AHRD V1 D2DWB2_PHAVU); contains Interpro domain(s) IPR011990 Tetratricopeptide-like helical	0.96	1.33E-04	Tetratricopeptide repeat protein 28	GO:0005488
Solyc02g087980.2	Structural maintenance of chromosomes protein 4 V1 **-- D8TXU8_VOLCA); contains Interpro domain(s) IPR003395 RecF/RecN/SMC protein, N-terminal	0.96	7.31E-04	Structural maintenance of chromosomes protein 4	GO:0005524; GO:0005694
Solyc03g120930.1	Avr9/Cf-9 rapidly elicited protein 146 (AHRD ***- Q9FQZ6_TOBAC)	0.96	1.50E-03	Avr9/Cf-9 rapidly elicited protein 146	
Solyc04g015620.2	Os01g0611000 protein (Fragment) (AHRD V1 ***- contains Interpro domain(s) IPR006946 Protein of unknown function DUF642	0.96	1.86E-04	Unknown protein	
Solyc09g011820.2	Unknown Protein (AHRD V1)	0.96	2.83E-03	Gpi-anchored protein	
Solyc12g044630.1	Profilin (AHRD V1 ***- Q9SMC0_TOBAC); contains domain(s) IPR005455 Profilin, plant	0.96	7.11E-04	Profilin	GO:0003779; GO:0030036
Solyc02g083610.2	BZIP transcription factor (AHRD V1 *-*- contains Interpro domain(s) IPR006867 Protein of unknown function DUF632	0.95	1.06E-03	Unknown protein	GO:0003677
Solyc02g089550.2	Receptor like kinase, RLK	0.95	2.35E-05	Protein nsp-interacting kinase 1	GO:0004672; GO:0005524; GO:0006468
Solyc06g076920.2	Undecaprenyl pyrophosphate synthase (AHRD V1 **** contains Interpro domain(s) IPR001441 Di-trans-poly-cis-decaprenylcistransferase-like	0.95	2.82E-03	Dehydrodolichyl diphosphate synthase 2	GO:0016765
Solyc08g080280.2	Unknown Protein (AHRD V1)	0.95	8.26E-04	Unknown Protein	
Solyc12g011010.1	Meiosis 5 (AHRD V1 *-*- B6T5X0_MAIZE)	0.95	1.30E-03	Protodermal factor 1	
Solyc03g117550.1	Receptor protein kinase-like (AHRD V1 *-*- contains Interpro domain(s) IPR002290 Serine/threonine protein kinase	0.94	2.73E-04	Receptor-like protein kinase at1g80870	GO:0004553; GO:0005975
Solyc08g042100.2	Armadillo/beta-catenin repeat family protein (AHRD V1 D7MAU3_ARALY); contains Interpro domain(s) IPR011989 Armadillo-like helical	0.94	4.63E-05	Arm repeat superfamily protein isoform 1	GO:0005488
Solyc08g080730.2	Senescence-associated protein (AHRD V1 ***- B3TLS7_ELAGV); Interpro domain(s) IPR000301 Tetraspanin, subgroup	0.94	2.53E-03	Tetraspanin-10	GO:0016021
Solyc03g083720.1	Pectinesterase (AHRD V1 ***- C0PST8_PICSI); contains domain(s) IPR006501 Pectinesterase inhibitor	0.93	2.55E-03	Pectinesterase	GO:0004857; GO:0030599
Solyc04g075000.1	Serine/threonine protein kinase (AHRD V1 *-** contains Interpro domain(s) IPR002290 Serine/threonine protein kinase	0.93	1.35E-03	Serine threonine-protein kinase-like protein	GO:0004672; GO:0005524; GO:0006468
Solyc02g062780.2	Chromodomain-helicase-DNA-binding protein 6 (AHRD V1 **-* contains Interpro domain(s) IPR000330 SNF2-related	0.92	2.01E-05	Atp-dependent dna helicase ddm1-like isoform x1	GO:0003677; GO:0005524

Solyc03g093250.1	Structural maintenance of chromosomes 2 (AHRD **-- Q52KE9_MOUSE); contains Interpro domain(s) IPR003395 RecF/RecN/SMC protein, N-terminal	0.92	1.01E-03	Structural maintenance of chromosomes protein	GO:0005524; GO:0005694
Solyc03g118740.2	Auxin efflux carrier (AHRD V1 ***- contains Interpro domain(s) IPR014024 Auxin efflux carrier, subgroup	0.92	1.17E-05	Probable auxin efflux carrier component 1c	GO:0016021
Solyc07g052240.2	Laccase-22 (AHRD V1 **-- LAC22_ORYSJ); contains domain(s) IPR001117 Multicopper oxidase, type 1	0.92	2.25E-03	L-ascorbate oxidase homolog	GO:0016491
Solyc08g014190.2	Cytochrome P450	0.92	1.80E-03	Geraniol 8-hydroxylase	n/a
Solyc09g065590.2	Os12g0604200 protein (Fragment) (AHRD V1 *-*- contains Interpro domain(s) IPR008546 Protein of unknown function DUF828, plant	0.92	2.53E-03	Auxin canalization protein	GO:0035091
Solyc11g010850.1	1-deoxy-D-xylulose 5-phosphate synthase 2 (AHRD V1 C7U111_SOLLC); contains Interpro domain(s) IPR005477 Deoxyxylulose-5-phosphate synthase	0.92	3.97E-05	Probable 1-deoxy-d-xylulose-5-phosphate synthase chloroplastic	GO:0008661; GO:0016114
Solyc01g067510.2	Receptor-like kinase (AHRD V1 **** A7VM36_MARPO); Interpro domain(s) IPR002290 Serine/threonine protein kinase	0.91	1.05E-03	Proline-rich receptor-like protein kinase perk3	GO:0005515; GO:0004674; GO:0006468
Solyc01g095100.2	WRKY transcription factor 23 (AHRD V1 C9DI12_9ROSI); contains Interpro domain(s) IPR003657 DNA-binding WRKY	0.91	1.90E-03	Wrky transcription factor 22	GO:0003700; GO:0005634; GO:0043565; GO:0045449
Solyc01g100030.2	Deoxyuridine 5&apos-triphosphate nucleotidohydrolase (AHRD V1 ***- contains Interpro domain(s) IPR008181 DeoxyUTP pyrophosphatase subfamily 1	0.91	9.25E-04	Deoxyuridine 5 -triphosphate nucleotidohydrolase	GO:0016787; GO:0046080
Solyc07g061920.2	Glucan synthase like 3 (AHRD V1 D8R8I5_SELML); contains Interpro domain(s) IPR003440 Glycosyl transferase, family 48	0.91	2.98E-04	Callose synthase 8	GO:0000148; GO:0003843; GO:0006075; GO:0016020
Solyc08g083210.2	Endoglucanase 1 (AHRD V1 ***- B6U0P7_MAIZE); Interpro domain(s) IPR001701 Glycoside hydrolase, family 9	0.91	3.57E-04	Endo- -beta-glucanase	GO:0004553; GO:0005975
Solyc12g094700.1	Cathepsin B-like cysteine proteinase (AHRD V1 CYSP_SCHMA); contains Interpro domain(s) IPR013128 Peptidase C1A, papain	0.91	6.46E-05	Xylem cysteine proteinase 1-	GO:0004197; GO:0006508
Solyc01g080070.2	Copper chaperone (AHRD V1 *--- B6TJ24_MAIZE); Interpro domain(s) IPR006121 Heavy metal transport/detoxification protein	0.9	3.76E-05	Neurogenic protein mastermind	GO:0030001; GO:0046872
Solyc01g080770.2	Receptor like kinase, RLK	0.9	7.26E-04	Leucine-rich repeat receptor-serine threonine-protein kinase bam3	GO:0004674; GO:0006468
Solyc01g111330.2	Thaumatin-like protein (AHRD V1 ***- Q9STX6_ARATH); Interpro domain(s) IPR001938 Thaumatin, pathogenesis-related	0.9	7.72E-04	Thaumatin-like protein 1b	GO:0005515
Solyc02g093400.1	Integral membrane protein MviN (AHRD V1 D8G376_9CYAN); contains Interpro domain(s) IPR004268 Flagellin assembly, membrane protein MviN	0.9	2.64E-03	Lipid ii flippase	
Solyc03g114690.2	Wd-40 repeat-containing protein (AHRD V1 *--- contains Interpro domain(s) IPR017986 WD40 repeat, region	0.9	2.87E-04	Wd repeat and hmg-box dna-binding protein 1	GO:0000166
Solyc06g069790.2	Gibberellin-regulated protein (AHRD V1 **-- B4UW77_ARAHY); Interpro domain(s) IPR003854 Gibberellin regulated protein	0.9	1.37E-04	Gibberellin-regulated protein 6	
Solyc06g083490.2	Tropinone reductase-like protein 16 (AHRD V1 B2BXZ1_9BRAS); contains Interpro domain(s) IPR002347 Glucose/ribitol dehydrogenase	0.9	7.27E-04	Tropinone reductase homolog	GO:0008152; GO:0016491
Solyc07g008710.2	Major latex-like protein (AHRD V1 **-- contains Interpro domain(s) IPR000916 Bet v I allergen	0.9	1.52E-03	Mlp-like protein 34	GO:0006952; GO:0009607
Solyc07g041970.2	Subtilisin-like protease (AHRD V1 **-- B6SZ82_MAIZE); Interpro domain(s) IPR015500 Peptidase S8, subtilisin-related	0.9	4.74E-05	Subtilisin-like protease	GO:0004252; GO:0006508
Solyc09g009900.2	Pollen-specific protein - like (AHRD V1 O49527_ARATH); contains Interpro domain(s) IPR013970 Replication factor A protein 3	0.9	9.95E-04	Replication protein a 14 kda subunit b	GO:0005515
Solyc09g072820.2	Cellulose synthase (AHRD V1 **** B8XPP6_9ROSI); Interpro domain(s) IPR005150 Cellulose synthase	0.9	4.95E-06	Cellulose synthase a catalytic subunit 4	GO:0005515; GO:0008270
Solyc09g082830.2	ARGONAUTE 1 (AHRD V1 ***- D6RUV9_TOBAC); Interpro domain(s) IPR003165 Stem cell self-renewal protein Piwi	0.9	2.26E-05	Protein argonaute 10	GO:0003676
Solyc09g090500.2	Cyclopropane-fatty-acyl-phospholipid synthase (AHRD V1 ***- C7LRK7_DESBD); Interpro domain(s) IPR003333 Cyclopropane-fatty-acyl-phospholipid synthase	0.9	3.06E-04	Pavine n-methyltransferase	GO:0008610; GO:0008825
Solyc09g092520.2	Xyloglucan endotransglucosylase/hydrolase 8 (AHRD V1 **** contains Interpro domain(s) IPR016455 Xyloglucan endotransglucosylase/hydrolase	0.9	1.27E-03	Brassinosteroid-regulated protein bru1	GO:0004553; GO:0005975
Solyc12g015800.1	RING finger family protein (AHRD V1 D6RU92_SILLA); contains Interpro domain(s) IPR018957 Zinc finger, C3HC4 RING-type	0.9	1.37E-03	E3 ubiquitin-protein ligase at4g11680	GO:0008270
Solyc08g076370.2	Homeobox-leucine zipper protein ATHB-9 (AHRD V1 ATBH9_ARATH); contains Interpro domain(s) IPR002913 Lipid-binding START	0.89	4.41E-06	Homeobox-leucine zipper protein hdg5	GO:0003677; GO:0045449

Solyc08g077210.2	Inositol 1 4 5-trisphosphate 5-phosphatase (AHRD *-** Q712G2_ARATH); contains Interpro domain(s) IPR000300 Inositol polyphosphate related phosphatase	0.89	3.34E-04	Type i inositol -trisphosphate 5-phosphatase 2-like	GO:0004437; GO:0004445
Solyc10g080690.1	Patatin-like protein 3 (AHRD V1 ***- contains Interpro domain(s) IPR002641 Patatin	0.89	9.85E-04	Patatin-like protein 3	GO:0006629
Solyc10g084150.1	Cytokinin riboside 5'-monophosphate phosphoribohydrolase LOG (AHRD **-- LOG_ORYSJ); contains Interpro domain(s) IPR005269 Conserved hypothetical protein CHP00730	0.89	8.31E-05	Cytokinin riboside 5 -monophosphate phosphoribohydrolase log3	
Solyc01g101030.2	FACT complex subunit SPT16 (AHRD V1 D3AWW1_POLPA); contains Interpro domain(s) IPR013953 FACT complex subunit Spt16p/Cdc68p	0.88	1.92E-04	Fact complex subunit spt16	GO:0009987; GO:0042393
Solyc05g056470.1	ABC transporter G family member 5 V1 **** AB5G_ARATH); contains Interpro domain(s) IPR013525 ABC-2 type transporter	0.88	7.42E-06	Abc transporter g family member 5	GO:0016020
Solyc07g005840.2	Cellulose synthase 3 (AHRD V1 **** contains Interpro domain(s) IPR005150 Cellulose synthase	0.88	8.64E-06	Cellulose synthase a catalytic subunit 7	GO:0005515; GO:0008270
Solyc07g054900.2	Alpha-hydroxynitrile lyase (AHRD V1 **-- O49897_MANES); Interpro domain(s) IPR000073 Alpha/beta hydrolase fold-1	0.88	2.57E-03	Polyneuridine-aldehyde esterase	GO:0080030; GO:0080031
Solyc08g066810.2	Glycosyl hydrolase family 5 protein/cellulase (AHRD **-- A8NVS8_COPC7); contains Interpro domain(s) IPR001547 Glycoside hydrolase, family 5	0.88	6.20E-04	Cellulase protein	GO:0003824; GO:0005975; GO:0043169
Solyc09g014160.1	UPF0503 protein At3g09070, chloroplastic (AHRD V1 U503A_ARATH); contains Interpro domain(s) IPR008004 Protein of unknown function DUF740	0.88	6.36E-04	Upf0503 protein chloroplastic	
Solyc09g092760.2	Glycine-rich protein (AHRD V1 *-*- D2K2U4_TOBAC); Interpro domain(s) IPR010800 Glycine rich	0.88	1.24E-03	Glycine-rich cell wall structural	
Solyc12g100180.1	ATP-binding cassette transporter (AHRD V1 ***- contains Interpro domain(s) IPR013525 ABC-2 type transporter	0.88	6.05E-04	Pleiotropic drug resistance protein 1	GO:0016020
Solyc01g094750.2	Cytochrome P450	0.87	1.07E-04	Cytochrome p450 86a8-	GO:0004497; GO:0005506; GO:0020037
Solyc02g084950.2	Carboxyl methyltransferase 4 (AHRD V1 **** contains Interpro domain(s) IPR005299 SAM dependent carboxyl methyltransferase	0.87	5.03E-05	Salicylate o-methyltransferase	GO:0008168
Solyc03g031910.2	Helicase sen1 (AHRD V1 *--- A8NRR5_COPC7)	0.87	1.73E-03	P-loop containing nucleoside triphosphate hydrolases superfamily	
Solyc03g064010.2	Receptor like kinase, RLK	0.87	9.68E-05	Probable inactive leucine-rich repeat receptor-like protein kinase	GO:0004674; GO:0006468
Solyc03g083350.2	Ubiquitin (AHRD V1 **-- Q9FNF8_ARATH); contains domain(s) IPR000403 Phosphatidylinositol -3 and 4-kinase, catalytic	0.87	1.19E-03	Phosphatidylinositol 4-kinase gamma 3	GO:0016773; GO:0043008
Solyc03g115050.2	Single-stranded DNA-binding replication protein A large (AHRD V1 **** D2V0Z2_NAEGR); contains Interpro domain(s) IPR004591 Replication factor-a protein 1 Rpa1	0.87	2.88E-03	Replication protein a 70 kda dna-binding subunit b	GO:0003677; GO:0005634; GO:0006260
Solyc04g005700.2	Major latex-like protein (AHRD V1 **-- contains Interpro domain(s) IPR000916 Bet v I allergen	0.87	1.33E-05	Kirola	GO:0006952; GO:0009607
Solyc04g015030.2	Metal ion binding protein (AHRD V1 B6SZL4_MAIZE); contains Interpro domain(s) IPR006121 Heavy metal transport/detoxification protein	0.87	9.67E-06	Neurofilament medium polypeptide-like	GO:0030001; GO:0046872
Solyc04g074990.2	Zinc finger-homeodomain protein 1 (Fragment) (AHRD *--- B0LK17_YUCFI); contains Interpro domain(s) IPR006456 ZF-HD homeobox protein Cys/His-rich dimerisation region	0.87	9.64E-05	Zinc-finger homeodomain protein 2-	GO:0003677; GO:0045449
Solyc04g082950.2	Unknown Protein (AHRD V1); contains Interpro IPR008166 Protein of unknown function DUF23	0.87	1.00E-03	PREDICTED: uncharacterized protein LOC101247215	
Solyc05g010320.2	Chalcone--flavonone isomerase (AHRD V1 ***- D7LV11_ARALY); Interpro domain(s) IPR003466 Chalcone isomerase, subgroup	0.87	2.14E-03	Chalcone isomerase	GO:0042398
Solyc05g013010.2	Sulfotransferase family protein (AHRD V1 **** contains Interpro domain(s) IPR000863 Sulfotransferase	0.87	6.41E-04	Cytosolic sulfotransferase 5-	GO:0008146
Solyc09g082510.2	Kinase interacting family protein (AHRD V1 D7KCB2_ARALY); contains Interpro domain(s) IPR011684 KIP1-like	0.87	2.11E-04	Intracellular protein transport protein uso1-	
Solyc10g074540.1	Expressed protein (Fragment) (AHRD V1 ***- contains Interpro domain(s) IPR006766 Phosphate-induced protein 1 conserved region	0.87	2.43E-04	Protein exordium-	
Solyc01g079110.2	Histone H3 (AHRD V1 ***- D8L9K7_WHEAT); Interpro domain(s) IPR000164 Histone H3	0.86	2.25E-03	Histone	GO:0003677

Solyc02g068070.2	Lipase (AHRD V1 **** Q1XBG1_RICCO); contains domain(s) IPR006693 AB-hydrolase associated lipase region	0.86	2.89E-03	Triacylglycerol lipase 2-	GO:0006629; GO:0004806
Solyc02g093100.2	Receptor like kinase, RLK	0.86	9.89E-04	Probable inactive receptor kinase at5g67200	GO:0004674; GO:0006468
Solyc03g111680.2	DNA polymerase III gamma/tau subunit (AHRD *-*- B0JGE9_MICAN); contains Interpro domain(s) IPR012763 DNA polymerase III, subunit gamma/ tau	0.86	4.59E-04	Protein stichel-	GO:0003887; GO:0005524; GO:0006260; GO:0009360
Solyc05g012950.1	Sulfotransferase family protein (AHRD V1 **** contains Interpro domain(s) IPR000863 Sulfotransferase	0.86	1.48E-03	Cytosolic sulfotransferase 5-like	GO:0008146
Solyc06g064820.2	GDSL esterase/lipase At1g71691 (AHRD V1 ***- contains Interpro domain(s) IPR001087 Lipase, GDSL	0.86	1.39E-03	Gdsl esterase lipase at1g71691-	GO:0006629; GO:0016788
Solyc11g066130.1	Thaumatin-like protein (AHRD V1 **-- D2KU75_CITJA); Interpro domain(s) IPR017949 Thaumatin, conserved site IPR001938 Thaumatin, pathogenesis-related	0.86	8.26E-04	Osmotin-like protein	
Solyc01g080290.2	Plasma membrane associated protein-like (AHRD V1 Q8LG79_ARATH); contains Interpro domain(s) IPR008390 AWPM-19-like	0.85	1.88E-03	Awpm-19-like protein	
Solyc02g071710.2	GDSL esterase/lipase At1g29670 (AHRD V1 ***- contains Interpro domain(s) IPR001087 Lipase, GDSL	0.85	7.33E-04	Gdsl esterase lipase	GO:0006629; GO:0016788
Solyc02g090580.2	Os03g0859900 protein (Fragment) (AHRD V1 *-*- contains Interpro domain(s) IPR006869 Protein of unknown function DUF547	0.85	8.70E-05	Kinesin-like protein kif11	
Solyc04g008230.2	Polygalacturonase (AHRD V1 ***- B6TYD6_MAIZE); contains domain(s) IPR012334 Pectin lyase fold	0.85	9.30E-05	Polygalacturonase	GO:0005975
Solyc04g008650.2	Receptor like kinase, RLK	0.85	1.11E-03	Inactive leucine-rich repeat receptor-like serine threonine-protein kinase	GO:0004674; GO:0006468
Solyc04g015470.2	Phosphatidylinositol-4-phosphate 5-kinase family protein (AHRD V1 D7L735_ARALY); contains Interpro domain(s) IPR017163 Phosphatidylinositol-4-phosphate 5-kinase, plant	0.85	3.61E-04	Phosphatidylinositol 4-phosphate 5-kinase 1	GO:0016307; GO:0046488
Solyc05g005870.2	Nodulin MtN21 family protein (AHRD V1 D7KXZ8_ARALY); contains Interpro domain(s) IPR000620 Protein of unknown function DUF6, transmembrane	0.85	1.18E-03	Wat1-related protein	GO:0016020
Solyc05g013220.2	Fatty acid elongase 3-ketoacyl-CoA synthase (AHRD **** Q6DUV6_BRANA); contains Interpro domain(s) IPR012392 Very-long-chain 3-ketoacyl-CoA synthase	0.85	2.97E-04	3-ketoacyl- synthase 19	GO:0003824; GO:0008152
Solyc05g049980.2	Glycoside hydrolase family 28 protein/polygalacturonase family (AHRD V1 **-- Q1PF10_ARATH); contains Interpro domain(s) IPR012334 Pectin lyase fold	0.85	1.36E-05	Probable polygalacturonase	GO:0005975
Solyc09g007360.2	Interactor of constitutive active ROPs 3 V1 ***- ICR3_ARATH)	0.85	1.69E-03	Interactor of constitutive active rops chloroplastic-like	
Solyc02g085110.2	Laccase (AHRD V1 ***- Q9AUI0_PINTA); contains domain(s) IPR017761 Laccase	0.84	1.58E-03	Laccase-11	GO:0016491
Solyc03g123620.2	Pectinesterase (AHRD V1 ***- B9GXZ7_POPTR); contains domain(s) IPR000070 Pectinesterase, catalytic	0.84	2.65E-03	Pectinesterase 3	GO:0030599
Solyc05g009660.2	BZIP transcription factor (AHRD V1 **** contains Interpro domain(s) IPR011616 bZIP transcription factor, bZIP-1	0.84	8.64E-04	Transcription factor hbp-1b	GO:0003700; GO:0005634; GO:0006355; GO:0043565; GO:0046983
Solyc05g009990.2	LRR receptor-like serine/threonine-protein kinase, RLP	0.84	3.11E-05	Piriformospora indica-insensitive protein 2	GO:0004553; GO:0005975
Solyc10g084970.1	Unknown Protein (AHRD V1)	0.84	4.88E-05	Unknown Protein	
Solyc05g006020.2	Nucleobase ascorbate transporter (AHRD V1 **-* contains Interpro domain(s) IPR006043 Xanthine/uracil/vitamin C permease	0.83	2.02E-03	Nucleobase-ascorbate transporter 7	GO:0005215; GO:0006810; GO:0016020
Solyc06g009780.2	Kinesin (AHRD V1 ***- Q5MNW6_GOSHI); contains domain(s) IPR001752 Kinesin, motor region	0.83	5.58E-05	Kinesin-4	GO:0003777; GO:0005524; GO:0007018
Solyc08g008610.2	Hydrolase alpha/beta fold family protein (AHRD ***- D7M8X4_ARALY); contains Interpro domain(s) IPR000073 Alpha/beta hydrolase fold-1	0.83	1.95E-03	Alpha beta-hydrolases superfamily protein	
Solyc08g014430.2	Formin 3 (AHRD V1 *-*- D0QAN4_ARATH); Interpro domain(s) IPR015425 Actin-binding FH2	0.83	5.81E-04	Formin-like protein 1	GO:0030036
Solyc08g078520.2	Os03g0859900 protein (Fragment) (AHRD V1 *--- contains Interpro domain(s) IPR006869 Protein of unknown function DUF547	0.83	4.65E-04	Unknown Protein	
Solyc10g079090.1	Chaperone protein dnaJ 6 (AHRD V1 B6TT74_MAIZE); contains Interpro domain(s) IPR003095 Heat shock protein DnaJ	0.83	1.15E-03	Chaperone protein dnaj 6	GO:0006457; GO:0031072; GO:0051082

Solyc01g107800.2	Expressed protein (Fragment) (AHRD V1 *-*- contains Interpro domain(s) IPR006514 Protein of unknown function DUF579, plant	0.82	2.24E-03	Protein irx15	
Solyc02g082260.2	Hydroxy-methylglutaryl-coenzyme A reductase (AHRD V1 **** contains Interpro domain(s) IPR004554 Hydroxymethylglutaryl-CoA reductase, class I, catalytic	0.82	2.23E-03	3-hydroxy-3-methylglutaryl-coenzyme a reductase 1	GO:0004420; GO:0015936; GO:0050662; GO:0055114
Solyc02g088820.2	Serine carboxypeptidase K10B2.2 (AHRD V1 **** contains Interpro domain(s) IPR001563 Peptidase S10, serine carboxypeptidase	0.82	2.58E-04	Unknown Protein	GO:0004185; GO:0006508
Solyc02g091890.1	MRNA complete cds clone RAFL24-22-E06 (AHRD *-*- Q67YL8_ARATH)	0.82	9.23E-04	Dentin sialophosphoprotein	
Solyc03g112620.2	Carboxyl-terminal proteinase (AHRD V1 ***- B4FXN3_MAIZE); Interpro domain(s) IPR004314 Protein of unknown function DUF239, plant	0.82	6.70E-04	Unknown Protein	
Solyc03g113450.2	Receptor like kinase, RLK	0.82	8.43E-04	Lrr receptor-like serine threonine-protein kinase fei 2	GO:0004675; GO:0006468
Solyc04g045530.2	DNA primase large subunit (AHRD V1 B5X2T9_SALSA); contains Interpro domain(s) IPR016558 DNA primase, large subunit, eukaryotic	0.82	2.22E-03	Probable dna primase large subunit	GO:0003896; GO:0005658; GO:0006269
Solyc05g014690.1	ATP-dependent DNA helicase (AHRD V1 *-** contains Interpro domain(s) IPR004589 DNA helicase, ATP-dependent, RecQ type	0.82	2.69E-03	Atp-dependent dna helicase q	GO:0000739; GO:0005524; GO:0006310; GO:0000405
Solyc06g075220.1	Fasciclin-like arabinogalactan protein 5 (AHRD V1 A9XTL0_GOSHI); contains Interpro domain(s) IPR000782 FAS1 domain	0.82	2.63E-04	Fasciclin-like arabinogalactan protein 11	GO:0004773
Solyc08g076820.2	BHLH transcription factor (AHRD V1 **** contains Interpro domain(s) IPR011598 Helix-loop-helix DNA-binding	0.82	5.71E-05	Transcription factor bhlh71	GO:0005634; GO:0030528; GO:0045449
Solyc01g065500.2	Phosphoribosylanthranilate transferase (Fragment) (AHRD V1 *--- contains Interpro domain(s) IPR013583 Phosphoribosyltransferase C-terminal, plant	0.81	2.88E-03	Protein quirky	
Solyc01g103530.2	Receptor like kinase, RLK	0.81	2.71E-04	Leucine-rich repeat receptor-serine threonine-protein kinase bam3	GO:0004674; GO:0006468
Solyc02g080880.2	Aspartic proteinase (AHRD V1 **-* Q8L6A8_THECC); Interpro domain(s) IPR001461 Peptidase A1	0.81	1.13E-04	Aspartic proteinase	GO:0006508; GO:0004190
Solyc04g008500.2	C2H2L domain class transcription factor (AHRD **-* D9ZIU0_MALDO); contains Interpro domain(s) IPR007087 Zinc finger, C2H2-type	0.81	3.76E-04	Protein indeterminate-domain chloroplastic	GO:0005622; GO:0008270
Solyc05g007930.2	Beta-1 3-galactosyltransferase-like protein (AHRD V1 **** contains Interpro domain(s) IPR002659 Glycosyl transferase, family 31	0.81	3.98E-04	Beta- -galactosyltransferase 15	GO:0005529; GO:0010488
Solyc06g048620.2	Receptor like kinase, RLK	0.81	1.89E-04	Probably inactive leucine-rich repeat receptor-like protein kinase	GO:0005515; GO:0004675
Solyc07g053950.1	Unknown Protein (AHRD V1)	0.81	6.03E-04	Unknown protein	
Solyc07g056410.2	Receptor like kinase, RLK	0.81	1.66E-03	Leucine-rich repeat receptor-serine threonine-protein kinase	GO:0006468
Solyc08g079740.2	Receptor like kinase, RLK	0.81	2.79E-04	Probable lrr receptor-like serine threonine-protein kinase	GO:0004675; GO:0006468
Solyc01g006540.2	Lipoxygenase (AHRD V1 **** Q96573_SOLLC); contains domain(s) IPR001246 Lipoxygenase, plant	0.8	6.75E-05	Linoleate 13s-lipoxygenase 2-chloroplastic	GO:0005506; GO:0009055; GO:0016165; GO:0055114
Solyc01g096940.2	Receptor like kinase, RLK	0.8	2.10E-05	Probable inactive leucine-rich repeat receptor-like protein kinase	GO:0005515; GO:0004675; GO:0006468
Solyc02g084610.1	LRR receptor-like serine/threonine-protein kinase, RLP	0.8	2.39E-04	Probable lrr receptor-like serine threonine-protein kinase	GO:0005515
Solyc03g111820.2	Sieve element-occluding protein 3 (AHRD V1 B5THF7_MEDTR)	0.8	4.80E-04	Sieve element occlusion a	
Solyc04g077190.2	Endo-1 4-beta-xylanase (AHRD V1 *-** B6SW51_MAIZE); Interpro domain(s) IPR013781 Glycoside hydrolase, subgroup, catalytic core	0.8	3.20E-04	Glycosyl hydrolase family 10 protein carbohydrate-binding domain-containing protein isoform 1	GO:0031176; GO:0016798
Solyc07g049500.2	Argonaute 4-like protein (AHRD V1 ***- contains Interpro domain(s) IPR003165 Stem cell self-renewal protein Piwi	0.8	9.75E-04	Protein argonaute 16	GO:0035197; GO:0003676; GO:0019899
Solyc01g096190.2	Calcium-transporting ATPase (AHRD V1 ***- A8NHF4_COPC7); Interpro domain(s) IPR005782 ATPase, P-type, calcium-transporting	0.79	6.77E-05	Calcium-transporting endoplasmic reticulum-type	GO:0006754; GO:0006812; GO:0015662; GO:0016020
Solyc01g106690.2	Unknown Protein (AHRD V1); contains Interpro IPR010410 Protein of unknown function DUF1005	0.79	7.06E-04	Formin-like protein 18	

Solyc02g071820.2	Receptor like kinase, RLK	0.79	1.01E-03	Probable lrr receptor-like serine threonine-protein kinase	GO:0005515; GO:0016301; GO:0006468
Solyc03g007800.2	Topoisomerase 1-associated factor 1 (AHRD V1 C9S978_VERA1); contains Interpro domain(s) IPR007725 Timeless C-terminal	0.79	2.13E-04	Protein timeless homolog	
Solyc04g081240.2	Auxin response factor 5 (AHRD V1 D9IVB5_SOLLC); contains Interpro domain(s) IPR010525 Auxin response factor	0.79	5.43E-04	Auxin response factor 5	GO:0003677; GO:0005634; GO:0009725; GO:0045449
Solyc07g063850.2	Indole-3-acetic acid-amido synthetase GH3.8 (AHRD V1 B6U4E2_MAIZE); contains Interpro domain(s) IPR004993 GH3 auxin-responsive promoter	0.79	1.91E-03	Indole-3-acetic acid-amido synthetase	GO:0010279
Solyc08g023280.2	Tripartite motif-containing 22 (Predicted) (AHRD V1 B0VXD4_CALJA); contains Interpro domain(s) IPR001357 BRCT	0.79	1.53E-03	Protein breast cancer susceptibility 1 homolog	GO:0005622
Solyc08g079090.2	Laccase-22 (AHRD V1 **-- LAC22_ORYSJ); contains domain(s) IPR001117 Multicopper oxidase, type 1	0.79	2.51E-03	Monocopper oxidase-like protein sku5	GO:0016491
Solyc10g007870.2	Tumor-related protein (AHRD V1 ***- Q40447_9SOLA)	0.79	4.16E-05	Thionin-like protein	
Solyc11g007330.1	cDNA clone J033118E13 full insert sequence V1 **-- B7EU28_ORYSJ); contains Interpro domain(s) IPR007033 Protein of unknown function DUF662	0.79	1.90E-03	Rab6-interacting golgin	
Solyc01g090440.2	Unknown Protein (AHRD V1)	0.78	1.88E-04	Peptidyl-prolyl cis-trans isomerase fkbp20-2	
Solyc01g111570.2	Receptor-like kinase (AHRD V1 *-*- A7VM42_MARPO); Interpro domain(s) IPR002290 Serine/threonine protein kinase	0.78	2.32E-03	Probable receptor-like serine threonine-protein kinase	GO:0004672; GO:0005524; GO:0006468
Solyc02g065240.2	Hydrolase alpha/beta fold family protein (AHRD **-- D7LG04_ARALY); contains Interpro domain(s) IPR000073 Alpha/beta hydrolase fold-1	0.78	1.98E-04	Salicylic acid-binding protein 2	GO:0080030; GO:0080032
Solyc02g070780.2	DNA replication licensing factor MCM3 (AHRD **** Q16NY8_AEDAE); contains Interpro domain(s) IPR008046 MCM protein 3	0.78	6.34E-04	Dna replication licensing factor mcm3 homolog 2	GO:0003677; GO:0005524; GO:0005634; GO:0006270
Solyc03g097380.2	Heavy metal-associated domain containing protein expressed V1 *--- Q7XG81_ORYSJ); contains Interpro domain(s) IPR006121 Heavy metal transport/detoxification protein	0.78	5.80E-05	Neurofilament medium polypeptide	GO:0030001; GO:0046872
Solyc06g071330.2	Nucleobase ascorbate transporter (AHRD V1 **-* contains Interpro domain(s) IPR006043 Xanthine/uracil/vitamin C permease	0.78	1.17E-03	Nucleobase-ascorbate transporter 6	GO:0005215; GO:0006810; GO:0016020
Solyc09g092740.2	Unknown Protein (AHRD V1); contains Interpro IPR010800 Glycine rich	0.78	1.47E-04	Fam10 family protein	GO:0005737
Solyc12g008530.1	Pectinesterase (AHRD V1 ***- B9RKG6_RICCO); contains domain(s) IPR000070 Pectinesterase, catalytic	0.78	4.24E-05	Probable pectinesterase 53	GO:0005618
Solyc01g079240.2	Long-chain-fatty-acid--CoA ligase family protein (AHRD V1 D7LGL1_ARALY); contains Interpro domain(s) IPR000873 AMP-dependent synthetase and ligase	0.77	2.17E-03	Unknown Protein	GO:0003824; GO:0008152
Solyc03g082860.2	Histone-lysine N-methyltransferase (AHRD V1 **-- D8L815_9POAL); Interpro domain(s) IPR001214 SET	0.77	1.96E-03	Histone-lysine n-methyltransferase atxr6	GO:0005515; GO:0008270; GO:0003677
Solyc03g120380.2	Auxin response factor 9 (AHRD V1 ARFI_ARATH); contains Interpro domain(s) IPR003311 AUX/IAA protein	0.77	3.02E-04	Auxin-induced protein aux22	GO:0005634; GO:0045449
Solyc04g078810.2	Remorin (AHRD V1 ***- Q00M66_SOYBN); contains domain(s) IPR005516 Remorin, C-terminal region	0.77	1.47E-03	Remorin family protein isoform 2	
Solyc05g012790.2	Palmitoyltransferase erf2 (AHRD V1 *-*- C5FZI1_NANOT); Interpro domain(s) IPR001594 Zinc finger, DHHC-type	0.77	8.53E-04	Probable protein s-acyltransferase 22	GO:0008270
Solyc09g011050.2	Laccase (AHRD V1 ***- O24042_LIRTU); contains domain(s) IPR011707 Multicopper oxidase, type 3	0.77	2.88E-03	Laccase-17	GO:0016491
Solyc09g074930.2	REF-like stress related protein 1 (AHRD *--- Q6XNP4_HEVBR); contains Interpro domain(s) IPR008802 Rubber elongation factor	0.77	4.56E-04	Stress-related protein	
Solyc12g057080.1	UDP-glucuronosyltransferase (AHRD V1 **-- Q5UB81_ARATH); contains domain(s) IPR002213 UDP-glucuronosyl/UDP-glucosyltransferase	0.77	2.04E-03	7-deoxyloganetin glucosyltransferase	GO:0008152; GO:0016758
Solyc02g030480.2	Cinnamyl alcohol dehydrogenase-like protein (AHRD V1 A9PHZ1_POPTR); contains Interpro domain(s) IPR002085 Alcohol dehydrogenase superfamily, zinc-containing	0.76	1.84E-03	Probable cinnamyl alcohol dehydrogenase 6	GO:0008270; GO:0016491
Solyc02g090070.2	Phototropic-responsive NPH3 family protein (AHRD V1 D7MMF7_ARALY); contains Interpro domain(s) IPR004249 NPH3	0.76	1.14E-03	Btb poz domain-containing protein	GO:0005515
Solyc03g121610.2	Receptor-like kinase (AHRD V1 **** A7VM33_MARPO); Interpro domain(s) IPR002290 Serine/threonine protein kinase	0.76	1.84E-03	Receptor-like serine threonine-protein kinase ale2	GO:0004674; GO:0006468

Solyc05g009820.2	Glycosyltransferase family GT8 protein (AHRD V1 D0ECQ6_POPDE); contains Interpro domain(s) IPR002495 Glycosyl transferase, family 8	0.76	2.85E-04	Probable galacturonosyltransferase-like 3	GO:0016757
Solyc09g015040.1	Os08g0119500 protein (Fragment) (AHRD V1 *-*-	0.76	1.94E-03	Unknown Protein	
Solyc09g092510.2	Unknown Protein (AHRD V1)	0.76	8.13E-04	Unknown Protein	
Solyc11g013270.1	Os01g0841200 protein (Fragment) (AHRD V1 **-- contains Interpro domain(s) IPR004348 Protein of unknown function DUF246, plant	0.76	7.96E-04	Unknown Protein	
Solyc01g006370.2	Glucan synthase like 3 (AHRD V1 D8R8I5_SELML); contains Interpro domain(s) IPR003440 Glycosyl transferase, family 48	0.75	2.73E-03	Callose synthase 3	GO:0000148; GO:0003843; GO:0006075; GO:0016020
Solyc01g079500.2	DNA replication licensing factor (AHRD V1 Q2U020_ASPOR); contains Interpro domain(s) IPR008050 MCM protein 7	0.75	1.13E-03	Dna replication licensing factor mcm7	GO:0003677; GO:0005524; GO:0005634; GO:0006270
Solyc06g008510.2	CDT1a protein (AHRD V1 ***- Q710F0_ARATH); Interpro domain(s) IPR014939 DNA replication factor CDT1-like	0.75	8.60E-04	Cdt1-like protein chloroplastic	GO:0005515; GO:0004693
Solyc06g069820.2	Unknown Protein (AHRD V1); contains Interpro IPR007750 Protein of unknown function DUF674	0.75	1.28E-03	Unknown Protein	
Solyc10g008490.2	Protein phosphatase 2c (AHRD V1 ***- contains Interpro domain(s) IPR015655 Protein phosphatase 2C	0.75	1.09E-03	Probable protein phosphatase 2c	GO:0003824
Solyc12g044840.1	Receptor-like kinase (AHRD V1 *-*- A7VM65_9VIRI); Interpro domain(s) IPR002290 Serine/threonine protein kinase	0.75	2.10E-03	Inactive protein kinase selmodraft	GO:0006468
Solyc02g086650.2	Glucose-6-phosphate/phosphate translocator 2 (AHRD V1 **** contains Interpro domain(s) IPR004696 Tpt phosphate/phosphoenolpyruvate translocator	0.74	1.28E-03	Phosphoenolpyruvate phosphate translocator chloroplastic	GO:0008524; GO:0016020
Solyc03g005460.2	DNA helicase (AHRD V1 **-- C4R4A1_PICPG); Interpro domain(s) IPR000330 SNF2-related	0.74	1.33E-03	Swi snf-related matrix-associated actin-dependent regulator of chromatin subfamily a member 3	GO:0003676; GO:0008270; GO:0016818
Solyc03g078780.1	UDP-glucosyltransferase (AHRD V1 **** C0LNQ9_MAIZE); contains domain(s) IPR002213 UDP-glucuronosyl/UDP-glucosyltransferase	0.74	2.90E-03	Udp-glycosyltransferase 76f1	GO:0008152; GO:0016758
Solyc04g082420.2	BTB/POZ domain-containing protein (AHRD V1 **-- contains Interpro domain(s) IPR013089 Kelch related	0.74	1.55E-03	Btb poz domain-containing protein	GO:0005515
Solyc08g080780.2	SKIP interacting protein 24 (Fragment) (AHRD *--- B8Q8B0_ORYSI)	0.74	2.43E-04	Unknown Protein	
Solyc10g080090.1	HAT family dimerisation domain containing protein (AHRD V1 **-- Q6AVI0_ORYSJ); contains Interpro domain(s) IPR008906 HAT dimerisation	0.74	9.71E-04	Zinc finger bed domain-containing protein daysleeper	GO:0003677
Solyc02g093300.2	DNA polymerase (AHRD V1 **** B9SC90_RICCO); Interpro domain(s) IPR004578 DNA-directed DNA polymerase, family B, pol2	0.73	5.95E-04	Dna polymerase alpha catalytic subunit	GO:0000166; GO:0003677; GO:0003887; GO:0005634; GO:0006260
Solyc03g093460.2	Receptor-like kinase (AHRD V1 ***- C0SW26_SOYBN); Interpro domain(s) IPR002290 Serine/threonine protein kinase	0.73	1.78E-03	Kinase-like protein tmkl1	GO:0006468
Solyc03g118310.2	BHLH transcription factor (AHRD V1 *-** contains Interpro domain(s) IPR011598 Helix-loop-helix DNA-binding	0.73	8.42E-04	Transcription factor ice1	GO:0005634; GO:0030528; GO:0045449
Solyc04g078750.2	Harpin-induced protein (AHRD V1 ***- B6TDG9_MAIZE); Interpro domain(s) IPR010847 Harpin-induced 1	0.73	2.40E-03	Unknown Protein	
Solyc05g051640.2	Receptor like kinase, RLK	0.73	1.29E-03	Leucine-rich repeat receptor-like protein kinase tdr	GO:0004675; GO:0006468
Solyc06g048950.2	Receptor like kinase, RLK	0.73	9.19E-04	Probably inactive leucine-rich repeat receptor-like protein kinase	GO:0004675; GO:0006468
Solyc07g047830.2	Bzip-like transcription factor-like (AHRD V1 *-*- contains Interpro domain(s) IPR006867 Protein of unknown function DUF632	0.73	9.68E-04	Unknown protein	
Solyc07g049550.2	1-aminocyclopropane-1-carboxylate oxidase (AHRD V1 **** Q94F66_SOLTU); Interpro domain(s) IPR005123 Oxoglutarate and iron-dependent oxygenase	0.73	2.25E-04	1-aminocyclopropane-1-carboxylate oxidase	GO:0016491
Solyc08g067240.2	DNA topoisomerase 2-binding protein 1 (AHRD *--- TOPB1_MOUSE); contains Interpro domain(s) IPR001357 BRCT	0.73	8.19E-04	Brct domain-containing dna repair protein	GO:0005622
Solyc08g081120.2	Kinesin-like protein 73641-79546 (AHRD V1 ***-	0.73	5.28E-04	Kinesin kp1	
Solyc09g092460.2	Receptor-like kinase (AHRD V1 **** C0SW31_SOYBN); Interpro domain(s) IPR002290 Serine/threonine protein kinase	0.73	6.03E-04	Probable receptor protein kinase tmk1	GO:0004672; GO:0005524; GO:0006468
Solyc10g085070.1	UPF0503 protein At3g09070, chloroplastic (AHRD V1 U503A_ARATH); contains Interpro domain(s) IPR008004 Protein of unknown function DUF740	0.73	2.09E-03	Upf0503 protein chloroplastic	

Solyc12g014140.1	Transcription factor CYCLOIDEA (Fragment) (AHRD V1 CYCLD_ANTML); contains Interpro domain(s) IPR005333 Transcription factor, TCP	0.73	1.38E-04	Transcription factor tcp4	
Solyc12g056300.1	Receptor like kinase, RLK	0.73	4.92E-04	Probable lrr receptor-like serine threonine-protein kinase	GO:0006468
Solyc02g092930.1	MYB transcription factor (AHRD V1 **** contains Interpro domain(s) IPR015495 Myb transcription factor	0.72	1.46E-03	Transcription factor myb44	GO:0005524; GO:0003700
Solyc04g082710.2	Cathepsin B-like cysteine proteinase 3 (AHRD **-- CPR3_CAEEL); contains Interpro domain(s) IPR013128 Peptidase C1A, papain	0.72	2.69E-04	Xylem cysteine proteinase 1	GO:0004197; GO:0006508
Solyc07g007980.2	ATP binding / serine-threonine kinase (AHRD **** C5DB71_VITVI); contains Interpro domain(s) IPR002290 Serine/threonine protein kinase	0.72	7.18E-04	Probable receptor-like protein kinase	GO:0004672; GO:0005524; GO:0006468
Solyc10g011820.2	Delta-6 desaturase (AHRD V1 **** D2KBG7_RIBNI); Interpro domain(s) IPR012171 Fatty acid/sphingolipid desaturase	0.72	7.12E-04	Delta -fatty-acid desaturase-	GO:0020037
Solyc10g085850.1	TPSI1 (AHRD V1 ***- Q41336_SOLLC)	0.72	1.75E-03	Tpsi1	
Solyc11g069660.1	Nbs-lrr, resistance protein	0.72	1.39E-03	Disease resistance partial	GO:0005524; GO:0006915
Solyc11g071850.1	AT5G22070 protein (Fragment) (AHRD V1 ***- contains Interpro domain(s) IPR004949 Protein of unknown function DUF266, plant	0.72	2.52E-04	Unknown Protein	
Solyc04g073970.2	cDNA clone J033025P19 full insert sequence V1 ***- B7ERC6_ORYSJ)	0.71	1.35E-03	Unknown Protein	
Solyc05g005000.2	Lipase (AHRD V1 **** B6UAB9_MAIZE)	0.71	1.91E-03	Unknown Protein	GO:0004806
Solyc05g014540.2	DNA polymerase alpha subunit B family V1 ***- D7KVM0_ARALY); contains Interpro domain(s) IPR016722 DNA polymerase alpha, subunit B	0.71	7.94E-04	Dna polymerase alpha subunit b	GO:0046982; GO:0006260
Solyc06g050840.2	DNA binding protein (AHRD V1 ***-	0.71	1.33E-03	Transcription factor scream2	GO:0030528
Solyc07g018240.1	Elongation of very long chain fatty protein 4 (AHRD V1 *-** B0W9Y0_CULQU); contains Interpro domain(s) IPR002076 GNS1/SUR4 membrane protein	0.71	7.92E-04	Elongation of fatty acids protein 3	GO:0005515; GO:0009922; GO:0016021
Solyc01g112260.2	Unknown Protein (AHRD V1)	0.7	2.62E-03	Unknown Protein	
Solyc04g079340.2	Os03g0859900 protein (Fragment) (AHRD V1 ***- contains Interpro domain(s) IPR006869 Protein of unknown function DUF547	0.7	6.92E-04	Unknown Protein	
Solyc07g018300.2	Single-stranded DNA binding protein p30 subunit V1 **** C5JUU5_AJEDS); contains Interpro domain(s) IPR014646 Replication protein A, subunit RPA32	0.7	1.50E-03	Replication protein a 32 kda subunit a	GO:0003676
Solyc07g042390.1	Pectinesterase (AHRD V1 **** B7SIX2_9CONI); contains domain(s) IPR006501 Pectinesterase inhibitor	0.7	1.31E-03	Pectinesterase	GO:0004857; GO:0030599
Solyc10g081250.1	DNA polymerase (AHRD V1 ***- C5Y6L0_SORBI); Interpro domain(s) IPR004578 DNA-directed DNA polymerase, family B, pol2	0.7	1.36E-03	Dna polymerase delta catalytic subunit	GO:0000166; GO:0003677; GO:0003887; GO:0005634; GO:0006260
Solyc12g044310.1	Solute carrier family 15 member 4 V1 **-- S15A4_XENLA); contains Interpro domain(s) IPR000109 TGF-beta receptor, type I/II extracellular region	0.7	1.57E-03	Protein nrt1 ptr family	GO:0016020
Solyc04g082110.2	Rop guanine nucleotide exchange factor 1 V1 **-* ROGF1_ARATH); contains Interpro domain(s) IPR005512 Rop nucleotide exchanger, PRONE	0.69	2.01E-03	Rop guanine nucleotide exchange factor 1-	GO:0005089
Solyc05g026240.1	LRR receptor-like serine/threonine-protein kinase, RLP	0.69	1.96E-03	Leucine-rich repeat extensin-protein 4	GO:0005515; GO:0005199; GO:0004675
Solyc06g074850.2	Serine carboxypeptidase (AHRD V1 **** Q2Z1Y2_PRUMU); Interpro domain(s) IPR001563 Peptidase S10, serine carboxypeptidase	0.69	1.83E-03	Serine carboxypeptidase	GO:0004185; GO:0006508
Solyc08g061560.2	Receptor like kinase, RLK	0.69	6.62E-04	Lrr receptor-like serine threonine-protein kinase erecta	GO:0005515; GO:0004675
Solyc08g079650.2	ACT domain containing protein expressed (AHRD **-- Q10P43_ORYSJ); contains Interpro domain(s) IPR002912 Amino acid-binding ACT	0.69	7.23E-04	Amino acid binding	GO:0008152; GO:0016597
Solyc09g011960.1	Laccase (AHRD V1 ***- O24042_LIRTU); contains domain(s) IPR017761 Laccase	0.69	1.31E-03	Laccase-17	GO:0016491
Solyc09g090020.2	Germin-like protein 5 (AHRD V1 ***- contains Interpro domain(s) IPR014710 RmlC-like jelly roll fold	0.69	7.01E-04	Germin-like protein subfamily 1 member 17	GO:0030145; GO:0048046
Solyc10g005960.1	Fasciclin-like arabinogalactan protein 10 (AHRD V1 A9XTL5_GOSHI); contains Interpro domain(s) IPR000782 FAS1 domain	0.69	1.38E-03	Fasciclin-like arabinogalactan protein 1	
Solyc01g102390.2	Germin-like protein (AHRD V1 ***- O65358_SOLTU); Interpro domain(s) IPR014710 RmlC-like jelly roll fold	0.68	3.96E-04	Nectarin-1	GO:0030145; GO:0048046

Solyc02g024070.2	Class III homeodomain-leucine zipper (AHRD V1 Q1WD30_GINBI); contains Interpro domain(s) IPR013978 MEKHLA	0.68	1.74E-03	Homeobox-leucine zipper protein athb-14	GO:0003677; GO:0045449
Solyc05g005170.2	Polygalacturonase 2 (AHRD V1 ***- A8D0T9_ONCHC); Interpro domain(s) IPR012334 Pectin lyase fold	0.68	4.73E-04	Polygalacturonase	GO:0004650; GO:0005975
Solyc06g068770.2	Exostosin-like glycosyltransferase (AHRD V1 **-- A8I7L6_CHLRE); Interpro domain(s) IPR004263 Exostosin-like	0.68	9.54E-04	Probable beta- -xylosyltransferase	GO:0016020
Solyc07g008670.2	AT1G74160 protein (Fragment) (AHRD V1 *-*-	0.68	1.71E-03	Protein longifolia 1-	
Solyc10g074920.1	Mannan endo-1 4-beta-mannosidase (AHRD V1 ***- contains Interpro domain(s) IPR001547 Glycoside hydrolase, family 5	0.68	4.81E-04	Mannan endo- -beta-mannosidase	GO:0003824; GO:0005975; GO:0043169
Solyc10g083670.1	Cellulose synthase-like C2 glycosyltransferase family 2 V1 **-* A9RNK0_PHYPA); contains Interpro domain(s) IPR001173 Glycosyl transferase, family 2	0.68	2.58E-03	Glucomannan 4-beta-mannosyltransferase 9	GO:0051753
Solyc01g096040.2	Aspartic proteinase nepenthesin I (AHRD V1 A9ZMF9_NEPAL); contains Interpro domain(s) IPR001461 Peptidase A1	0.67	2.29E-03	Protein aspartic protease in guard cell 2	GO:0004190; GO:0006508
Solyc03g098290.2	Sucrose synthase (AHRD V1 **** Q9FHU4_ARATH); Interpro domain(s) IPR012820 Sucrose synthase, plants and cyanobacteria	0.67	1.53E-03	Sucrose synthase 6	GO:0009058
Solyc05g013690.2	GDSL esterase/lipase At3g26430 (AHRD V1 ***- contains Interpro domain(s) IPR001087 Lipase, GDSL	0.67	1.77E-03	Gdsl esterase lipase at3g26430-	GO:0006629; GO:0016788
Solyc08g066490.2	Receptor like kinase, RLK	0.67	2.41E-03	Probably inactive leucine-rich repeat receptor-like protein kinase	GO:0004675; GO:0006468
Solyc09g075360.2	Endoglucanase 1 (AHRD V1 ***- B6U0P7_MAIZE); Interpro domain(s) IPR008928 Six-hairpin glycosidase-like IPR012341 Six-hairpin glycosidase IPR018221 Glycoside hydrolase, family 9, active	0.67	2.64E-03	Endoglucanase 17	GO:0004553; GO:0005975
Solyc09g083400.2	Os02g0200800 protein (Fragment) (AHRD V1 *-*- contains Interpro domain(s) IPR009675 Targeting for Xklp2	0.67	2.72E-03	Protein wvd2-like 1	
Solyc11g072490.1	Interactor of constitutive active ROPs 3 V1 ***- ICR3_ARATH)	0.67	1.50E-03	Interactor of constitutive active rops 3-like	
Solyc01g098740.2	Receptor protein kinase-like protein (AHRD V1 Q9LJU5_ARATH); contains Interpro domain(s) IPR002290 Serine/threonine protein kinase	0.66	1.34E-03	Pto-interacting protein 1	GO:0004672; GO:0005524; GO:0006468
Solyc01g110960.2	Glutamic acid-rich protein (AHRD V1 ***-	0.66	1.82E-03	Prefoldin chaperone subunit family	
Solyc08g080720.2	Selenoprotein H (AHRD V1 *-*- A8JGP7_CHLRE); Interpro domain(s) IPR011893 SelT/selW/selH selenoprotein	0.66	2.87E-03	Selenoprotein h	GO:0008430; GO:0045454
Solyc09g082500.2	Tesmin/TSO1-like CXC domain containing protein expressed V1 *--- Q10G99_ORYSJ); contains Interpro domain(s) IPR005172 Tesmin/TSO1-like, CXC	0.66	1.37E-03	Protein tesmin tso1-like cxc 2	GO:0003700
Solyc11g044940.1	Pto-like, Serine/threonine kinase protein, resistance protein	0.66	1.02E-03	Serine threonine-protein kinase-like protein acr4	GO:0004672; GO:0005524; GO:0006468
Solyc02g072240.2	Cellulose synthase (AHRD V1 **** Q6J8W9_9ROSI); Interpro domain(s) IPR005150 Cellulose synthase	0.65	3.92E-04	Cellulose synthase a catalytic subunit 8	GO:0016020; GO:0016760; GO:0030244
Solyc07g062680.1	Transcription factor CYCLOIDEA (Fragment) (AHRD V1 CYCLD_ANTML); contains Interpro domain(s) IPR017887 Transcription factor TCP subgroup	0.65	1.28E-03	Transcription factor tcp4	GO:0003700
Solyc09g011690.2	Unknown Protein (AHRD V1)	0.65	1.85E-03	Unknown Protein	
Solyc12g008490.1	Ceramide glucosyltransferase (AHRD V1 ***- Q946Y1_GOSAR)	0.65	1.57E-03	Nucleotide-diphospho-sugar transferases superfamily protein isoform 1	
Solyc02g084390.2	Kinesin like protein (AHRD V1 *-*- contains Interpro domain(s) IPR001752 Kinesin, motor region	0.64	1.45E-03	Kinesin-like protein nack1	GO:0003777; GO:0005524; GO:0007018
Solyc03g096800.2	At5g24290-like protein (Fragment) (AHRD V1 *-*- contains Interpro domain(s) IPR008217 Protein of unknown function DUF125, transmembrane	0.64	1.69E-03	Membrane protein of er body-like protein isoform	
Solyc06g072700.2	Metal ion binding protein (AHRD V1 B6U064_MAIZE); contains Interpro domain(s) IPR006121 Heavy metal transport/detoxification protein	0.64	1.21E-03	Neurofilament medium polypeptide	GO:0030001; GO:0046872
Solyc02g092670.1	Subtilisin-like protease (AHRD V1 ***- A9XG40_TOBAC); Interpro domain(s) IPR015500 Peptidase S8, subtilisin-related	0.63	9.98E-04	Subtilisin-like protease	GO:0004252; GO:0006508
Solyc12g088760.1	Subtilisin-like protease (AHRD V1 **-- Q38708_ALNGL); Interpro domain(s) IPR015500 Peptidase S8, subtilisin-related	0.63	2.05E-03	Subtilisin-like protease	GO:0004252; GO:0042802; GO:0043086
Solyc05g010400.2	Receptor like kinase, RLK	0.62	1.86E-03	Protein nsp-interacting kinase 2	GO:0004672; GO:0005524; GO:0006468

Solyc07g062580.2	DnaJ homolog subfamily C member 7 V1 *--- B5X1N5_SALSA); contains Interpro domain(s) IPR015609 Molecular chaperone, heat shock protein, Hsp40, DnaJ	0.62	2.59E-03	Tpr repeat-containing thioredoxin ttl1	GO:0045454
Solyc09g011160.2	Regulator of chromosome condensation RCC1 (AHRD *--- B8FLG8_DESAA); contains Interpro domain(s) IPR009091 Regulator of chromosome condensation/beta-lactamase-inhibitor protein II	0.62	2.83E-03	Ultraviolet-b receptor uvr8-like	GO:0003682; GO:0008536
Solyc01g110580.2	Auxin-induced SAUR-like protein (AHRD V1 **-- contains Interpro domain(s) IPR003676 Auxin responsive SAUR protein	0.61	2.73E-03	Auxin-induced protein 15a	GO:0005516
Solyc03g019890.2	Beta-galactosidase (AHRD V1 ***- A2JGX1_SOLLC); contains domain(s) IPR001944 Glycoside hydrolase, family 35	0.61	2.77E-03	Beta-galactosidase 10	GO:0005975; GO:0005515
Solyc04g078700.2	Receptor-like protein kinase (AHRD V1 ***- contains Interpro domain(s) IPR002290 Serine/threonine protein kinase	0.61	2.21E-03	Probable serine threonine-protein kinase	GO:0004672; GO:0005524; GO:0006468
Solyc07g021700.2	N-myc downstream regulated (Fragment) (AHRD V1 Q177C7_AEDAE); contains Interpro domain(s) IPR015511 Pollen specific protein SF21	0.61	2.78E-03	Pollen-specific protein sf21	GO:0005515
Solyc09g097890.2	Membrane protein (AHRD V1 **-- B6ST21_MAIZE); Interpro domain(s) IPR006593 Cytochrome b561/ferric reductase transmembrane	0.61	2.03E-03	Cytochrome b561 and domon domain-containing protein	
Solyc04g009180.1	TCP family transcription factor (AHRD V1 A6MCZ2_9ORYZ); contains Interpro domain(s) IPR005333 Transcription factor, TCP	0.6	1.95E-03	Transcription factor tcp7	GO:0003700
Solyc05g009680.1	Aspartic proteinase nepenthesin I (AHRD V1 A9ZMF9_NEPAL); contains Interpro domain(s) IPR001461 Peptidase A1	0.6	2.82E-03	Protein aspartic protease in guard cell 1	GO:0004190; GO:0006508
Solyc07g006710.1	Pathogenesis-related protein PR-1 (AHRD V1 **-- contains Interpro domain(s) IPR002413 Ves allergen	0.6	2.10E-03	Pathogenesis-related protein pr-1	GO:0005515; GO:0005576
Solyc10g086660.1	Rop guanine nucleotide exchange factor 1 V1 ***- ROGF1_ARATH); contains Interpro domain(s) IPR005512 Rop nucleotide exchanger, PRONE	0.6	1.70E-03	Rop guanine nucleotide exchange factor 7	GO:0005089
Solyc01g107650.2	Receptor like kinase, RLK	0.59	2.24E-03	Probable lrr receptor-like serine threonine-protein kinase	GO:0004672; GO:0005524; GO:0006468
Solyc03g110880.2	DNA-directed RNA polymerase (AHRD V1 ***- contains Interpro domain(s) IPR015712 DNA-directed RNA polymerase, subunit 2	0.59	2.81E-03	Unknown Protein	GO:0003677; GO:0003899; GO:0006350

Supplementary Table 4. List of differentially down-regulated genes identified by RNA-seq upon exposition of receiver plants to Sys-treated plants.

The table reports the locus id, the gene description, the log_2 of the fold change (log2FC), the FDR of the differential expression, the Best Blast Hit Descriptor and the GO IDs.

Locus	Gene Description (ITAG 2.5)	Log2FC	FDR	Best Blast Hit Descriptor	GO ID
Solyc10g052580.1	Auxin-induced SAUR-like protein (AHRD V1 ***- contains Interpro domain(s) IPR003676 Auxin responsive SAUR protein	-9.82	5.51E-08	Auxin-induced protein	GO:0005516
Solyc06g035940.2	Homeobox-leucine zipper protein PROTODERMAL FACTOR 2 V1 **** PDF2_ARATH); contains Interpro domain(s) IPR002913 Lipid-binding START	-9.14	2.60E-08	Homeobox-leucine zipper protein anthocyaninless 2	GO:0003677; GO:0045449
Solyc10g052570.1	Auxin-responsive protein (AHRD V1 ***- A8IXW9_BRACM); Interpro domain(s) IPR003676 Auxin responsive SAUR protein	-6.96	3.97E-05	Auxin-induced protein 6b	
Solyc04g052890.1	Auxin-responsive protein (AHRD V1 ***- A8IXW9_BRACM); Interpro domain(s) IPR003676 Auxin responsive SAUR protein	-6.68	7.83E-05	Auxin-induced protein 6b	
Solyc09g089490.2	Proteinase inhibitor I (AHRD V1 ***- contains Interpro domain(s) IPR000864 Proteinase inhibitor I13, potato inhibitor I	-6.36	7.82E-06	Proteinase inhibitor i-b	GO:0004867; GO:0009611
Solyc02g083480.2	Peroxidase (AHRD V1 ***- A9PGX3_POPTR); contains domain(s) IPR002016 Haem peroxidase, plant/fungal/bacterial	-5.3	5.79E-07	Peroxidase 64	GO:0004601; GO:0006979; GO:0020037; GO:0055114
Solyc12g006730.1	Unknown Protein (AHRD V1)	-5.02	2.91E-05	Unknown Protein	
Solyc12g006750.1	Unknown Protein (AHRD V1)	-4.79	9.15E-05	Unknown Protein	
Solyc02g005050.2	Unknown Protein (AHRD V1)	-4.77	6.65E-04	Unknown Protein	
Solyc00g020540.1	Aminotransferase-like protein (AHRD V1 *-*- Q6EQM2_ORYSJ); Interpro domain(s) IPR019557 Aminotransferase-like, plant mobile domain	-4.71	1.87E-04	Unknown Protein	
Solyc09g018200.1	Plant-specific domain TIGR01568 family protein (AHRD *--- B6U561_MAIZE); contains Interpro domain(s) IPR006458 Protein of unknown function DUF623, plant	-4.58	1.02E-03	Transcription repressor ofp1	
Solyc05g051720.1	Glutaredoxin (AHRD V1 ***- B9I9V9_POPTR); contains domain(s) IPR011905 Glutaredoxin-like, plant II	-4.57	5.01E-06	Monothiol glutaredoxin-s1	GO:0045454
Solyc07g044900.1	Unknown Protein (AHRD V1)	-4.24	9.24E-04	Unknown Protein	
Solyc08g013950.1	Unknown Protein (AHRD V1)	-4.11	4.84E-06	Unknown Protein	
Solyc07g052070.1	Cytochrome P450	-4.01	5.67E-04	Cytochrome p450 cyp72a219	
Solyc02g067750.2	Carbonic anhydrase (AHRD V1 ***- B1VK36_SOLLC); Interpro domain(s) IPR001765 Carbonic anhydrase	-3.85	9.09E-05	Carbonic chloroplastic	GO:0004089; GO:0008270; GO:0015976
Solyc02g061780.2	NAC domain transcription factor (AHRD V1 Q5DM35_WHEAT); contains Interpro domain(s) IPR003441 No apical meristem (NAM) protein	-3.82	1.43E-25	Nac domain-containing protein 94	GO:0003677; GO:0045449
Solyc04g011790.1	Glutaredoxin (AHRD V1 **** B9I9V9_POPTR); contains domain(s) IPR011905 Glutaredoxin-like, plant II	-3.59	7.63E-06	Monothiol glutaredoxin-s1	GO:0045454; GO:0008794
Solyc09g008750.1	Unknown Protein (AHRD V1)	-3.53	9.44E-09	Vq motif-containing protein 29	
Solyc04g071780.2	Cytochrome P450	-3.42	4.46E-09	Cytochrome p450 71a1	GO:0004497; GO:0005506; GO:0020037
Solyc02g079480.1	FAD-binding domain-containing protein (AHRD V1 **-- contains Interpro domain(s) IPR006094 FAD linked oxidase, N-terminal	-3.35	1.31E-07	Tetrahydrocannabinolic acid synthase	GO:0016491; GO:0050660
Solyc09g097960.2	Aldo/keto reductase family protein (AHRD V1 D7KWQ9_ARALY); contains Interpro domain(s) IPR001395 Aldo/keto reductase	-3.34	3.91E-05	Auxin-induced protein pcnt115	GO:0016491; GO:0055114
Solyc09g082810.2	Unknown Protein (AHRD V1); contains Interpro IPR010800 Glycine rich	-3.28	3.53E-26	S14981extensin class i (clone w1-8 l) - tomato	
Solyc05g051730.1	Glutaredoxin (AHRD V1 **** B9I9V9_POPTR); contains domain(s) IPR011905 Glutaredoxin-like, plant II	-3.24	1.03E-04	Monothiol glutaredoxin-s1	GO:0045454; GO:0008794
Solyc05g052400.2	Laccase (AHRD V1 ***- Q9ZP47_POPTR); contains domain(s) IPR017761 Laccase	-3.16	3.00E-04	Laccase-12	GO:0016491
Solyc02g071560.2	Subtilisin-like protease (AHRD V1 ***- Q9FK77_ARATH); Interpro domain(s) IPR015500 Peptidase S8, subtilisin-related	-3.08	2.23E-07	Subtilisin-like protease	GO:0004252; GO:0042802; GO:0043086

Solyc05g052670.1	N-hydroxycinnamoyl/benzoyltransferase 1 (AHRD V1 **-- Q00M72_SOYBN); Interpro domain(s) IPR003480 Transferase	-3	2.81E-19	Uncharacterized acetyltransferase	GO:0016747
Solyc06g051860.1	Inorganic phosphate transporter 6 (AHRD V1 Q5CC71_MAIZE); contains Interpro domain(s) IPR016196 Major facilitator superfamily, general substrate transporter	-2.92	6.22E-07	Inorganic phosphate transporter 1-11	GO:0005315; GO:0005515
Solyc05g012850.2	Unknown Protein (AHRD V1)	-2.91	4.69E-05	Unknown Protein	
Solyc12g057020.1	Acetyl xylan esterase A (AHRD V1 D0TLQ4_9BACE); contains Interpro domain(s) IPR005181 Protein of unknown function DUF303, acetylesterase putative	-2.89	6.00E-05	Probable carbohydrate esterase at4g34215	
Solyc02g071470.2	1-aminocyclopropane-1-carboxylate oxidase 1 (AHRD V1 **-* contains Interpro domain(s) IPR005123 Oxoglutarate and iron-dependent oxygenase	-2.87	8.03E-08	Protein srg1	GO:0016491
Solyc03g116620.2	Phospholipase D (AHRD V1 **** D2E4A5_9ROSI); Interpro domain(s) IPR011402 Phospholipase D, plant	-2.82	1.73E-04	Phospholipase d alpha 1	GO:0005515; GO:0004630; GO:0008152
Solyc08g080670.1	Osmotin-like protein (Fragment) (AHRD V1 **-- contains Interpro domain(s) IPR001938 Thaumatin, pathogenesis-related	-2.74	9.54E-04	Osmotin-like protein	GO:0005515
Solyc09g007010.1	Pathogenesis related protein PR-1 (AHRD V1 Q9SC15_SOLTU); contains Interpro domain(s) IPR002413 Ves allergen	-2.74	2.62E-35	Pathogenesis-related leaf protein 4	GO:0005515; GO:0005576
Solyc12g094610.1	U-box domain-containing protein 15 (AHRD V1 PUB15_ARATH); contains Interpro domain(s) IPR011989 Armadillo-like helical	-2.73	2.93E-04	U-box domain-containing protein 15	GO:0005488
Solyc07g052140.2	(-)-germacrene D synthase (AHRD V1 ***- contains Interpro domain(s) IPR005630 Terpene synthase, metal-binding domain	-2.72	3.09E-08	(-)-Germacrene d synthase-	GO:0016829
Solyc03g033750.1	BCS1 protein-like protein (AHRD V1 ***-	-2.65	1.45E-04	Probable mitochondrial chaperone bcs1-a	
Solyc12g088240.1	Cellulose synthase-like C1-1 glycosyltransferase family 2 (AHRD V1 ***- D8RV47_SELML); contains Interpro domain(s) IPR001173 Glycosyl transferase, family 2	-2.62	1.63E-03	Probable xyloglucan glycosyltransferase 12	
Solyc02g032660.2	MATE efflux family protein expressed (AHRD **-- Q10HY1_ORYSJ); contains Interpro domain(s) IPR002528 Multi antimicrobial extrusion protein MatE	-2.6	6.09E-10	Protein transparent testa 12-like	GO:0006855; GO:0015238; GO:0015297; GO:0016020
Solyc08g080640.1	Osmotin-like protein (Fragment) (AHRD V1 **-- contains Interpro domain(s) IPR017949 Thaumatin, conserved site IPR001938 Thaumatin, pathogenesis-related	-2.58	6.39E-19	Osmotin-like protein	GO:0005515
Solyc08g080650.1	Osmotin-like protein (Fragment) (AHRD V1 **-- contains Interpro domain(s) IPR001938 Thaumatin, pathogenesis-related	-2.53	2.63E-25	Osmotin-like protein	GO:0005515
Solyc01g103920.2	Ferredoxin I (AHRD V1 ***- Q93XJ9_SOLTU); Interpro domain(s) IPR010241 Ferredoxin [2Fe-2S], plant	-2.52	8.06E-07	Ferredoxin	GO:0005515; GO:0051536
Solyc00g174340.1	Pathogenesis-related protein 1b (AHRD V1 ***- contains Interpro domain(s) IPR018244 Allergen V5/Tpx-1 related, conserved site	-2.51	5.88E-27	Unknown Protein	GO:0005515; GO:0005576
Solyc05g009170.1	Zinc finger protein 6 (AHRD V1 B6T066_MAIZE); contains Interpro domain(s) IPR007087 Zinc finger, C2H2-type	-2.49	6.69E-05	Zinc finger protein 6	GO:0005622; GO:0008270
Solyc07g044910.1	Unknown Protein (AHRD V1)	-2.49	1.79E-03	Unknown Protein	
Solyc02g086700.2	Beta-1 3-glucanase (AHRD V1 ***- Q9SYX6_TOBAC); Interpro domain(s) IPR000490 Glycoside hydrolase, family 17	-2.47	2.51E-16	Glucan endo- -beta-glucosidase	GO:0003824; GO:0005975; GO:0043169
Solyc09g059220.1	Unknown Protein (AHRD V1)	-2.44	2.02E-03	Unknown Protein	
Solyc07g049530.2	1-aminocyclopropane-1-carboxylate oxidase (AHRD V1 ***- Q94F66_SOLTU); Interpro domain(s) IPR005123 Oxoglutarate and iron-dependent oxygenase	-2.4	6.25E-28	1-aminocyclopropane-1-carboxylate oxidase 1	GO:0016491
Solyc08g080620.1	Osmotin-like protein (Fragment) (AHRD V1 **-- contains Interpro domain(s) IPR017949 Thaumatin, conserved site IPR001938 Thaumatin, pathogenesis-related	-2.38	6.10E-06	Osmotin-like protein	GO:0005515
Solyc12g042010.1	Unknown Protein (AHRD V1); contains Interpro IPR011598 Helix-loop-helix DNA-binding	-2.37	2.77E-05	Transcription factor ibh1	GO:0005634; GO:0030528; GO:0045449
Solyc09g010990.2	Laccase (AHRD V1 ***- O24044_LIRTU); contains domain(s) IPR017761 Laccase	-2.35	1.14E-04	Laccase-17	GO:0016491
Solyc11g069700.1	Elongation factor 1-alpha (AHRD V1 ***- contains Interpro domain(s) IPR004539 Translation elongation factor EF1A, eukaryotic and archaeal	-2.35	6.03E-12	Elongation factor 1-alpha	GO:0003746; GO:0005525; GO:0005737; GO:0006414
Solyc08g081470.2	Nitrilase-associated protein (AHRD V1 --*- B6SPJ4_MAIZE)	-2.33	6.95E-06	Protein spiral1	
Solyc00g174330.2	Pathogenesis related protein PR-1 (AHRD V1 Q9SC15_SOLTU); contains Interpro domain(s) IPR001283 Allergen V5/Tpx-1 related	-2.32	7.49E-22	Unknown Protein	GO:0005576

Solyc07g064650.1	Arabidopsis thaliana genomic DNA chromosome 5 clone MOK16 (AHRD V1 *--- Q9LYW2_ARATH); contains Interpro domain(s) IPR007608 Protein of unknown function	-2.31	1.46E-03	Phospholipase d c	GO:0005515
Solyc04g072700.2	Metal ion binding protein (AHRD V1 B6U811_MAIZE); contains Interpro domain(s) IPR006121 Heavy metal transport/detoxification protein	-2.3	1.78E-08	Heavy metal-associated isoprenylated plant protein 26	GO:0030001; GO:0046872
Solyc02g082920.2	Endochitinase (Chitinase) (AHRD V1 **** Q43184_SOLTU); Interpro domain(s) IPR000726 Glycoside hydrolase, family 19, catalytic	-2.29	1.66E-19	Class ii chitinase	GO:0004568; GO:0006032; GO:0016998
Solyc01g105810.2	Rab-GDP dissociation inhibitor (AHRD V1 **** contains Interpro domain(s) IPR000806 Rab GDI protein	-2.27	1.36E-03	Guanosine nucleotide diphosphate dissociation inhibitor	GO:0005093; GO:0015031
Solyc09g089930.1	Ethylene responsive transcription factor 1a (AHRD *-** C0J9I9_9ROSA); contains Interpro domain(s) IPR001471 Pathogenesis-related transcriptional factor and ERF, DNA-binding	-2.27	1.59E-16	Ethylene-responsive transcription factor 1b	GO:0003700; GO:0006355
Solyc02g064690.2	Acetyltransferase-like protein (AHRD V1 ***- Q7EYF5_ORYSJ); Interpro domain(s) IPR000182 GCN5-related N-acetyltransferase	-2.26	3.36E-09	Probable n-acetyltransferase hls1	GO:0008152
Solyc09g008760.1	Unknown Protein (AHRD V1); contains Interpro IPR008889 VQ	-2.24	1.12E-06	Serine arginine repetitive matrix protein 2	
Solyc11g066270.1	Xyloglucan endotransglucosylase/hydrolase 9 (AHRD V1 ***- contains Interpro domain(s) IPR016455 Xyloglucan endotransglucosylase/hydrolase	-2.24	1.23E-04	Probable xyloglucan endotransglucosylase hydrolase protein 31	GO:0004553; GO:0005975
Solyc03g098760.1	Kunitz-type protease inhibitor-like protein (AHRD V1 Q2XPY0_SOLTU); contains Interpro domain(s) IPR011065 Kunitz inhibitor ST1-like	-2.17	1.28E-03	Kunitz-type inhibitor b	GO:0004866
Solyc12g013830.1	Unknown Protein (AHRD V1)	-2.16	1.38E-04	Unknown Protein	
Solyc01g087810.2	Subtilisin-like protease (AHRD V1 **-- O82777_SOLLC); Interpro domain(s) IPR015500 Peptidase S8, subtilisin-related	-2.14	8.52E-18	Subtilisin-like protease	GO:0006508
Solyc02g061770.2	Endochitinase (Chitinase) (AHRD V1 **** Q43184_SOLTU); Interpro domain(s) IPR000726 Glycoside hydrolase, family 19, catalytic	-2.14	7.08E-04	Basic endochitinase	GO:0004568; GO:0006032; GO:0016998
Solyc09g005730.2	Plant-specific domain TIGR01589 family protein (AHRD ***- B4FFL2_MAIZE); contains Interpro domain(s) IPR006476 Conserved hypothetical protein CHP01589, plant	-2.14	1.89E-06	Math and lrr domain-containing protein	
Solyc07g008520.2	Peptide transporter (AHRD V1 **-* O48542_HORVU); Interpro domain(s) IPR000109 TGF-beta receptor, type I/II extracellular region	-2.12	1.27E-05	Protein nrt1 ptr family	GO:0016020; GO:0042936
Solyc01g087820.2	Subtilisin-like protease (AHRD V1 **-- O82777_SOLLC); Interpro domain(s) IPR015500 Peptidase S8, subtilisin-related IPR000209 Peptidase S8 and S53, subtilisin, kexin, sedolisin	-2.11	3.34E-19	Subtilisin-like protease	GO:0004252; GO:0042802; GO:0043086
Solyc03g025670.2	PAR-1c protein (AHRD V1 ***- Q43589_TOBAC); Interpro domain(s) IPR009489 PAR1	-2.1	5.24E-18	Unknown Protein	
Solyc10g044680.1	Myb-like transcription factor (AHRD V1 **-- contains Interpro domain(s) IPR015495 Myb transcription factor	-2.09	4.37E-04	Transcription factor myb86	GO:0003677
Solyc01g087840.2	Subtilisin-like protease (AHRD V1 **-- O82006_SOLLC); Interpro domain(s) IPR015500 Peptidase S8, subtilisin-related	-2.08	3.83E-09	Subtilisin-like protease	GO:0004252; GO:0042802; GO:0043086
Solyc10g075150.1	Non-specific lipid-transfer protein (AHRD V1 **** contains Interpro domain(s) IPR013770 Plant lipid transfer protein and hydrophobic protein, helical	-2.08	2.09E-23	Non-specific lipid-transfer protein 2	GO:0006869
Solyc01g081170.1	Beta-glucosidase (AHRD V1 ***- B5U9B3_9BIVA); contains domain(s) IPR001360 Glycoside hydrolase, family 1	-2.07	2.69E-04	Beta-glucosidase 11	GO:0003824; GO:0005975; GO:0043169
Solyc03g119390.2	Transcription factor (AHRD V1 *-** D6MKM4_9ASPA); Interpro domain(s) IPR011598 Helix-loop-helix DNA-binding	-2.01	2.08E-04	Transcription factor bee 1	GO:0005634; GO:0030528; GO:0045449
Solyc04g040180.2	S-adenosylmethionine-dependent methyltransferase (Fragment) (AHRD V1 **** contains Interpro domain(s) IPR013217 Methyltransferase type 12	-2.01	3.00E-04	Methyltransferase	GO:0008757
Solyc11g005480.1	Citrate binding protein (AHRD V1 ***- contains Interpro domain(s) IPR014895 Alginate lyase 2	-2	2.23E-03	Citrate-binding	
Solyc01g008620.2	Beta-1 3-glucanase (AHRD V1 ***- Q9SYX6_TOBAC); Interpro domain(s) IPR000490 Glycoside hydrolase, family 17	-1.98	1.25E-14	3)-beta-glucan endohydrolase short	GO:0003824; GO:0005975; GO:0043169
Solyc03g120110.2	Serine/threonine kinase receptor (AHRD V1 **** contains Interpro domain(s) IPR002290 Serine/threonine protein kinase	-1.98	2.03E-06	G-type lectin s-receptor-like serine threonine-protein kinase ces101	GO:0004672; GO:0005524; GO:0006468

Solyc08g006470.2	Zinc finger family protein (AHRD V1 D7MB67_ARALY); contains Interpro domain(s) IPR007087 Zinc finger, C2H2-type	-1.98	6.07E-06	Zinc finger protein 622	GO:0005622; GO:0008270
Solyc10g055820.1	Chitinase (AHRD V1 ***- B9VRK7_CAPAN); contains domain(s) IPR000726 Glycoside hydrolase, family 19, catalytic	-1.98	3.85E-05	Endochitinase 3	GO:0004568; GO:0006032; GO:0016998
Solyc01g081160.2	Beta-glucosidase (AHRD V1 **** D3YJ59_VITVI); contains domain(s) IPR001360 Glycoside hydrolase, family 1	-1.97	9.29E-04	Beta-glucosidase 11-like	GO:0003824; GO:0005975; GO:0043169
Solyc04g050620.2	Cytochrome P450	-1.97	3.37E-07	Cytochrome p450 cyp736a12	
Solyc11g032220.1	NADPH dehydrogenase 1 (AHRD V1 **-* contains Interpro domain(s) IPR001155 NADH:flavin oxidoreductase/NADH oxidase, N-terminal	-1.97	7.62E-10	12-oxophytodienoate reductase 11	GO:0010181; GO:0016491
Solyc06g008620.1	Protein tolB (AHRD V1 *-*- D3SF14_THISK); Interpro domain(s) IPR011042 Six-bladed beta-propeller, TolB-like	-1.95	7.59E-11	Protein tolB	
Solyc02g079710.2	Serine/threonine kinase receptor (AHRD V1 **** contains Interpro domain(s) IPR002290 Serine/threonine protein kinase	-1.93	4.36E-04	G-type lectin s-receptor-like serine threonine-protein kinase	GO:0048544
Solyc04g007980.2	1-aminocyclopropane-1-carboxylate oxidase (AHRD V1 **-* B6SUN2_MAIZE); Interpro domain(s) IPR005123 Oxoglutarate and iron-dependent oxygenase	-1.93	2.18E-15	1-aminocyclopropane-1-carboxylate oxidase homolog 4	GO:0016491
Solyc09g010160.1	NAC domain protein IPR003441 (AHRD V1 B9IIS2_POPTR); contains Interpro domain(s) IPR003441 No apical meristem (NAM) protein	-1.92	4.46E-06	Nac domain-containing protein 90	GO:0003677; GO:0045449
Solyc03g020010.1	Kunitz-type trypsin inhibitor alpha chain (AHRD **** ID5A_PROJU); contains Interpro domain(s) IPR002160 Proteinase inhibitor I3, Kunitz legume	-1.91	1.56E-03	Miraculin	GO:0004866; GO:0004867; GO:0015066
Solyc07g008210.2	TPR domain protein (AHRD V1 *-*- contains Interpro domain(s) IPR011990 Tetratricopeptide-like helical	-1.91	5.78E-09	Tetratricopeptide repeat-superfamily	GO:0005488
Solyc08g005510.1	Tir-nbs-lrr, resistance protein	-1.89	5.15E-05	Tmv resistance protein	GO:0004888; GO:0007165; GO:0031224; GO:0045087
Solyc09g090970.2	Major allergen Mal d 1 (AHRD ***- Q84LA7_MALDO); contains Interpro domain(s) IPR000916 Bet v I allergen	-1.89	2.43E-04	Pathogenesis-related protein sth-2	GO:0006952; GO:0009607
Solyc05g053600.2	ATP-binding cassette transporter (AHRD V1 ***- contains Interpro domain(s) IPR013525 ABC-2 type transporter	-1.88	8.93E-09	Pleiotropic drug resistance protein 1	GO:0016020
Solyc03g098480.1	Unknown Protein (AHRD V1)	-1.85	2.74E-06	Unknown Protein	
Solyc05g008250.1	Myb-like transcription factor 6 (AHRD V1 Q8H256_GOSHI); contains Interpro domain(s) IPR015495 Myb transcription factor	-1.83	3.48E-08	Transcription factor myb3	GO:0003700; GO:0045449
Solyc05g009040.2	Receptor-like protein kinase (AHRD V1 ***- contains Interpro domain(s) IPR002290 Serine/threonine protein kinase	-1.83	2.79E-04	Probable receptor-like protein kinase	GO:0004672; GO:0005524; GO:0006468
Solyc10g075090.1	Non-specific lipid-transfer protein (AHRD V1 **** contains Interpro domain(s) IPR013770 Plant lipid transfer protein and hydrophobic protein, helical	-1.83	5.89E-19	Non-specific lipid-transfer protein 2	GO:0006869
Solyc12g011150.1	Unknown Protein (AHRD V1)	-1.82	5.28E-08	Unknown Protein	
Solyc02g068170.1	Unknown Protein (AHRD V1)	-1.81	6.04E-05	Unknown Protein	
Solyc03g118850.2	Hydrolase alpha/beta fold family (AHRD V1 Q1DF33_MYXXD)	-1.81	5.06E-04	Unknown Protein	GO:0004252; GO:0006508
Solyc10g085880.1	UDP-glucosyltransferase family 1 protein (AHRD V1 C6KI43_CITSI); contains Interpro domain(s) IPR002213 UDP-glucuronosyl/UDP-glucosyltransferase	-1.81	3.93E-06	Udp-glycosyltransferase 73c3	GO:0008152; GO:0016758
Solyc01g067460.1	Glutaredoxin family protein (AHRD V1 ***- contains Interpro domain(s) IPR011905 Glutaredoxin-like, plant II	-1.8	6.90E-13	Monothiol glutaredoxin-s2	GO:0045454
Solyc02g072550.1	DOG1 alpha splice variant (Fragment) (AHRD ***- A0SVJ8_ARATH)	-1.8	1.39E-03	Transcription factor hbp-1b	
Solyc07g055560.2	G-type lectin S-receptor-like serine/threonine-protein kinase At2g19130 V1 *--- Y2913_ARATH); contains Interpro domain(s) IPR002401 Cytochrome P450, E-class, group I	-1.8	4.52E-07	Cytochrome p450 cyp72a219	GO:0004672; GO:0005524; GO:0006468
Solyc01g100490.2	Nicotianamine synthase (AHRD V1 ***- A3DUI9_MALXI); Interpro domain(s) IPR004298 Nicotianamine synthase	-1.79	8.74E-07	Nicotianamine synthase	GO:0030410; GO:0030418
Solyc07g062480.1	S-locus glycoprotein (Fragment) (AHRD V1 *--- contains Interpro domain(s) IPR001480 Curculin-like (mannose-binding) lectin	-1.79	3.96E-05	Epidermis-specific secreted glycoprotein ep1	GO:0005529
Solyc07g055840.2	Citrate synthase (AHRD V1 **** B9IFM7_POPTR); Interpro domain(s) IPR010953 Citrate synthase, type II	-1.78	4.66E-07	Citrate glyoxysomal	GO:0044262; GO:0046912
Solyc08g078760.1	AT5g47580/MNJ7_17 (AHRD V1 ***- Q9FGJ9_ARATH)	-1.78	3.19E-11	Unknown Protein	

Solyc07g053890.2	O-acyltransferase WSD1 (AHRD V1 **-- WSD1_ARATH); Interpro domain(s) IPR009721 Protein of unknown function DUF1298	-1.76	2.18E-09	O-acyltransferase wsd1	
Solyc01g107780.2	UDP-glucosyltransferase family 1 protein (AHRD V1 C6KI43_CITSI); contains Interpro domain(s) IPR002213 UDP-glucuronosyl/UDP-glucosyltransferase	-1.75	3.78E-08	Scopoletin glucosyltransferase	GO:0008152; GO:0016758
Solyc06g050790.2	Amino acid transporter (AHRD V1 **** contains Interpro domain(s) IPR013057 Amino acid transporter, transmembrane	-1.75	2.39E-05	Sodium-coupled neutral amino acid transporter 7	GO:0015171
Solyc10g055800.1	Chitinase (AHRD V1 ***- B9VRK7_CAPAN); contains domain(s) IPR016283 Glycoside hydrolase, family 19	-1.75	4.50E-11	Endochitinase 4	GO:0008061
Solyc03g121010.2	Unknown Protein (AHRD V1)	-1.74	7.10E-04	Unknown Protein	
Solyc04g005810.2	Thioredoxin H (AHRD V1 ***- Q4U0W0_NICAL); Interpro domain(s) IPR015467 Thioredoxin, core	-1.74	9.91E-05	Thioredoxin h2	GO:0045454
Solyc08g079900.1	Subtilisin-like protease (AHRD V1 **-- Q9LWA4_SOLLC); Interpro domain(s) IPR015500 Peptidase S8, subtilisin-related	-1.74	1.99E-07	Subtilisin-like protease	GO:0004252; GO:0006508
Solyc03g033840.2	26S protease regulatory subunit 6B homolog V1 *--- PRS6B_ASPNG); contains Interpro domain(s) IPR003959 ATPase, AAA-type, core	-1.73	8.35E-08	Probable mitochondrial chaperone bcs1-a	GO:0005524
Solyc04g079030.2	Anthocyanidin 3-O-glucosyltransferase (AHRD V1 *-*- B6TRK5_MAIZE); Interpro domain(s) IPR002213 UDP-glucuronosyl/UDP-glucosyltransferase	-1.73	3.61E-05	Udp-glycosyltransferase 79b6	GO:0008152; GO:0016758
Solyc11g065940.1	Epsin 2-like protein (Fragment) (AHRD V1 D9HX40_LAGLG); contains Interpro domain(s) IPR001026 Epsin, N-terminal	-1.73	1.15E-15	Enth vhs family protein	GO:0005488
Solyc01g106820.2	Peptidase M50 family (AHRD V1 ***-	-1.72	9.42E-07	Probable zinc metallopeptidase chloroplastic	
Solyc04g079470.2	Serpin (Serine protease inhibitor) (AHRD V1 Q53P13_ORYSJ); contains Interpro domain(s) IPR015554 Protease inhibitor I4, serpin, plant	-1.72	2.38E-03	Serpin-zx	GO:0004867
Solyc10g080670.1	Unknown Protein (AHRD V1)	-1.72	9.50E-12	Unknown Protein	
Solyc02g077040.2	Cathepsin B-like cysteine proteinase 5 (AHRD **-* CPR5_CAEEL); contains Interpro domain(s) IPR013128 Peptidase C1A, papain	-1.71	4.67E-14	Senescence-specific cysteine protease sag39	GO:0004197; GO:0006508
Solyc10g055810.1	Endochitinase (Chitinase) (AHRD V1 **** Q43184_SOLTU); Interpro domain(s) IPR016283 Glycoside hydrolase, family 19 IPR018371 Chitin-binding, type 1, conserved site IPR000726	-1.71	8.38E-14	Endochitinase 4	GO:0004568; GO:0006032; GO:0016998
Solyc01g006550.2	LRR receptor-like serine/threonine-protein kinase, RLP	-1.7	3.94E-09	Receptor-like protein 12	GO:0005515
Solyc08g007430.1	Nitrate transporter (AHRD V1 **** Q852P5_TOBAC); Interpro domain(s) IPR000109 TGF-beta receptor, type I/II extracellular region	-1.7	2.43E-03	Protein nrt1 ptr family	GO:0016020; GO:0042936
Solyc02g036480.1	Harpin-induced protein-like (Fragment) (AHRD V1 **-- contains Interpro domain(s) IPR010847 Harpin-induced 1	-1.69	1.79E-03	Protein yls9	
Solyc05g052280.2	Peroxidase (AHRD V1 ***- B9VRK9_CAPAN); contains domain(s) IPR002016 Haem peroxidase, plant/fungal/bacterial	-1.69	1.69E-06	Peroxidase p7	GO:0004601; GO:0006979; GO:0020037; GO:0055114
Solyc11g044440.1	Serine/threonine-protein phosphatase 7 long form homolog V1 **-- PPP7L_ARATH); contains Interpro domain(s) IPR019557 Aminotransferase-like, plant mobile domain	-1.69	6.71E-05	Serine threonine-protein phosphatase 7 long form homolog	
Solyc12g042480.1	Cytochrome P450	-1.69	5.22E-10	Cytochrome p450	
Solyc01g010480.2	Potassium voltage-gated channel subfamily H member (AHRD V1 *--- KCNH8_HUMAN); contains Interpro domain(s) IPR013767 PAS fold	-1.67	7.29E-11	Protein twin lov 1	GO:0000155; GO:0000160
Solyc08g005890.2	Hydroxycinnamoyl transferase (AHRD V1 **-* D2XJ64_9MAGN); Interpro domain(s) IPR003480 Transferase	-1.66	2.97E-04	Uncharacterized acetyltransferase	GO:0016747
Solyc09g057960.1	Cysteine-rich receptor-like protein kinase (AHRD V1 C6ZRS1_SOYBN); contains Interpro domain(s) IPR002902 Protein of unknown function DUF26	-1.66	2.52E-04	Cysteine-rich repeat secretory protein 55	
Solyc01g094140.2	Cytochrome P450	-1.64	1.03E-03	Cytochrome p450 704c1	GO:0004497; GO:0005506; GO:0020037; GO:0055114
Solyc01g097270.2	Chitinase (Fragment) (AHRD V1 *--- Q38777_ALLSA); Interpro domain(s) IPR000726 Glycoside hydrolase, family 19, catalytic IPR001153 Barwin	-1.64	7.89E-12	Wound-induced protein win2	GO:0008061
Solyc04g080650.2	Cytochrome P450	-1.64	7.72E-04	Abscisic acid 8 -hydroxylase 2	GO:0004497; GO:0005506; GO:0020037
Solyc01g098590.2	Phosphoglycerate mutase family protein (AHRD V1 C1DZH2_9CHLO); contains Interpro domain(s) IPR013078 Phosphoglycerate mutase	-1.63	1.40E-12	Broad-range acid phosphatase det1	GO:0003824; GO:0008152

Solyc00g282510.1	Phenylalanine ammonia-lyase (AHRD V1 ***- B5LAW0_CAPAN); Interpro domain(s) IPR005922 Phenylalanine ammonia-lyase	-1.62	5.16E-05	Unknown Protein	GO:0005737; GO:0006559; GO:0016841
Solyc00g201160.2	Receptor protein kinase (AHRD V1 **** contains Interpro domain(s) IPR002290 Serine/threonine protein kinase	-1.61	1.84E-03	Unknown Protein	GO:0004674; GO:0006468
Solyc10g080230.1	DNA binding protein-like (AHRD V1 ***- contains Interpro domain(s) IPR005516 Remorin, C-terminal region	-1.61	1.72E-05	Gata zinc finger domain-containing protein 8	
Solyc01g009690.1	LRR receptor-like serine/threonine-protein kinase, RLP	-1.59	1.95E-05	Receptor-like protein 12	GO:0005515
Solyc01g096320.2	Homeobox leucine zipper protein (AHRD V1 Q9XH73_PRUAR); contains Interpro domain(s) IPR001356 Homeobox	-1.59	1.66E-05	Homeobox-leucine zipper protein athb-12	GO:0003677; GO:0045449
Solyc02g068830.1	Receptor like kinase, RLK	-1.59	1.38E-07	Probable lrr receptor-like serine threonine-protein kinase	GO:0004672; GO:0005524; GO:0006468
Solyc06g054570.1	Glutaredoxin family protein (AHRD V1 ***- contains Interpro domain(s) IPR011905 Glutaredoxin-like, plant II	-1.59	7.18E-04	Monothiol glutaredoxin-s4	GO:0045454
Solyc06g069070.1	Lipid transfer protein (AHRD V1 ***- contains Interpro domain(s) IPR013770 Plant lipid transfer protein and hydrophobic protein, helical	-1.58	2.57E-08	Non-specific lipid-transfer protein 2	GO:0006869
Solyc07g043250.1	Unknown Protein (AHRD V1); contains Interpro IPR008889 VQ	-1.58	5.61E-05	Unknown Protein	
Solyc08g077370.2	Purine permease family protein (AHRD V1 D7MCV2_ARALY); contains Interpro domain(s) IPR004853 Protein of unknown function DUF250	-1.58	1.03E-03	Probable purine permease 11	GO:0005345
Solyc10g075070.1	Non-specific lipid-transfer protein (AHRD V1 ***- contains Interpro domain(s) IPR013770 Plant lipid transfer protein and hydrophobic protein, helical	-1.58	5.20E-10	Non-specific lipid-transfer protein 2	GO:0006869
Solyc02g089490.2	Unknown Protein (AHRD V1)	-1.57	1.82E-03	Unknown Protein	
Solyc12g089130.1	CRAL/TRIO domain containing protein expressed (AHRD **-- Q75GU9_ORYSJ); contains Interpro domain(s) IPR001251 Cellular retinaldehyde-binding/triple function, C-terminal	-1.55	2.17E-04	Random slug protein 5	
Solyc07g042520.2	Sucrose synthase 4 (AHRD V1 **** contains Interpro domain(s) IPR012820 Sucrose synthase, plants and cyanobacteria IPR000368 Sucrose synthase	-1.54	6.64E-06	Sucrose synthase	GO:0009058
Solyc07g062500.2	Cytochrome P450	-1.54	9.69E-06	Cytochrome p450 cyp72a219	GO:0004497; GO:0005506; GO:0020037
Solyc10g080240.1	Remorin family protein (AHRD V1 ***- contains Interpro domain(s) IPR005516 Remorin, C-terminal region	-1.54	9.37E-04	Gata zinc finger domain-containing protein 8	
Solyc02g063410.2	Hydroxyproline-rich glycoprotein (AHRD V1 ***- A9YWR1_MEDTR)	-1.53	1.56E-03	Unknown Protein	
Solyc06g084820.1	cytochrome P450	-1.52	7.12E-07	Geraniol 8-hydroxylase-	GO:0020037; GO:0005506; GO:0004497; GO:0055114
Solyc07g056200.2	NBS-LRR class disease resistance protein (AHRD **-- D5L9G6_ORYSI)	-1.52	6.05E-05	Metal ion binding	
Solyc08g079870.1	Subtilisin-like protease (AHRD V1 **-- Q9LWA3_SOLLC); Interpro domain(s) IPR015500 Peptidase S8, subtilisin-related IPR000209 Peptidase S8 and S53, subtilisin, kexin, sedolisin	-1.52	1.16E-12	Subtilisin-like protease	GO:0004252; GO:0042802; GO:0043086
Solyc11g011710.1	Auxin-responsive protein (AHRD V1 ***- A8IXW9_BRACM); Interpro domain(s) IPR003676 Auxin responsive SAUR protein	-1.52	7.53E-05	Auxin-induced protein 6b	
Solyc06g054090.1	Ribosomal-protein-alanine N-acetyltransferase (AHRD V1 **** D3FS71_BACPE); Interpro domain(s) IPR000182 GCN5-related N-acetyltransferase	-1.51	2.46E-04	Gcn5-related n-acetyltransferase family protein	GO:0008152; GO:0008999
Solyc06g060590.2	Nodulin MtN3 family protein (AHRD V1 D7KK13_ARALY); contains Interpro domain(s) IPR018179 RAG1-activating protein 1 homologue	-1.5	1.34E-07	Bidirectional sugar transporter sweet1	GO:0016021
Solyc11g045460.1	CXE carboxylesterase (AHRD V1 **-- Q0ZPW1_9ROSA); Interpro domain(s) IPR013094 Alpha/beta hydrolase fold-3	-1.5	1.70E-03	Probable carboxylesterase 15	GO:0008152; GO:0016787
Solyc03g120260.2	Coatomer beta&apos subunit (AHRD V1 ***- contains Interpro domain(s) IPR020472 G-protein beta WD-40 repeat, region	-1.49	2.04E-04	Beta subunit isoform 1	
Solyc01g097240.2	Pathogenesis-related protein 4B (Fragment) (AHRD V1 Q6LBM4_TOBAC); contains Interpro domain(s) IPR018226 Barwin, conserved site IPR001153 Barwin	-1.48	1.22E-06	Chitinase hevein pr-4 wheatwin2	GO:0004568; GO:0006032; GO:0016998
Solyc07g064600.2	Endoribonuclease L-PSP family protein (AHRD V1 D7LAZ0_ARALY); contains Interpro domain(s) IPR019897 YjgF-like protein, conserved site	-1.48	2.01E-03	Ribonuclease uk114	GO:0005515; GO:0004521
Solyc11g066590.1	Lysosomal Pro-X carboxypeptidase (AHRD V1 **-- contains Interpro domain(s) IPR008758 Peptidase S28	-1.48	6.22E-09	Lysosomal pro-x carboxypeptidase-	GO:0006508; GO:0008236

Solyc02g070020.1	UDP-glucosyltransferase (AHRD V1 **-* Q8LKG3_STERE); contains domain(s) IPR002213 UDP-glucuronosyl/UDP-glucosyltransferase	-1.47	6.56E-05	Udp-glycosyltransferase 91c1	GO:0008152; GO:0016758
Solyc12g088190.1	Amino acid permease 6 (AHRD V1 Q7Y076_BRANA); contains Interpro domain(s) IPR013057 Amino acid transporter, transmembrane	-1.46	3.87E-09	Amino acid permease 6	GO:0015175; GO:0015172
Solyc07g017610.2	Saccharopine dehydrogenase (NAD(+) L-glutamate-forming) (AHRD V1 C7PUW7_CHIPD); contains Interpro domain(s) IPR005097 Saccharopine dehydrogenase	-1.45	1.45E-08	Alpha-aminoadipic semialdehyde synthase	GO:0047131; GO:0055114
Solyc10g083690.2	Cytochrome P450	-1.45	1.17E-04	Cytochrome p450 76a2	GO:0004497; GO:0005506; GO:0020037
Solyc04g064530.1	Unknown Protein (AHRD V1)	-1.44	1.08E-08	Unknown Protein	
Solyc08g079430.2	Primary amine oxidase (AHRD V1 ***- contains Interpro domain(s) IPR000269 Copper amine oxidase	-1.44	1.40E-09	Primary amine oxidase	GO:0005507; GO:0008131; GO:0009308; GO:0048038; GO:0055114
Solyc09g097770.2	Cell wall protein (AHRD V1 ***- contains Interpro domain(s) IPR010800 Glycine rich	-1.44	2.22E-05	Tyrosine- and lysine-rich protein precursor	
Solyc01g067020.2	Receptor like kinase, RLK	-1.42	6.15E-08	Probable inactive receptor kinase	GO:0004675; GO:0006468
Solyc02g079870.2	Unknown Protein (AHRD V1)	-1.42	4.96E-08	Unknown Protein	
Solyc04g009850.2	1-aminocyclopropane-1-carboxylate oxidase-like protein (AHRD V1 **-* contains Interpro domain(s) IPR005123 Oxoglutarate and iron-dependent oxygenase	-1.42	6.00E-05	1-aminocyclopropane-1-carboxylate oxidase homolog 1	GO:0016491
Solyc06g050800.2	Amino acid transporter (AHRD V1 **** contains Interpro domain(s) IPR013057 Amino acid transporter, transmembrane	-1.42	3.63E-06	Probable sodium-coupled neutral amino acid transporter 6	GO:0015171
Solyc02g076980.2	Cathepsin B-like cysteine proteinase (AHRD V1 CYSP_SCHMA); contains Interpro domain(s) IPR013128 Peptidase C1A, papain IPR000169 Peptidase, cysteine peptidase active site	-1.41	1.91E-08	Cysteine protease	GO:0004197; GO:0006508
Solyc03g083420.2	OBP3-responsive gene 1 (AHRD V1 **-- contains Interpro domain(s) IPR002290 Serine/threonine protein kinase	-1.4	1.09E-03	Probable plastid-lipid-associated protein chloroplastic	GO:0005515; GO:0006468
Solyc04g005610.2	NAC domain transcription factor (AHRD V1 Q5DM36_WHEAT); contains Interpro domain(s) IPR003441 No apical meristem (NAM) protein	-1.4	3.59E-04	Nac transcription factor 29	GO:0003677; GO:0045449
Solyc04g072380.2	Phosphatidylinositol 3,4,5-trisphosphate-dependent Rac exchanger 2 protein V1 *--- PREX2_MOUSE); contains Interpro domain(s) IPR006869 Protein of unknown function DUF547	-1.4	6.45E-04	Vacuolar membrane-associated iml1	GO:0007242
Solyc03g095650.2	MLO-like protein 17 (AHRD V1 ***- contains Interpro domain(s) IPR004326 Mlo-related protein	-1.39	1.09E-12	Mlo1	GO:0008219; GO:0016021
Solyc05g006990.2	Nitrate transporter (AHRD V1 **-* Q7XAK5_PRUPE); Interpro domain(s) IPR000109 TGF-beta receptor, type I/II extracellular region	-1.39	3.21E-07	Protein nrt1 ptr family	GO:0016020; GO:0042936
Solyc01g006290.2	Peroxidase (AHRD V1 **** Q42964_TOBAC); contains domain(s) IPR002016 Haem peroxidase, plant/fungal/bacterial	-1.38	1.47E-07	Lignin-forming anionic peroxidase	GO:0004601; GO:0006979; GO:0020037; GO:0055114
Solyc04g079420.2	Nbs-lrr, resistance protein	-1.38	1.37E-09	Probable disease resistance protein	GO:0006952
Solyc06g076330.2	Laccase (AHRD V1 **** O24044_LIRTU); contains domain(s) IPR017761 Laccase	-1.38	1.06E-03	Laccase 2	GO:0016491
Solyc11g017280.1	Receptor like kinase, RLK	-1.38	2.93E-04	Leucine-rich repeat receptor-like tyrosine-protein kinase	GO:0006468
Solyc01g005470.2	Cell number regulator 10 (AHRD V1 D9HP26_MAIZE); contains Interpro domain(s) IPR006461 Protein of unknown function Cys-rich	-1.37	3.17E-05	Protein plant cadmium resistance 2	
Solyc04g053110.1	Glutaredoxin (AHRD V1 **** B9I9V9_POPTR); contains domain(s) IPR011905 Glutaredoxin-like, plant II	-1.37	1.05E-03	Monothiol glutaredoxin-s6-like	GO:0045454; GO:0008794
Solyc06g048410.2	Superoxide dismutase (AHRD V1 **** Q7YK44_SOLLC); Interpro domain(s) IPR019833 Manganese/iron superoxide dismutase, binding site IPR001189 Manganese/iron superoxide dismutase	-1.37	3.96E-08	Superoxide dismutase	GO:0004784; GO:0006801; GO:0046872; GO:0055114
Solyc06g069430.2	MADS box transcription factor (AHRD V1 Q9SBQ1_PETHY); contains Interpro domain(s) IPR002487 Transcription factor, K-box	-1.37	3.39E-05	Agamous-like mads-box protein agl8 homolog	GO:0003700; GO:0005634; GO:0006355
Solyc09g064940.2	Phenazine biosynthesis protein PhzF family (AHRD ***- B4VKV9_9CYAN); contains Interpro domain(s) IPR003719 Phenazine biosynthesis PhzC/PhzF protein	-1.37	3.04E-10	Phenazine biosynthesis-like domain-containing protein 1	GO:0003824; GO:0009058

Solyc01g005230.2	S-adenosyl-L-methionine salicylic acid carboxyl methyltransferase (AHRD **** A7XZE9_9MAGN); contains Interpro domain(s) IPR005299 SAM dependent carboxyl methyltransferase	-1.36	8.82E-04	Probable s-adenosylmethionine-dependent methyltransferase	GO:0008168
Solyc01g010770.2	Spfh domain / band 7 family (AHRD V1 **-- B4AS86_FRANO); contains Interpro domain(s) IPR001107 Band 7 protein	-1.36	1.36E-06	Hypersensitive-induced response protein 2	GO:0005515
Solyc07g005420.1	Unknown Protein (AHRD V1)	-1.36	9.62E-05	Unknown Protein	
Solyc07g008310.2	Rieske (2Fe-2S) domain protein (AHRD V1 Q024N8_SOLUE); contains Interpro domain(s) IPR001663 Aromatic-ring-hydroxylating dioxygenase, alpha subunit	-1.35	6.03E-08	Choline chloroplastic	GO:0005506; GO:0006725; GO:0051537; GO:0055114
Solyc05g050340.2	WRKY transcription factor 6 (AHRD V1 C9DHZ5_9ROSI); contains Interpro domain(s) IPR003657 DNA-binding WRKY	-1.34	1.01E-04	Probable wrky transcription factor 41	GO:0003700; GO:0005634; GO:0043565; GO:0045449
Solyc12g100030.1	LRR receptor-like serine/threonine-protein kinase, RLP	-1.34	1.05E-04	Receptor-like protein 12	GO:0005515; GO:0004675
Solyc01g102850.1	Tir-nbs-lrr, resistance protein	-1.33	5.72E-08	Tmv resistance protein n	GO:0004888; GO:0007165; GO:0031224; GO:0045087
Solyc12g006380.1	1-aminocyclopropane-1-carboxylate oxidase-like protein (AHRD V1 **-* contains Interpro domain(s) IPR005123 Oxoglutarate and iron-dependent oxygenase	-1.33	5.64E-06	1-aminocyclopropane-1-carboxylate oxidase homolog	GO:0016491
Solyc02g063510.1	Unknown Protein (AHRD V1)	-1.32	1.99E-03	Unknown Protein	
Solyc03g026370.1	Peptidoglycan-binding LysM domain-containing protein (AHRD V1 D7MLA8_ARALY); contains Interpro domain(s) IPR018392 Peptidoglycan-binding lysin domain	-1.32	1.34E-03	Peptidoglycan-binding domain-containing protein	GO:0016998
Solyc05g054750.2	Plant-specific domain TIGR01589 family protein (AHRD *-*- B4FFL2_MAIZE); contains Interpro domain(s) IPR006476 Conserved hypothetical protein CHP01589, plant	-1.32	3.00E-06	Unknown Protein	
Solyc09g083200.2	Nod factor receptor protein (Fragment) (AHRD ***- B6ZN07_GLYSO); contains Interpro domain(s) IPR002482 Peptidoglycan-binding Lysin subgroup	-1.32	4.62E-05	Protein lyk5-	GO:0016998
Solyc01g006300.2	Peroxidase (AHRD V1 **** Q43774_SOLLC); contains domain(s) IPR002016 Haem peroxidase, plant/fungal/bacterial	-1.31	2.11E-08	Lignin-forming anionic peroxidase	GO:0004601; GO:0006979; GO:0020037; GO:0055114
Solyc02g063020.1	Major facilitator superfamily MFS_1 (AHRD V1 A4FJQ9_SACEN); contains Interpro domain(s) IPR016196 Major facilitator superfamily, general substrate transporter	-1.31	3.39E-04	Major facilitator superfamily protein	GO:0055085; GO:0005351
Solyc05g007770.2	NAC domain transcription factor (AHRD V1 Q5DM36_WHEAT); contains Interpro domain(s) IPR003441 No apical meristem (NAM) protein	-1.31	2.98E-05	Nac transcription factor 29-like	GO:0003677; GO:0045449
Solyc12g045020.1	Cytochrome P450	-1.31	2.39E-04	Cytochrome p450 cyp736a12	GO:0004497; GO:0005506; GO:0020037
Solyc01g095170.2	Harpin-induced protein (AHRD V1 ***- B6SP51_MAIZE); Interpro domain(s) IPR010847 Harpin-induced 1	-1.3	7.10E-04	Late embryogenesis abundant hydroxyproline-rich glycoprotein	
Solyc03g082620.2	Oligopeptide transporter (Fragment) (AHRD V1 **** contains Interpro domain(s) IPR004813 Oligopeptide transporter OPT superfamily	-1.3	1.23E-06	Metal-nicotianamine transporter ysl2	GO:0015198; GO:0055085
Solyc04g074000.2	Receptor like kinase, RLK	-1.3	4.58E-07	Probable lrr receptor-like serine threonine-protein kinase	GO:0004713; GO:0005524; GO:0006468
Solyc07g008240.2	Non-symbiotic hemoglobin protein (AHRD V1 ***- contains Interpro domain(s) IPR001032 Leghaemoglobin	-1.3	1.47E-03	Non-symbiotic hemoglobin 1	GO:0005506; GO:0015671; GO:0019825; GO:0020037
Solyc08g016210.2	LRR receptor-like serine/threonine-protein kinase, RLP	-1.3	2.81E-07	Lrr receptor-like serine threonine-protein kinase	GO:0005515
Solyc01g080410.2	Peptide methionine sulfoxide reductase msrB (AHRD ***- B6T2C6_MAIZE); contains Interpro domain(s) IPR002579 Methionine sulphoxide reductase B	-1.29	7.74E-04	Peptide methionine sulfoxide reductase b5	GO:0055114
Solyc02g084850.2	Unknown Protein (AHRD V1); contains Interpro IPR000167 Dehydrin	-1.29	6.27E-05	Unknown Protein	GO:0006950; GO:0009415
Solyc03g078150.2	Amino acid transporter family protein (AHRD **** D7LGK0_ARALY); contains Interpro domain(s) IPR013057 Amino acid transporter, transmembrane	-1.29	4.63E-04	Vacuolar amino acid transporter 1	GO:0015171
Solyc03g098240.2	Glutamate decarboxylase (AHRD V1 **** B6TV07_MAIZE); Interpro domain(s) IPR010107 Glutamate decarboxylase	-1.29	8.49E-06	Glutamate decarboxylase	GO:0003824; GO:0030170
Solyc04g007800.2	C2 domain-containing protein (AHRD V1 **-- contains Interpro domain(s) IPR018029 C2 membrane targeting protein	-1.29	1.58E-05	Probable adp-ribosylation factor gtpase-activating protein agd11	
Solyc04g040130.1	Omega-6 fatty acid desaturase (AHRD V1 Q461Q1_HEVBR); contains Interpro domain(s) IPR005804 Fatty acid desaturase, type 1	-1.29	2.72E-04	Omega-6 fatty acid endoplasmic reticulum isozyme 2	GO:0006629; GO:0016491

Solyc05g050130.2	Acidic chitinase (AHRD V1 **-- Q71HN4_FICAW); Interpro domain(s) IPR001223 Glycoside hydrolase, family 18, catalytic domain	-1.29	4.60E-07	Acidic endochitinase	GO:0003824; GO:0005975; GO:0043169
Solyc08g068860.2	Aspartic proteinase nepenthesin-1 (AHRD V1 **-- contains Interpro domain(s) IPR001461 Peptidase A1	-1.29	4.36E-09	Protein aspartic protease in guard cell 2	GO:0004190; GO:0006508
Solyc09g075210.2	Late embryogenesis abundant protein 5 (AHRD ***- Q1HGG0_9ROSI); contains Interpro domain(s) IPR004926 Late embryogenesis abundant protein 3	-1.29	3.35E-04	Late embryogenesis abundant protein lea5	GO:0006950
Solyc07g055710.2	Heat stress transcription factor A3 (AHRD *-*- D1M7W9_SOLLC); contains Interpro domain(s) IPR000232 Heat shock factor (HSF)-type, DNA-binding	-1.28	9.10E-04	Heat stress transcription factor a-4b	GO:0003700; GO:0005634; GO:0006355; GO:0043565
Solyc08g007460.2	Non-specific lipid-transfer protein (AHRD V1 -*** contains Interpro domain(s) IPR003612 Plant lipid transfer protein/seed storage/trypsin-alpha amylase inhibitor	-1.28	4.01E-04	Lipid transfer-like protein vas	GO:0008289
Solyc01g107820.2	UDP-glucosyltransferase family 1 protein (AHRD V1 C6KI43_CITSI); contains Interpro domain(s) IPR002213 UDP-glucuronosyl/UDP-glucosyltransferase	-1.27	6.71E-07	Scopoletin glucosyltransferase	GO:0008152; GO:0016758
Solyc01g106620.2	Pathogenesis-related protein 1a (AHRD V1 **-- contains Interpro domain(s) IPR018244 Allergen V5/Tpx-1 related, conserved site	-1.26	5.64E-05	Basic form of pathogenesis-related protein 1	GO:0005515; GO:0005576
Solyc04g007990.1	Unknown Protein (AHRD V1)	-1.26	6.30E-05	Unknown Protein	
Solyc01g014840.2	Tir-nbs-lrr, resistance protein	-1.25	7.10E-04	Tmv resistance protein n	GO:0006952
Solyc04g072760.2	High affinity sulfate transporter 2 (AHRD **** SUT2_STYHA); contains Interpro domain(s) IPR001902 Sulphate anion transporter	-1.25	1.23E-03	Sulfate transporter	GO:0008271; GO:0008272; GO:0016020
Solyc05g050120.2	Malic enzyme (AHRD V1 ***- O04936_SOLLC); Interpro domain(s) IPR012302 Malic enzyme, NAD-binding	-1.25	1.17E-07	Nadp-dependent malic enzyme	GO:0003824; GO:0005488; GO:0008152
Solyc05g051850.2	Inositol-3-phosphate synthase (AHRD V1 ***- C4PW06_ARATH); Interpro domain(s) IPR002587 Myo-inositol-1-phosphate synthase	-1.24	8.09E-04	Inositol-3-phosphate synthase	GO:0003824; GO:0005488; GO:0008152
Solyc11g011440.1	Aspartic proteinase nepenthesin-1 (AHRD V1 **-- contains Interpro domain(s) IPR001461 Peptidase A1	-1.24	1.06E-05	Aspartic proteinase pcs1	GO:0004190; GO:0006508
Solyc01g081270.2	Glutathione S-transferase (AHRD V1 ***- Q8H9E5_CUCMA); Interpro domain(s) IPR004046 Glutathione S-transferase, C-terminal	-1.23	7.82E-04	Glutathione transferase gst 23	
Solyc04g014400.2	LRR receptor-like serine/threonine-protein kinase, RLP	-1.23	1.80E-09	Lrr receptor-like serine threonine-protein kinase fls2	GO:0005515
Solyc07g041900.2	Cathepsin L-like cysteine proteinase (AHRD V1 A7LJ78_DERVA); contains Interpro domain(s) IPR013128 Peptidase C1A, papain IPR000169 Peptidase, cysteine peptidase active site	-1.23	6.07E-04	Cysteine proteinase 3	GO:0004197; GO:0006508
Solyc05g008960.2	Receptor-like protein kinase (AHRD V1 ***- contains Interpro domain(s) IPR002290 Serine/threonine protein kinase	-1.22	5.74E-04	Probable serine threonine-protein kinase	GO:0006468
Solyc02g078380.2	Aluminum-induced protein-like protein (AHRD V1 **--	-1.21	2.40E-08	Stem-specific protein tsjt1	
Solyc01g108790.1	3-hydroxyisobutyryl-CoA hydrolase-like protein 2, mitochondrial (AHRD ***- HIBC5_ARATH)	-1.2	7.64E-04	3-hydroxyisobutyryl- hydrolase 1	
Solyc04g009860.2	1-aminocyclopropane-1-carboxylate oxidase-like protein (AHRD V1 **-* contains Interpro domain(s) IPR005123 Oxoglutarate and iron-dependent oxygenase	-1.2	1.39E-03	1-aminocyclopropane-1-carboxylate oxidase homolog 1	GO:0016491
Solyc12g013620.1	NAC domain protein IPR003441 (AHRD V1 B9N1K9_POPTR); contains Interpro domain(s) IPR003441 No apical meristem (NAM) protein	-1.2	6.68E-04	Nac domain-containing protein 72	GO:0003677; GO:0045449
Solyc06g071070.1	Short-chain dehydrogenase/reductase family protein (AHRD V1 D7L204_ARALY); contains Interpro domain(s) IPR002347 Glucose/ribitol dehydrogenase	-1.19	3.45E-04	Short-chain type dehydrogenase reductase	GO:0008152; GO:0016491
Solyc06g076470.2	Unknown Protein (AHRD V1)	-1.19	3.15E-05	Unknown Protein	
Solyc03g044790.2	Alpha-hydroxynitrile lyase (AHRD V1 **-- O49897_MANES); Interpro domain(s) IPR000073 Alpha/beta hydrolase fold-1	-1.18	1.66E-03	Salicylic acid-binding protein 2	GO:0003824; GO:0080030; GO:0080031
Solyc08g076050.2	ARK3 product/receptor-like serine/threonine protein kinase ARK3 V1 **** Q9S971_ARATH); contains Interpro domain(s) IPR002290 Serine/threonine protein kinase	-1.18	5.83E-05	G-type lectin s-receptor- serine threonine-protein kinase	GO:0004672; GO:0005524; GO:0006468
Solyc01g081310.2	Glutathione-S-transferase (AHRD V1 ***- B2BY02_9BRAS); contains domain(s) IPR004045 Glutathione S-transferase, N-terminal IPR017933 Glutathione S-transferase/chloride channel, C-terminal	-1.17	8.68E-05	Glutathione transferase gst 23	
Solyc02g084840.2	Dehydrin DHN1 (AHRD V1 *-*- DHN1_PEA); Interpro domain(s) IPR000167 Dehydrin	-1.17	2.72E-03	Cold shock protein	GO:0006950; GO:0009415

Solyc04g079480.2	Serpin (Serine protease inhibitor) (AHRD V1 Q53P13_ORYSJ); contains Interpro domain(s) IPR015554 Protease inhibitor I4, serpin, plant	-1.17	1.34E-03	Serpin-zx	GO:0004867
Solyc05g009500.2	Peptide transporter (AHRD V1 **-* A6YJX4_9MAGN); Interpro domain(s) IPR000109 TGF-beta receptor, type I/II extracellular region	-1.17	3.37E-08	Protein nrt1 ptr family	GO:0042937; GO:0016020; GO:0042936
Solyc07g007150.1	Unknown Protein (AHRD V1)	-1.17	6.73E-04	Unknown Protein	
Solyc08g068870.2	Aspartic proteinase nepenthesin-1 (AHRD V1 **-- contains Interpro domain(s) IPR001461 Peptidase A1	-1.17	1.12E-08	Protein aspartic protease in guard cell 2	GO:0004190; GO:0006508
Solyc04g006940.2	Phospholipid-transporting ATPase (AHRD V1 **** C1GDL1_PARBD); Interpro domain(s) IPR006539 ATPase, P-type, phospholipid-translocating, flippase	-1.16	1.44E-04	Phospholipid-transporting atpase 9	GO:0004012; GO:0016021
Solyc04g064590.1	Protein kinase (AHRD V1 *-*- Q40541_TOBAC); Interpro domain(s) IPR015748 Mitogen activated protein kinase kinase kinase 3	-1.16	6.03E-04	Mitogen-activated protein kinase kinase kinase yoda-like	GO:0006468
Solyc06g076520.1	class I heat shock protein (AHRD ***- Q69BI7_CARPA); contains Interpro domain(s) IPR002068 Heat shock protein Hsp20	-1.16	2.41E-05	Kda class i heat shock	GO:0051082
Solyc06g076560.1	class I heat shock protein (AHRD ***- Q69BI7_CARPA); contains Interpro domain(s) IPR002068 Heat shock protein Hsp20	-1.16	4.74E-06	Kda class i heat shock	GO:0051082
Solyc07g005100.2	Chitinase-like protein (AHRD V1 ***- O81861_ARATH); Interpro domain(s) IPR011583 Chitinase II	-1.16	3.32E-09	Class v	GO:0003824; GO:0005975; GO:0043169
Solyc01g105310.2	Metacaspase (AHRD V1 ***- A8NU42_COPC7); contains domain(s) IPR011600 Peptidase C14, caspase catalytic	-1.15	1.55E-04	Metacaspase-3	GO:0000287; GO:0004427; GO:0005737; GO:0006796
Solyc02g072470.2	Receptor like kinase, RLK	-1.14	6.22E-04	Probable lrr receptor-like serine threonine-protein kinase	GO:0005515; GO:0019199; GO:0055114
Solyc03g113270.2	Homeobox-leucine zipper-like protein (AHRD V1 *-*- contains Interpro domain(s) IPR001356 Homeobox IPR017970 Homeobox, conserved site	-1.14	3.67E-05	Homeobox-leucine zipper protein hat5	GO:0003677; GO:0045449
Solyc10g006750.2	CONSTANS-like zinc finger protein (AHRD V1 D0EP04_SOYBN); contains Interpro domain(s) IPR000315 Zinc finger, B-box	-1.14	6.33E-05	Zinc finger protein constans	GO:0005622; GO:0008270
Solyc10g085140.1	Undecaprenyl pyrophosphate synthase (AHRD V1 ***- contains Interpro domain(s) IPR001441 Di-trans-poly-cis-decaprenylcistransferase-like	-1.14	8.09E-06	Dehydrodolichyl diphosphate synthase 2	GO:0016765
Solyc07g043230.2	Low affinity zinc transporter (AHRD V1 A3LU47_PICST); contains Interpro domain(s) IPR004698 Zinc/iron permease, fungal and plant	-1.13	1.81E-05	Zinc transporter 5	GO:0016020; GO:0030001; GO:0046873
Solyc08g082120.2	Methanol inducible protein (AHRD V1 ***-	-1.13	9.73E-05	Methanol inducible protein	
Solyc09g008250.2	MYB transcription factor (AHRD V1 *--* contains Interpro domain(s) IPR015495 Myb transcription factor	-1.13	1.10E-04	Transcription factor rax2	GO:0003700; GO:0005516; GO:0003677
Solyc01g009700.1	LRR receptor-like serine/threonine-protein kinase, RLP	-1.12	7.67E-05	Receptor-like protein 12	GO:0005515
Solyc01g091870.2	Major facilitator superfamily domain-containing protein 8 V1 *--- MFSD8_XENLA); contains Interpro domain(s) IPR004331 SPX, N-terminal	-1.12	5.51E-04	Spx domain-containing membrane protein	GO:0055085
Solyc01g095320.2	BCL-2-associated athanogene 6 (AHRD V1 *-*- contains Interpro domain(s) IPR003103 Apoptosis regulator Bcl-2 protein, BAG	-1.12	2.16E-03	Bag family molecular chaperone regulator 6	GO:0006915
Solyc01g098020.2	Acetyltransferase GNAT family protein expressed (AHRD ***- Q75GC2_ORYSJ); contains Interpro domain(s) IPR000182 GCN5-related N-acetyltransferase	-1.12	4.76E-06	Unknown Protein	GO:0008152
Solyc01g108540.2	Acetyl esterase (AHRD V1 *--- AES_SHIFL); Interpro domain(s) IPR013094 Alpha/beta hydrolase fold-3	-1.12	1.51E-03	2-hydroxyisoflavanone dehydratase	GO:0008152; GO:0016787
Solyc03g031990.2	Auxin efflux carrier family protein (AHRD **** D7KJ74_ARALY); contains Interpro domain(s) IPR004776 Auxin efflux carrier	-1.12	1.81E-03	Uncharacterized transporter	GO:0016021
Solyc07g008020.2	Auxin response factor 16 (AHRD V1 ARFP_ORYSJ); contains Interpro domain(s) IPR003311 AUX/IAA protein	-1.12	1.99E-06	Auxin-responsive protein	GO:0005634; GO:0045449
Solyc09g090080.1	Inorganic phosphate transporter (AHRD V1 **** contains Interpro domain(s) IPR004738 Phosphate permease	-1.12	4.73E-04	Inorganic phosphate transporter 1-4	GO:0005315; GO:0016021
Solyc01g008510.2	Photosystem II 5 kDa protein, chloroplastic V1 *-*- PST2_GOSHI)	-1.11	2.63E-03	Photosystem ii 5 kda chloroplastic	
Solyc01g079170.2	Galactinol synthase (AHRD V1 ***- D6QSF5_BRANA); Interpro domain(s) IPR002495 Glycosyl transferase, family 8	-1.11	6.40E-05	Galactinol synthase 1	GO:0016757
Solyc01g109500.2	BURP domain-containing protein (AHRD V1 *--- contains Interpro domain(s) IPR004873 BURP	-1.11	1.60E-08	Burp domain-containing protein 3	

Solyc03g078370.1	Receptor-like protein kinase (AHRD V1 ***- contains Interpro domain(s) IPR002290 Serine/threonine protein kinase	-1.11	1.96E-03	G-type lectin s-receptor-like serine threonine-protein kinase rlk1	GO:0004672; GO:0005524; GO:0006468
Solyc05g015490.2	Non-specific lipid-transfer protein (AHRD V1 ***- contains Interpro domain(s) IPR003612 Plant lipid transfer protein/seed storage/trypsin-alpha amylase inhibitor	-1.11	5.20E-08	Non-specific lipid transfer protein gpi-anchored 1	
Solyc12g096570.1	ARGOS (AHRD V1 ***- C7SFP7_SOLLC)	-1.11	7.61E-05	Argos-like protein	
Solyc01g005410.2	Calcium binding protein Caleosin (AHRD V1 A8N0P5_COPC7); contains Interpro domain(s) IPR007736 Caleosin related	-1.1	9.21E-04	Probable peroxygenase 5	GO:0005509
Solyc01g096340.2	Auxin-induced SAUR-like protein (AHRD V1 ***- contains Interpro domain(s) IPR003676 Auxin responsive SAUR protein	-1.1	1.51E-05	Auxin-induced protein 15a	GO:0005516
Solyc02g085770.2	ABA induced plasma membrane protein PM (AHRD V1 ***- P93615_WHEAT); contains Interpro domain(s) IPR008390 AWPM-19-like	-1.1	1.13E-06	Awpm-19-like family	
Solyc03g123710.2	Unknown Protein (AHRD V1)	-1.1	4.08E-04	Unknown Protein	
Solyc08g067310.1	CBL-interacting protein kinase 6 (AHRD V1 A0MNL4_POPTR); contains Interpro domain(s) IPR002290 Serine/threonine protein kinase	-1.1	1.03E-03	Cbl-interacting serine threonine-protein kinase 5	GO:0004672; GO:0005524; GO:0006468
Solyc01g014320.2	S-adenosyl-L-methionine salicylic acid carboxyl methyltransferase (AHRD **** A7XZE9_9MAGN); contains Interpro domain(s) IPR005299 SAM dependent carboxyl methyltransferase	-1.09	3.12E-05	Probable s-adenosylmethionine-dependent methyltransferase	GO:0008168
Solyc01g087800.2	Subtilisin-like protease (AHRD V1 **-- O82777_SOLLC); Interpro domain(s) IPR015500 Peptidase S8, subtilisin-related	-1.09	4.57E-04	Subtilisin-like protease	GO:0004252; GO:0042802; GO:0043086
Solyc01g095340.2	Unknown Protein (AHRD V1)	-1.09	8.64E-04	Bag family molecular chaperone regulator 6	
Solyc03g120900.1	Protein transport SEC13-like protein (AHRD V1 Q2PYY1_SOLTU); contains Interpro domain(s) IPR020472 G-protein beta WD-40 repeat, region	-1.09	1.48E-03	Protein transport protein sec13 homolog b	GO:0005515
Solyc05g014280.2	Heat shock protein (AHRD V1 ***- contains Interpro domain(s) IPR002068 Heat shock protein Hsp20	-1.09	5.50E-05	Small heat shock chloroplastic	
Solyc08g078040.2	Monooxygenase FAD-binding (AHRD V1 ***- D8MSJ3_9ENTR); Interpro domain(s) IPR003042 Aromatic-ring hydroxylase-like	-1.09	1.93E-03	Zeaxanthin chloroplastic	GO:0009055; GO:0016491
Solyc08g082190.2	Unknown Protein (AHRD V1)	-1.09	1.22E-04	Keratin-associated protein 6-2-like	
Solyc00g136560.2	Undecaprenyl pyrophosphate synthase (AHRD V1 *-*- contains Interpro domain(s) IPR001441 Di-trans-poly-cis-decaprenylcistransferase-like	-1.08	2.61E-04	Unknown Protein	GO:0016765
Solyc02g063270.2	Multidrug and toxin extrusion protein 1 V1 **-- S47A1_MOUSE); contains Interpro domain(s) IPR015521 MATE family transporter related protein	-1.08	1.82E-06	Mate efflux family protein 5	GO:0006855; GO:0015238; GO:0015297; GO:0016020
Solyc03g119930.1	Sulfate transporter like protein (AHRD V1 Q0WP36_ARATH); contains Interpro domain(s) IPR011547 Sulphate transporter	-1.08	4.92E-07	Molybdate transporter 2	GO:0005215; GO:0006810; GO:0016021
Solyc06g053260.1	Auxin-responsive family protein (AHRD V1 ***- contains Interpro domain(s) IPR003676 Auxin responsive SAUR protein	-1.08	5.10E-06	Auxin-induced protein	
Solyc09g091660.2	ABC transporter G family member 40 V1 ***- AB40G_ARATH); contains Interpro domain(s) IPR013525 ABC-2 type transporter	-1.08	1.80E-07	Pleiotropic drug resistance protein 1	GO:0016020
Solyc02g089510.2	Unknown Protein (AHRD V1)	-1.07	2.86E-03	Zinc finger protein constans	
Solyc07g061890.1	Unknown Protein (AHRD V1)	-1.07	1.55E-03	Unknown protein	
Solyc08g079420.2	Cytochrome P450	-1.07	6.62E-05	Geraniol 8-hydroxylase	GO:0004497; GO:0005506; GO:0020037
Solyc12g013690.1	Monooxygenase FAD-binding protein (AHRD V1 **-- contains Interpro domain(s) IPR003042 Aromatic-ring hydroxylase-like	-1.07	7.62E-05	Zeaxanthin chloroplastic	GO:0009055; GO:0016491
Solyc01g104110.2	Legumin 11S-globulin (AHRD V1 **-- Q39770_GINBI); Interpro domain(s) IPR011051 Cupin, RmlC-type	-1.06	3.66E-04	13s globulin seed storage protein 2	GO:0045735
Solyc01g105660.2	1-aminocyclopropane-1-carboxylate oxidase (AHRD V1 **-- ACCO_TOBAC); Interpro domain(s) IPR005123 Oxoglutarate and iron-dependent oxygenase	-1.06	5.86E-05	Probable 2-oxoglutarate fe -dependent dioxygenase	GO:0016491
Solyc06g076450.2	Ras-related protein Rab-25 (AHRD V1 ***- contains Interpro domain(s) IPR015595 Rab11-related	-1.06	2.23E-03	Ras-related protein rab11a	GO:0005524; GO:0005622; GO:0006355; GO:0008134
Solyc01g103650.2	Hydrolase alpha/beta fold family (AHRD V1 A4VRL9_PSEU5); contains Interpro domain(s) IPR012020 AB-hydrolase YheT, putative	-1.05	1.20E-05	Embryogenesis-associated protein emb8	GO:0004091

Solyc06g007430.1	CBL-interacting protein kinase 11 (AHRD V1 C4P7W0_VITVI); contains Interpro domain(s) IPR002290 Serine/threonine protein kinase	-1.05	3.73E-06	Cbl-interacting protein kinase 2	GO:0004672; GO:0005524; GO:0006468
Solyc08g065320.2	Transmembrane protein 222 (Fragment) (AHRD V1 Q5SSD9_HUMAN); contains Interpro domain(s) IPR008496 Protein of unknown function DUF778	-1.05	6.94E-07	Protein reversion-to-ethylene sensitivity1	
Solyc09g011580.2	Glutathione S-transferase-like protein (AHRD V1 **** contains Interpro domain(s) IPR004046 Glutathione S-transferase, C-terminal	-1.05	2.13E-07	Probable glutathione s-transferase	GO:0004364; GO:0043295
Solyc09g011590.2	Glutathione S-transferase-like protein (AHRD V1 **** contains Interpro domain(s) IPR004046 Glutathione S-transferase, C-terminal	-1.05	4.52E-06	Probable glutathione s-transferase	GO:0004364
Solyc11g008440.1	Amino acid transporter (AHRD V1 **** contains Interpro domain(s) IPR013057 Amino acid transporter, transmembrane	-1.05	2.94E-04	Vacuolar amino acid transporter 1	GO:0015186; GO:0015188; GO:0005302
Solyc04g080700.2	Wound responsive protein (Fragment) (AHRD V1 A6N0L4_ORYSI); contains Interpro domain(s) IPR003729 Protein of unknown function DUF151	-1.04	1.72E-06	Bifunctional nuclease 2-like	
Solyc06g051360.2	2-oxoglutarate-dependent dioxygenase (AHRD V1 ***- B9HHR0_POPTR)	-1.04	1.15E-03	Gibberellin 2-beta-dioxygenase 1	
Solyc08g005090.1	Unknown Protein (AHRD V1)	-1.04	2.72E-03	Suppressor protein srp40	
Solyc12g006530.1	Cycloartenol synthase (AHRD V1 ***- D7LA92_ARALY); Interpro domain(s) IPR018333 Squalene cyclase	-1.04	2.90E-06	Beta-amyrin synthase	GO:0003824
Solyc12g011410.1	Unknown Protein (AHRD V1)	-1.04	1.47E-04	Unknown Protein	
Solyc12g096710.1	Receptor like kinase, RLK	-1.04	4.02E-04	Probably inactive leucine-rich repeat receptor-like protein kinase	GO:0004675; GO:0006468
Solyc03g033410.2	Ubiquitin-conjugating enzyme E2 10 (AHRD V1 UBC10_ARATH); contains Interpro domain(s) IPR000608 Ubiquitin-conjugating enzyme, E2	-1.03	9.21E-05	Ubiquitin-conjugating enzyme e2 10	GO:0019787; GO:0043687; GO:0051246
Solyc06g008300.2	LRR receptor-like serine/threonine-protein kinase, RLP	-1.03	4.47E-04	Low quality protein: probable leucine-rich repeat receptor-like protein kinase	GO:0005515
Solyc07g042630.2	Beta-Amyrin Synthase (AHRD V1 **** O82146_PANGI); Interpro domain(s) IPR018333 Squalene cyclase	-1.03	1.36E-05	Lupeol synthase	GO:0003824
Solyc07g065160.2	Pirin (AHRD V1 ***- D7RJ70_CARPA); contains domain(s) IPR012093 Pirin	-1.03	3.60E-04	Pirin-like protein	GO:0003712; GO:0005516
Solyc11g066250.1	Serine carboxypeptidase (AHRD V1 **** Q9XH61_9ASTR); Interpro domain(s) IPR001563 Peptidase S10, serine carboxypeptidase	-1.03	7.18E-05	Serine carboxypeptidase-	GO:0004185; GO:0006508
Solyc11g066860.1	Os02g0448600 protein (Fragment) (AHRD V1 *---	-1.03	9.15E-06	Unknown Protein	
Solyc00g050130.1	UDP-glucose glucosyltransferase (AHRD V1 **** D2KTV5_LOTJA); Interpro domain(s) IPR002213 UDP-glucuronosyl/UDP-glucosyltransferase	-1.02	1.93E-03	Unknown Protein	GO:0008152; GO:0016758
Solyc01g095720.2	Lipase (AHRD V1 *-*- C1FIH0_9CHLO); contains domain(s) IPR002921 Lipase, class 3	-1.02	1.06E-05	Alpha beta-hydrolases superfamily protein	GO:0004806; GO:0006629
Solyc04g076990.2	Receptor like kinase, RLK	-1.02	3.07E-06	Receptor-like protein kinase haiku2	GO:0005515; GO:0004675
Solyc06g073500.2	Unknown Protein (AHRD V1)	-1.02	4.31E-04	Pentatricopeptide repeat superfamily protein	
Solyc01g080220.2	Dienelactone hydrolase family protein (AHRD V1 D7L3C7_ARALY); contains Interpro domain(s) IPR002925 Dienelactone hydrolase	-1.01	1.03E-06	Endo- -beta-d-glucanase	GO:0016787
Solyc02g079930.2	Phosphosulfolactate synthase (AHRD V1 ***- Q1ASZ8_RUBXD); Interpro domain(s) IPR003830 (2R)-phospho-3-sulpholactate synthase, ComA	-1.01	3.17E-04	Phosphosulfolactate synthase-related protein	GO:0019295
Solyc06g068600.2	Phosphate import ATP-binding protein pstB 1 V1 **** B6UHM7_MAIZE); contains Interpro domain(s) IPR005670 Phosphate transport system permease protein 1	-1.01	1.85E-05	Abc transporter i family member 17	GO:0005524; GO:0016887
Solyc12g088940.1	Protein serine/threonine kinase (AHRD V1 *-*- contains Interpro domain(s) IPR002290 Serine/threonine protein kinase	-1.01	1.36E-03	Mitogen-activated protein kinase kinase kinase yoda	GO:0006468
Solyc01g104690.2	Unknown Protein (AHRD V1)	-1	8.37E-05	Unknown protein	
Solyc04g007000.1	Ethylene-responsive transcription factor 4 (AHRD V1 ERF78_ARATH); contains Interpro domain(s) IPR003340 Transcriptional factor B3	-1	4.48E-04	Ap2 erf and b3 domain-containing transcription factor rav1	GO:0003700; GO:0006355
Solyc04g016230.2	Glucosyltransferase (AHRD V1 **** D7URL8_9LAMI); contains domain(s) IPR002213 UDP-glucuronosyl/UDP-glucosyltransferase	-1	4.15E-05	Zeatin o-glucosyltransferase	GO:0008152; GO:0016758

Solyc06g051940.2	Protein phosphatase 2C (AHRD V1 **** contains Interpro domain(s) IPR015655 Protein phosphatase 2C	-1	2.72E-04	Probable protein phosphatase 2c 51	GO:0003824; GO:0004722; GO:0008022
Solyc06g062460.2	BHLH transcription factor-like (AHRD V1 *--* contains Interpro domain(s) IPR001092 Basic helix-loop-helix dimerisation region bHLH	-1	1.35E-04	Transcription factor bhlh87-	GO:0005634; GO:0030528; GO:0045449
Solyc04g081770.2	GDSL esterase/lipase At5g42170 (AHRD V1 ***- contains Interpro domain(s) IPR001087 Lipase, GDSL	-0.99	1.38E-06	Gdsl esterase lipase exl3	GO:0006629; GO:0016788
Solyc05g013630.1	Unknown protein (Fragment) (AHRD V1 ***-	-0.99	1.70E-04	Cp-interacting protein-l	
Solyc08g069060.2	Beta-1 3-galactosyltransferase 6 (AHRD V1 **** contains Interpro domain(s) IPR002659 Glycosyl transferase, family 31	-0.99	4.49E-04	Beta- -galactosyltransferase 7	GO:0006486; GO:0008378; GO:0016020
Solyc12g019740.1	Thioredoxin family protein (AHRD V1 ***- contains Interpro domain(s) IPR015467 Thioredoxin, core	-0.99	9.18E-04	Thioredoxin-like 1- chloroplastic	GO:0045454; GO:0016671
Solyc12g044950.1	Omega-6 fatty acid desaturase (AHRD V1 Q461Q1_HEVBR); contains Interpro domain(s) IPR005804 Fatty acid desaturase, type 1	-0.99	1.82E-03	Omega-6 fatty acid endoplasmic reticulum isozyme 2	GO:0006629; GO:0016491
Solyc12g098190.1	F-box protein PP2-B1 (AHRD V1 ***- contains Interpro domain(s) IPR001810 Cyclin-like F-box	-0.99	1.24E-03	F-box protein pp2-b10	
Solyc06g051020.2	Peptide-N4-(N-acetyl-beta-glucosaminyl)asparagine amidase A (AHRD V1 *-*-	-0.98	2.95E-05	Peptide-n4-(n-acetyl-beta-glucosaminyl)asparagine amidase a	
Solyc06g053670.1	Enoyl-CoA hydratase/isomerase family protein (AHRD V1 D7MH17_ARALY); contains Interpro domain(s) IPR001753 Crotonase, core	-0.98	3.79E-04	Enoyl- hydratase domain-containing protein mitochondrial	GO:0003824; GO:0008152
Solyc06g071820.2	Speckle-type poz protein (AHRD V1 *--- contains Interpro domain(s) IPR000197 Zinc finger, TAZ-type	-0.98	2.27E-04	Btb poz and taz domain-containing protein 1	GO:0005515
Solyc12g094620.1	Catalase (AHRD V1 ***- Q2PYW5_SOLTU); contains domain(s) IPR018028 Catalase related subgroup	-0.98	2.35E-03	Catalase isozyme 1	GO:0004096; GO:0005506; GO:0006979; GO:0020037
Solyc02g079960.2	Thioredoxin h (AHRD V1 ***- Q9XF70_HEVBR); Interpro domain(s) IPR015467 Thioredoxin, core	-0.97	3.65E-04	Thioredoxin-like protein cxxs1	GO:0045454
Solyc04g007540.1	Multidrug resistance protein mdtK (AHRD V1 MDTK_ENT38); contains Interpro domain(s) IPR015521 MATE family transporter related protein	-0.97	3.01E-04	Mate efflux family protein 5-	GO:0006855; GO:0015238; GO:0015297; GO:0016020
Solyc04g053130.2	LHC-related protein (AHRD V1 **-- D8SUY7_SELML)	-0.97	1.30E-05	Stress enhanced protein chloroplastic	GO:0016168
Solyc04g077230.1	Unknown Protein (AHRD V1)	-0.97	2.55E-04	Unknown protein	
Solyc09g007980.1	Unknown Protein (AHRD V1)	-0.97	2.87E-03	Unknown protein	
Solyc01g008420.2	Mate efflux family protein (AHRD V1 D7KHQ9_ARALY); contains Interpro domain(s) IPR002528 Multi antimicrobial extrusion protein MatE	-0.96	2.94E-04	Mate efflux family protein 1	GO:0006855; GO:0015238; GO:0015297; GO:0016020
Solyc03g098000.2	At1g32160/F3C3_6 (AHRD V1 ***- Q9FVR1_ARATH); contains domain(s) IPR008479 Protein of unknown function DUF760	-0.96	1.12E-03	Alanine--trna ligase	
Solyc03g111310.2	AKIN gamma (AHRD V1 **-- Q7XYY0_MEDTR); Interpro domain(s) IPR000644 Cystathionine beta-synthase, core	-0.96	1.04E-05	Snf1-related protein kinase regulatory subunit gamma-1	
Solyc03g116570.2	YuxK (AHRD V1 **-- B4AHQ1_BACPU); contains domain(s) IPR007263 Putative thiol-disulphide oxidoreductase DCC	-0.96	1.43E-04	Dcc family protein chloroplastic	
Solyc04g071900.2	Peroxidase (AHRD V1 **** Q94IQ1_TOBAC); contains domain(s) IPR002016 Haem peroxidase, plant/fungal/bacterial	-0.96	1.03E-04	Peroxidase 12	GO:0004601; GO:0006979; GO:0020037; GO:0055114
Solyc08g028690.2	Tasselseed2-like short-chain dehydrogenase/reductase (Fragment) (AHRD V1 Q2EFE2_9POAL); contains Interpro domain(s) IPR002347 Glucose/ribitol dehydrogenase	-0.96	2.85E-04	Secoisolariciresinol dehydrogenase	GO:0008152; GO:0016491
Solyc01g086870.2	BHLH transcription factor (AHRD V1 *-** contains Interpro domain(s) IPR011598 Helix-loop-helix DNA-binding	-0.95	1.51E-04	Transcription factor bhlh130	GO:0005634; GO:0030528; GO:0045449
Solyc01g106400.2	Peptide methionine sulfoxide reductase msrB (AHRD ***- B6T2C6_MAIZE); contains Interpro domain(s) IPR002579 Methionine sulphoxide reductase B	-0.95	1.73E-05	Peptide methionine sulfoxide reductase b5	GO:0055114
Solyc02g063440.2	Unknown Protein (AHRD V1)	-0.95	1.64E-03	Unknown protein	GO:0005488
Solyc02g079430.2	CONSTANS-like zinc finger protein (AHRD V1 D0EP04_SOYBN); contains Interpro domain(s) IPR000315 Zinc finger, B-box	-0.95	2.46E-04	Zinc finger protein constans	GO:0005622; GO:0008270
Solyc02g085940.2	Unknown Protein (AHRD V1)	-0.95	9.63E-06	Ribulose bisphosphate carboxylase	

Solyc02g093750.2	Ring zinc finger protein (Fragment) (AHRD *--- A6MH00_LILLO); contains Interpro domain(s) IPR008166 Protein of unknown function DUF23	-0.95	9.67E-04	Upf0392 protein rcom	
Solyc04g074850.2	Multidrug resistance protein mdtK (AHRD V1 MDTK_YERP3); contains Interpro domain(s) IPR002528 Multi antimicrobial extrusion protein MatE	-0.95	7.72E-05	Protein transparent testa 12	GO:0006855; GO:0015238; GO:0015297; GO:0016020
Solyc04g080640.1	Genomic DNA chromosome 5 TAC clone (AHRD V1 ***- Q9FJZ5_ARATH)	-0.95	1.69E-03	Unknown protein	
Solyc09g089730.2	1-aminocyclopropane-1-carboxylate oxidase-like protein (AHRD V1 **-- contains Interpro domain(s) IPR005123 Oxoglutarate and iron-dependent oxygenase	-0.95	1.55E-03	1-aminocyclopropane-1-carboxylate oxidase homolog	GO:0016491
Solyc11g010700.1	Receptor-like protein kinase (AHRD V1 *-*- contains Interpro domain(s) IPR001245 Tyrosine protein kinase	-0.95	7.69E-05	U-box domain-containing protein 50	GO:0006468
Solyc01g102960.2	class IV heat shock protein (AHRD ***- B6T3F5_MAIZE); contains Interpro domain(s) IPR002068 Heat shock protein Hsp20	-0.94	2.14E-03	Kda class iv heat shock	
Solyc01g106000.2	Isochorismatase hydrolase (AHRD V1 ***- B6TNR9_MAIZE); Interpro domain(s) IPR000868 Isochorismatase-like	-0.94	1.70E-05	Nicotinamidase 1	GO:0003824; GO:0008152
Solyc02g071020.2	Unknown Protein (AHRD V1)	-0.94	3.23E-05	Chlorophyll a-b binding protein chloroplastic	
Solyc02g086880.2	Formate dehydrogenase (AHRD V1 **** Q5NE18_SOLLC); Interpro domain(s) IPR006140 D-isomer specific 2-hydroxyacid dehydrogenase, NAD-binding	-0.94	2.21E-05	Formate mitochondrial	GO:0003824; GO:0005488; GO:0008152
Solyc03g112060.2	Quinolinate synthase A (AHRD V1 *-*- contains Interpro domain(s) IPR003808 Fe-S metabolism associated SufE	-0.94	9.18E-04	Quinolinate chloroplastic	GO:0008987; GO:0009435
Solyc04g057940.2	U-box domain-containing protein (AHRD V1 *-*- contains Interpro domain(s) IPR017986 WD40 repeat, region	-0.94	7.66E-05	E3 ubiquitin-protein ligase lin-1	GO:0005488
Solyc04g072240.2	Oxidoreductase family protein (AHRD V1 ***- contains Interpro domain(s) IPR016040 NAD(P)-binding domain	-0.94	1.06E-04	Uncharacterized oxidoreductase	GO:0003824; GO:0005488; GO:0008152
Solyc05g006420.2	Two-component response regulator ARR3 (AHRD V1 B6TSM4_MAIZE); contains Interpro domain(s) IPR001789 Signal transduction response regulator, receiver region	-0.94	1.74E-06	Two-component response regulator arr5	GO:0000156; GO:0000160; GO:0006355
Solyc10g012080.2	MRNA clone RAFL21-79-C21 (AHRD V1 **-- contains Interpro domain(s) IPR006461 Protein of unknown function Cys-rich	-0.94	8.35E-04	Pollen-specific leucine-rich repeat extensin-like protein 3	
Solyc10g076550.1	Receptor-like protein kinase At3g21340 (AHRD V1 RLK6_ARATH); contains Interpro domain(s) IPR002290 Serine/threonine protein kinase	-0.94	2.55E-04	Wall-associated receptor kinase 2	GO:0004672; GO:0005524; GO:0006468
Solyc11g066740.1	Os12g0283800 protein (Fragment) (AHRD V1 *-*-	-0.94	7.41E-04	Protein reticulata-related chloroplastic	
Solyc01g094790.2	Cysteine synthase (AHRD V1 ***- Q9FS29_SOLTU); Interpro domain(s) IPR005859 Cysteine synthase A	-0.93	5.66E-04	Bifunctional l-3-cyanoalanine synthase cysteine synthase mitochondrial	GO:0004124; GO:0006535
Solyc02g063450.2	Hypothetical YFW family protein 5 (AHRD *--- Q5AP84_CANAL); contains Interpro domain(s) IPR009836 Protein of unknown function DUF1399	-0.93	4.63E-05	Unknown protein	
Solyc02g083280.2	Thiosulfate sulfurtransferase/rhodanese-like domain-containing protein 1 (AHRD *--* TSTD1_HUMAN); contains Interpro domain(s) IPR001763 Rhodanese-like	-0.93	7.48E-04	Thiosulfate sulfurtransferase chloroplastic-	GO:0004792
Solyc04g079220.1	Patatin-like protein 1 (AHRD V1 ***- contains Interpro domain(s) IPR002641 Patatin	-0.93	4.23E-04	Patatin-like protein 2	GO:0006629
Solyc07g006480.2	LRR receptor-like serine/threonine-protein kinase FEI 1 V1 **** FEI1_ARATH); contains Interpro domain(s) IPR002290 Serine/threonine protein kinase	-0.93	3.01E-04	Probably inactive leucine-rich repeat receptor-like protein kinase	GO:0004675; GO:0006468
Solyc07g055470.2	Cytochrome P450	-0.93	4.11E-05	Cytochrome p450 cyp72a219	
Solyc02g078210.2	Ubiquitin-conjugating enzyme 22 (AHRD V1 ***- contains Interpro domain(s) IPR000608 Ubiquitin-conjugating enzyme, E2	-0.92	6.78E-05	Probable ubiquitin-conjugating enzyme e2 24	GO:0019787; GO:0043687; GO:0051246
Solyc03g116700.2	Blue copper protein (AHRD V1 **-- contains Interpro domain(s) IPR003245 Plastocyanin-like	-0.92	3.48E-04	Cucumber peeling cupredoxin	GO:0005507; GO:0009055
Solyc04g079230.2	Patatin-like protein 1 (AHRD V1 ***- contains Interpro domain(s) IPR002641 Patatin	-0.92	6.57E-06	Patatin-like protein 2	GO:0006629
Solyc05g013810.2	Glycosyl hydrolase family 5 protein/cellulase (AHRD **-- A8NVS8_COPC7); contains Interpro domain(s) IPR001547 Glycoside hydrolase, family 5	-0.92	8.25E-05	Cellulase protein	GO:0003824; GO:0005975; GO:0043169
Solyc06g019170.2	Gamma-glutamyl phosphate reductase (AHRD V1 ***- contains Interpro domain(s) IPR005766 Delta l-pyrroline-5-carboxylate synthetase	-0.92	7.95E-04	Delta-1-pyrroline-5-carboxylate synthase	GO:0004349; GO:0005737; GO:0006561

Solyc09g074550.2	UPF0497 membrane protein 6 (AHRD V1 U4976_VITVI); contains Interpro domain(s) IPR006459 Uncharacterised protein family UPF0497, trans-membrane plant subgroup	-0.92	1.50E-03	Casp-like protein 1e2	GO:0005515
Solyc11g028080.1	Unknown Protein (AHRD V1)	-0.92	4.11E-04	Unknown protein	
Solyc12g099190.1	Invertase inhibitor (AHRD V1 ***- C1KBP2_SOLTU); Interpro domain(s) IPR006501 Pectinesterase inhibitor	-0.92	1.12E-03	Invertase inhibitor	GO:0004857; GO:0030599
Solyc01g080870.2	Peptide transporter-like protein (AHRD V1 **-* contains Interpro domain(s) IPR000109 TGF-beta receptor, type I/II extracellular region	-0.91	1.80E-04	Protein nrt1 ptr family	GO:0016020; GO:0042936
Solyc04g076980.2	LRR receptor-like serine/threonine-protein kinase, RLP	-0.91	1.49E-04	Receptor-like protein kinase haiku2	GO:0005515; GO:0004675
Solyc01g099750.2	Heparan-alpha-glucosaminide N-acetyltransferase (AHRD V1 **-- D2QUR2_SPILD); Interpro domain(s) IPR019259 Protein of unknown function DUF2261, transmembrane	-0.9	9.22E-04	Heparan-alpha-glucosaminide n-acetyltransferase	
Solyc03g006410.2	Plant-specific domain TIGR01615 family protein (AHRD ***- B6UDN7_MAIZE); contains Interpro domain(s) IPR006502 Protein of unknown function DUF506, plant	-0.9	7.75E-04	Unknown protein	
Solyc03g114150.2	Aldehyde dehydrogenase (AHRD V1 **** Q1AFF6_9ROSI); Interpro domain(s) IPR015590 Aldehyde dehydrogenase	-0.9	1.52E-04	Aldehyde dehydrogenase family 2 member mitochondrial	GO:0008152; GO:0016491
Solyc04g009960.2	L-allo-threonine aldolase (AHRD V1 **** D7RJ69_CARPA); Interpro domain(s) IPR001597 Aromatic amino acid beta-eliminating lyase/threonine aldolase	-0.9	1.01E-04	Probable low-specificity l-threonine aldolase 1	GO:0003824; GO:0030170
Solyc09g098030.2	Cytochrome P450	-0.9	2.48E-06	Geraniol 8-hydroxylase	GO:0004497; GO:0005506; GO:0020037
Solyc12g088250.1	Serine carboxypeptidase 1 (AHRD V1 ***- contains Interpro domain(s) IPR001563 Peptidase S10, serine carboxypeptidase	-0.9	4.45E-05	Serine carboxypeptidase	GO:0004185; GO:0006508
Solyc02g077590.1	Homeobox-leucine zipper-like protein (AHRD V1 *-*- contains Interpro domain(s) IPR001356 Homeobox	-0.89	1.00E-05	Homeobox-leucine zipper protein athb-52	GO:0003677; GO:0045449
Solyc03g097930.2	Unknown Protein (AHRD V1)	-0.89	1.26E-03	Potassium channel skor	
Solyc04g008330.1	Glucosyltransferase (AHRD V1 **** Q2VA65_SOYBN); contains domain(s) IPR002213 UDP-glucuronosyl/UDP-glucosyltransferase	-0.89	1.81E-05	Zeatin o-glucosyltransferase	GO:0008152; GO:0016758
Solyc09g011660.2	Universal stress protein 1 (AHRD V1 B0YQX0_GOSAR); contains Interpro domain(s) IPR006016 UspA	-0.89	7.61E-04	Universal stress protein a-like protein	GO:0006950
Solyc10g080610.1	Kelch-like protein 14 (AHRD V1 ***- contains Interpro domain(s) IPR015915 Kelch-type beta propeller	-0.89	1.74E-03	F-box kelch-repeat protein	
Solyc12g042380.1	MtN19-like protein (AHRD V1 ***- Q64EX4_PEA); Interpro domain(s) IPR011692 Stress up-regulated Nod 19	-0.89	6.24E-04	19-like isoform 2	
Solyc02g068080.2	Voltage-gated chloride channel (AHRD V1 **** contains Interpro domain(s) IPR002251 Chloride channel plant CLC	-0.88	5.57E-04	Chloride channel protein clc-b	GO:0016020; GO:0005247
Solyc02g082060.1	PPPDE peptidase domain-containing protein 1 (AHRD *--- PPDE1_XENLA); contains Interpro domain(s) IPR008580 Protein of unknown function DUF862, eukaryotic	-0.88	5.26E-05	Desi-like protein	
Solyc04g011670.2	BZIP transcription factor (AHRD V1 **** contains Interpro domain(s) IPR011616 bZIP transcription factor, bZIP-1	-0.88	2.18E-04	Tgacg-sequence-specific dna-binding protein tga-1a	GO:0003700; GO:0005634; GO:0006355; GO:0043565; GO:0046983
Solyc04g025040.1	Unknown Protein (AHRD V1)	-0.88	7.27E-04	Rna-binding protein fus	
Solyc05g015800.2	Acetyl coenzyme A cis-3-hexen-1-ol acetyl transferase V1 **-* Q9SRQ2_ARATH); contains Interpro domain(s) IPR003480 Transferase	-0.88	2.09E-05	Methanol o-anthraniloyltransferase	GO:0016747
Solyc08g076450.2	3-beta hydroxysteroid dehydrogenase/isomerase family protein (AHRD **-- B6TV09_MAIZE); contains Interpro domain(s) IPR016040 NAD(P)-binding domain	-0.88	9.89E-04	Nad -binding rossmann-fold superfamily protein	GO:0003824; GO:0005488; GO:0008152
Solyc09g007270.2	Ascorbate peroxidase (AHRD V1 **** B9VRH6_CITMA); Interpro domain(s) IPR002207 Plant ascorbate peroxidase	-0.88	2.71E-05	L-ascorbate peroxidase cytosolic	GO:0004601; GO:0006979; GO:0020037; GO:0055114
Solyc10g007070.2	CT099 (Fragment) (AHRD V1 *--- Q30GY4_9SOLN); Interpro domain(s) IPR003245 Plastocyanin-like	-0.88	2.41E-03	Early nodulin-like protein 2	GO:0009055
Solyc11g007200.1	Copper chaperone (AHRD V1 *-*- D3GC04_9ROSI); Interpro domain(s) IPR006121 Heavy metal transport/detoxification protein	-0.88	7.15E-06	Copper transport protein cch-	GO:0030001; GO:0046872
Solyc01g108300.2	Myb family transcription factor (AHRD V1 D7LQI3_ARALY); contains Interpro domain(s) IPR006447 Myb-like DNA-binding region, SHAQKYF class	-0.87	2.31E-04	Two-component response regulator-like aprr2	GO:0003677; GO:0045449

Solyc07g054760.1	Wound induced protein (AHRD V1 ***-	-0.87	2.83E-03	Wound induced protein	
Solyc11g013150.1	GRAS family transcription factor (AHRD V1 B9H7C5_POPTR); contains Interpro domain(s) IPR005202 GRAS transcription factor	-0.87	3.61E-04	Nodulation-signaling pathway 2 protein	
Solyc04g079210.2	Patatin-like protein 1 (AHRD V1 ***- contains Interpro domain(s) IPR016035 Acyl transferase/acyl hydrolase/lysophospholipase	-0.86	1.53E-05	Patatin-like protein 2	GO:0008152
Solyc04g079410.2	Single-stranded DNA binding protein (AHRD V1 D7KGI0_ARALY); contains Interpro domain(s) IPR000424 Primosome PriB/single-strand DNA-binding	-0.86	2.80E-03	Protein mitochondrial	GO:0003697
Solyc05g005920.2	Peptide transporter (AHRD V1 **-* A6YJX4_9MAGN); Interpro domain(s) IPR000109 TGF-beta receptor, type I/II extracellular region	-0.86	5.80E-04	Protein nrt1 ptr family	GO:0080054; GO:0016020; GO:0042936
Solyc05g010040.2	Unknown Protein (AHRD V1)	-0.86	1.02E-03	Unknown protein	
Solyc07g044980.2	NPR1-1 protein (Fragment) (AHRD V1 ***- contains Interpro domain(s) IPR013069 BTB/POZ	-0.86	2.20E-05	Regulatory protein npr3-like isoform x1	GO:0005515
Solyc07g063750.2	Serine/threonine-protein kinase receptor (AHRD V1 **** contains Interpro domain(s) IPR002290 Serine/threonine protein kinase	-0.85	9.38E-05	G-type lectin s-receptor-like serine threonine-protein kinase at4g27290	GO:0048544
Solyc09g011560.2	Glutathione S-transferase-like protein (AHRD V1 **** contains Interpro domain(s) IPR004046 Glutathione S-transferase, C-terminal	-0.85	4.38E-04	Probable glutathione s-transferase	GO:0004364
Solyc09g090110.2	Actin depolymerizing factor 6 (AHRD V1 Q8H9D5_SOLTU); contains Interpro domain(s) IPR002108 Actin-binding, cofilin/tropomyosin type	-0.85	1.38E-05	Actin-depolymerizing factor	GO:0003779; GO:0005622
Solyc12g006450.1	Aminotransferase-like protein (AHRD V1 ***- Q9LIE2_ARATH); Interpro domain(s) IPR005814 Aminotransferase class-III	-0.85	1.28E-05	Gamma aminobutyrate transaminase chloroplastic isoform x2	GO:0003824; GO:0030170
Solyc04g078200.2	Gibberellin-regulated family protein (AHRD V1 **-- contains Interpro domain(s) IPR003854 Gibberellin regulated protein	-0.84	1.47E-04	Snakin-1	
Solyc09g097780.2	Glycine-rich protein (AHRD V1 *-*- D2K2U2_TOBAC); Interpro domain(s) IPR010800 Glycine rich	-0.84	6.78E-05	Glycine-rich protein precursor	
Solyc10g049970.1	Kynurenine formamidase (AHRD V1 **-- D7BCL0_MEISD); Interpro domain(s) IPR007325 Putative cyclase	-0.84	1.64E-05	Kynurenine formamidase	GO:0009975; GO:0004061
Solyc02g030300.2	Serine/threonine-protein kinase receptor (AHRD V1 **** contains Interpro domain(s) IPR002290 Serine/threonine protein kinase	-0.83	1.81E-04	G-type lectin s-receptor-like serine threonine-protein kinase at4g27290	GO:0048544
Solyc03g025720.2	Long-chain-fatty-acid--CoA ligase (AHRD V1 **** C3JN12_RHOER); Interpro domain(s) IPR000873 AMP-dependent synthetase and ligase	-0.83	5.81E-04	4-coumarate-- ligase	GO:0003824; GO:0008152
Solyc07g052950.2	Unknown Protein (AHRD V1)	-0.83	4.98E-04	Unknown protein	
Solyc07g062970.2	Serine/threonine phosphatase family protein (AHRD V1 C1E0J1_9CHLO); contains Interpro domain(s) IPR015655 Protein phosphatase 2C	-0.83	7.49E-04	Probable protein phosphatase 2c 39	GO:0003824; GO:0004722
Solyc07g063910.2	Unknown Protein (AHRD V1)	-0.83	7.78E-04	Unknown protein	
Solyc09g072590.2	Actin-depolymerizing factor 6 (AHRD V1 **** contains Interpro domain(s) IPR002108 Actin-binding, cofilin/tropomyosin type	-0.83	8.96E-05	Actin-depolymerizing factor	GO:0003779; GO:0005622
Solyc09g075020.2	Multidrug resistance protein ABC transporter family V1 **-- B9GS96_POPTR); contains Interpro domain(s) IPR001140 ABC transporter, transmembrane region	-0.83	2.78E-03	Abc transporter c family member 14-	GO:0005524; GO:0016887
Solyc09g089860.2	Nodulin-like protein (AHRD V1 ***- B6TRP3_MAIZE); Interpro domain(s) IPR000620 Protein of unknown function DUF6, transmembrane	-0.83	2.00E-03	Wat1-related protein at5g07050-	GO:0016020
Solyc11g069450.1	Arabidopsis thaliana genomic DNA chromosome 5 clone MOK16 (AHRD V1 **-- Q9LYW2_ARATH); contains Interpro domain(s) IPR007608 Protein of unknown function	-0.83	1.04E-03	Unknown protein	GO:0005515
Solyc12g042470.1	Reductase 2 (AHRD V1 **** Q6TY50_HYDMC); Interpro domain(s) IPR020471 Aldo/keto reductase subgroup	-0.83	3.91E-04	Methylecgonone reductase-	GO:0016491; GO:0055114
Solyc12g089220.1	Wound responsive protein (Fragment) (AHRD V1 A6N0L4_ORYSI); contains Interpro domain(s) IPR003729 Protein of unknown function DUF151	-0.83	4.18E-04	Bifunctional nuclease 1-	
Solyc12g099600.1	Protein phosphatase 2C containing protein (AHRD ***- B6T998_MAIZE); contains Interpro domain(s) IPR015655 Protein phosphatase 2C	-0.83	7.85E-05	Probable protein phosphatase 2c 40	GO:0003824
Solyc02g065000.1	Calmodulin-like protein (AHRD V1 ***- Q67TZ4_ORYSJ); Interpro domain(s) IPR011992 EF-Hand type	-0.82	9.35E-04	Calmodulin-like protein 1	GO:0005509

Solyc02g081550.2	ATP-dependent Zn protease cell division protein homolog (AHRD V1 **** B2XTF7_HETA2); contains Interpro domain(s) IPR001315 Caspase Recruitment IPR003960 ATPase, AAA-type,	-0.82	1.89E-03	Atp-dependent zinc metalloprotease ftsh chloroplastic-	GO:0005515; GO:0005622; GO:0042981
Solyc04g005160.1	6-phosphogluconate dehydrogenase decarboxylating (AHRD V1 **** contains Interpro domain(s) IPR006113 6-phosphogluconate dehydrogenase, decarboxylating	-0.82	3.07E-05	6-phosphogluconate decarboxylating 3	GO:0004616; GO:0016491; GO:0042802
Solyc04g016430.2	Cytokinin oxidase/dehydrogenase 1 (AHRD V1 **** contains Interpro domain(s) IPR015345 Cytokinin dehydrogenase 1, FAD and cytokinin binding	-0.82	3.52E-04	Cytokinin dehydrogenase 1	GO:0016491; GO:0050660
Solyc05g006740.2	Glutathione S-transferase (AHRD V1 **** A3FF38_9CARY); Interpro domain(s) IPR004046 Glutathione S-transferase, C-terminal	-0.82	5.61E-04	Glutathione s-transferase u17	GO:0004364
Solyc05g010450.1	Unknown Protein (AHRD V1)	-0.82	2.95E-04	Micronuclear linker histone poly	
Solyc07g062060.2	Peptide methionine sulfoxide reductase msrB (AHRD ***- B6T2N3_MAIZE); contains Interpro domain(s) IPR002579 Methionine sulphoxide reductase B	-0.82	4.34E-04	Peptide methionine sulfoxide reductase chloroplastic	GO:0055114
Solyc11g064920.1	Dihydropyrimidinase (AHRD V1 **-* D7M4H1_ARALY); contains domain(s) IPR011778 D-hydantoinase	-0.82	5.97E-05	Dihydropyrimidinase	GO:0016810
Solyc12g096030.1	Mitochondrial carrier-like protein (AHRD V1 ***- contains Interpro domain(s) IPR002067 Mitochondrial carrier protein	-0.82	1.64E-03	Solute carrier family 25 member 44	GO:0016020
Solyc01g090680.2	Genomic DNA chromosome 5 TAC clone (AHRD V1 *-*- Q9FKH0_ARATH)	-0.81	2.31E-03	Unknown protein	
Solyc01g087620.2	Unknown Protein (AHRD V1)	-0.8	7.35E-05	Ubiquitin-like protein 5	
Solyc04g014530.1	Ethylene responsive transcription factor 1a (AHRD *-*- C0J9I9_9ROSA); contains Interpro domain(s) IPR001471 Pathogenesis-related transcriptional factor and ERF, DNA-binding	-0.8	1.66E-03	Ethylene-responsive transcription factor 1b	GO:0003700; GO:0006355
Solyc04g017720.2	Gibberellin regulated protein (AHRD V1 **-- contains Interpro domain(s) IPR003854 Gibberellin regulated protein	-0.8	2.90E-03	Protein gast1	
Solyc06g074940.2	ATP-binding cassette protein (AHRD V1 ***- contains Interpro domain(s) IPR003439 ABC transporter-like	-0.8	8.53E-04	Abc transporter f family member 1	GO:0005524; GO:0016887
Solyc08g067540.1	Non-specific lipid-transfer protein (AHRD V1 ***- contains Interpro domain(s) IPR013770 Plant lipid transfer protein and hydrophobic protein, helical	-0.8	1.80E-04	Non-specific lipid-transfer protein 1	GO:0006869
Solyc08g074490.2	Regulatory protein (AHRD V1 **-- Q8LFS2_ARATH)	-0.8	1.27E-03	Calcium-dependent protein kinase	
Solyc10g083380.1	Unknown Protein (AHRD V1); contains Interpro IPR004827 Basic-leucine zipper (bZIP) transcription factor	-0.8	2.42E-03	Bzip transcription factor family protein	GO:0003700; GO:0006355; GO:0043565
Solyc00g289230.1	Receptor protein kinase (AHRD V1 **** contains Interpro domain(s) IPR002290 Serine/threonine protein kinase	-0.79	2.51E-03	Unknown Protein	GO:0004674; GO:0006468
Solyc01g099840.2	Auxin-repressed protein (AHRD V1 ***- B4FA62_MAIZE); Interpro domain(s) IPR008406 Dormancyauxin associated	-0.79	8.37E-04	Auxin-repressed kda	
Solyc03g082690.2	U-box domain-containing protein (AHRD V1 *-*- contains Interpro domain(s) IPR011989 Armadillo-like helical	-0.79	7.55E-04	U-box domain-containing protein 44-	GO:0005488
Solyc03g112640.2	CRAL/TRIO domain containing protein (AHRD V1 B6UD95_MAIZE); contains Interpro domain(s) IPR001251 Cellular retinaldehyde-binding/triple function, C-terminal	-0.79	1.57E-03	Sec14p-like phosphatidylinositol transfer family protein	GO:0008526
Solyc05g052030.1	Ethylene responsive transcription factor 1a (AHRD *-*- C0J9I9_9ROSA); contains Interpro domain(s) IPR001471 Pathogenesis-related transcriptional factor and ERF, DNA-binding	-0.79	2.25E-03	Ethylene-responsive transcription factor erf106	GO:0003700; GO:0006355
Solyc08g068150.2	BURP domain-containing protein (AHRD V1 **-- contains Interpro domain(s) IPR004873 BURP	-0.79	5.14E-04	Dehydration-responsive protein rd22	
Solyc09g061840.2	3-ketoacyl CoA thiolase 1 (AHRD V1 C8YNG6_PETHY); contains Interpro domain(s) IPR002155 Thiolase	-0.79	9.93E-04	3-ketoacyl- thiolase peroxisomal	GO:0003824; GO:0008152
Solyc09g090430.2	Cyanate hydratase (AHRD V1 ***- B6TTW1_MAIZE); Interpro domain(s) IPR008076 Cyanase	-0.79	2.16E-04	Cyanate hydratase	GO:0008824; GO:0009439
Solyc10g083860.1	UDP-glucosyltransferase family 1 protein (AHRD V1 C6KI43_CITSI); contains Interpro domain(s) IPR002213 UDP-glucuronosyl/UDP-glucosyltransferase	-0.79	1.06E-04	Udp-glycosyltransferase 73c6	GO:0008152; GO:0016758
Solyc01g007920.2	Isochorismatase family protein (AHRD V1 ***- contains Interpro domain(s) IPR000868 Isochorismatase-like	-0.78	5.45E-04	Isochorismatase hydrolase family protein	GO:0003824; GO:0008152
Solyc01g009860.2	NAC domain transcription factor (AHRD V1 Q5DM36_WHEAT); contains Interpro domain(s) IPR003441 No apical meristem (NAM) protein	-0.78	9.71E-05	Nac transcription factor 29	GO:0003677; GO:0045449

Solyc09g010140.1	Arabidopsis thaliana genomic DNA chromosome 5 clone MOK16 (AHRD V1 *--- Q9LYW2_ARATH); contains Interpro domain(s) IPR007608 Protein of unknown function	-0.78	2.23E-03	Probable protein kinase ddb	
Solyc11g011920.1	Glutamate decarboxylase (AHRD V1 ***- Q8LKR4_TOBAC); Interpro domain(s) IPR010107 Glutamate decarboxylase	-0.78	1.29E-03	Glutamate decarboxylase	GO:0003824; GO:0030170
Solyc12g045030.1	Short-chain dehydrogenase/reductase family protein (AHRD V1 D7LV30_ARALY); contains Interpro domain(s) IPR002347 Glucose/ribitol dehydrogenase	-0.78	1.26E-03	Probable l-xylulose reductase	GO:0008152; GO:0016491
Solyc02g082080.1	High affinity copper uptake protein (AHRD **** Q8QH07_DANRE); contains Interpro domain(s) IPR007274 Ctr copper transporter	-0.77	8.61E-05	Copper transporter 5	GO:0005375; GO:0006825; GO:0016021
Solyc05g008290.2	Bilirubin oxidase (AHRD V1 ***- C6PN69_9CLOT); Interpro domain(s) IPR008972 Cupredoxin	-0.77	1.34E-03	Multicopper oxidase lpr1	GO:0055114
Solyc09g090070.1	Inorganic phosphate transporter (AHRD V1 **** contains Interpro domain(s) IPR004738 Phosphate permease	-0.77	6.79E-05	Phosphate transporter	GO:0005315; GO:0016021
Solyc12g015630.1	Genomic DNA chromosome 5 P1 clone (AHRD V1 ***- Q9FM81_ARATH)	-0.77	1.59E-03	Unknown protein	
Solyc01g094910.2	Ferric reductase oxidase (AHRD V1 **-* contains Interpro domain(s) IPR013121 Ferric reductase, NAD binding	-0.76	6.57E-05	Ferric reduction oxidase 2	GO:0005506; GO:0009055; GO:0016021; GO:0016491; GO:0050660
Solyc02g081850.2	Amino acid transporter (AHRD V1 **** contains Interpro domain(s) IPR015606 Cationic amino acid transporter	-0.76	6.84E-05	Cationic amino acid transporter 5	GO:0006810; GO:0016020
Solyc05g055310.2	Copper chaperone (AHRD V1 **** Q84KJ2_SOLLC); Interpro domain(s) IPR006121 Heavy metal transport/detoxification protein	-0.76	9.21E-05	Copper chaperone	GO:0030001; GO:0046872
Solyc06g060250.2	Aldehyde dehydrogenase family protein expressed (AHRD **** Q53NG8_ORYSJ); contains Interpro domain(s) IPR012394 Aldehyde dehydrogenase NAD(P)-dependent IPR015590 Aldehyde dehydrogenase	-0.76	1.63E-04	Aldehyde dehydrogenase family 3 member h1	GO:0008152; GO:0016491
Solyc08g062210.2	Nuclear transcription factor Y subunit A-3 V1 **** B6TQI5_MAIZE); contains Interpro domain(s) IPR001289 CCAAT-binding transcription factor, subunit B	-0.76	2.50E-04	Nuclear transcription factor y subunit a-3	GO:0003677; GO:0016602
Solyc10g078590.1	Unknown Protein (AHRD V1)	-0.76	7.17E-04	Unknown protein	
Solyc03g117810.2	Phosphate import ATP-binding protein pstB 1 V1 **** B6UHM7_MAIZE); contains Interpro domain(s) IPR005670 Phosphate transport system permease protein 1	-0.75	3.87E-04	Abc transporter i family member 17	GO:0005524; GO:0016887
Solyc04g040210.2	Unknown Protein (AHRD V1)	-0.75	2.07E-03	Cysteine-rich and transmembrane domain-containing protein a	
Solyc08g067960.2	CHY zinc finger family protein expressed V1 ***- Q337P2_ORYSJ); contains Interpro domain(s) IPR008913 Zinc finger, CHY-type	-0.75	9.96E-04	Ring finger and chy zinc finger domain-containing protein 1	GO:0008270; GO:0005102
Solyc08g082640.2	Cellulose synthase (AHRD V1 **** B9IMB3_POPTR); Interpro domain(s) IPR005150 Cellulose synthase	-0.75	8.05E-04	Cellulose synthase-like protein g3	GO:0016020; GO:0016760; GO:0030244
Solyc11g011880.1	RLK, Receptor like protein, putative resistance with an antifungal domain	-0.75	1.09E-03	Cysteine-rich receptor-like protein kinase 2	GO:0004672; GO:0005524; GO:0006468
Solyc11g065820.1	Mate efflux family protein (AHRD V1 D7KHQ9_ARALY); contains Interpro domain(s) IPR002528 Multi antimicrobial extrusion protein MatE	-0.75	1.34E-03	Mate efflux family protein 1	GO:0006855; GO:0015238; GO:0015297; GO:0016020
Solyc05g008110.2	Unknown Protein (AHRD V1)	-0.74	3.80E-04	Unknown Protein	
Solyc06g063090.2	Alanine aminotransferase (AHRD V1 **** Q6VEJ5_CAPAN); Interpro domain(s) IPR004839 Aminotransferase, class I and II	-0.74	2.17E-04	Alanine aminotransferase 2	GO:0003824; GO:0030170
Solyc06g082590.1	Ethylene responsive transcription factor 1b (AHRD *-*- C0J9I8_9ROSA); contains Interpro domain(s) IPR001471 Pathogenesis-related transcriptional factor and ERF, DNA-binding IPR017392 Pathogenesis-related	-0.74	1.30E-03	Pathogenesis-related genes transcriptional activator pti6-	GO:0003700; GO:0006355
Solyc09g075060.2	Beta-glucosidase (AHRD V1 **** D7L7Z3_ARALY); contains domain(s) IPR001360 Glycoside hydrolase, family 1	-0.74	4.98E-04	Beta-glucosidase 11	GO:0003824; GO:0005975; GO:0043169
Solyc10g007050.2	Enolase-phosphatase E-1 (AHRD V1 *-*- A8UQN2_9AQUI); Interpro domain(s) IPR010041 2,3-diketo-5-methylthio-1-phosphopentane phosphatase	-0.74	2.06E-04	Probable bifunctional methylthioribulose-1-phosphate dehydratase enolase-phosphatase e1 1	GO:0003824; GO:0008152
Solyc02g091100.2	Oxalyl-CoA decarboxylase (AHRD V1 **-- D5PWM9_COREQ); Interpro domain(s) IPR012001 Thiamine pyrophosphate enzyme, N-terminal TPP binding region	-0.73	2.16E-04	2-hydroxyacyl- lyase	GO:0030976

Solyc03g019880.2	UPF0426 protein At1g28150, chloroplastic (AHRD V1 Y1815_ARATH)	-0.73	1.58E-04	Upf0426 protein chloroplastic	
Solyc03g118970.2	Multidrug resistance protein mdtK (AHRD V1 MDTK_CITK8); contains Interpro domain(s) IPR015521 MATE family transporter related protein	-0.73	1.52E-04	Mate efflux family protein 5	GO:0006855; GO:0015238; GO:0015297; GO:0016020
Solyc04g007780.2	Major latex-like protein (AHRD V1 **-- contains Interpro domain(s) IPR000916 Bet v I allergen	-0.73	6.81E-04	Pr-10 type pathogenesis-related protein	GO:0006952; GO:0009607
Solyc04g017690.2	Early response to dehydration 15-like protein (AHRD V1 *--- Q5MEE1_PSEMZ); contains Interpro domain(s) IPR009818 Ataxin-2, C-terminal	-0.73	6.40E-04	Protein early responsive to dehydration 15	GO:0005515
Solyc08g075370.2	Unknown Protein (AHRD V1); contains Interpro IPR001464 Annexin	-0.73	6.55E-04	Unknown Protein	GO:0005509; GO:0005544
Solyc10g054670.1	Unknown Protein (AHRD V1)	-0.73	2.61E-03	Unknown Protein	
Solyc01g090900.2	Unknown Protein (AHRD V1)	-0.72	4.14E-04	Cytochrome p450	
Solyc03g095620.2	ABC-1 domain protein (AHRD V1 **-- contains Interpro domain(s) IPR004147 ABC-1	-0.72	9.40E-04	Unknown Protein	
Solyc05g050110.2	cDNA clone J013073A18 full insert sequence V1 *-*- B7EPF9_ORYSJ); contains Interpro domain(s) IPR018790 Protein of unknown function DUF2358	-0.72	2.02E-03	Unknown Protein	
Solyc11g013120.1	Protein crcB homolog (AHRD V1 *-*- contains Interpro domain(s) IPR003691 Camphor resistance CrcB protein	-0.72	2.75E-03	Protein crcB homolog	GO:0016020
Solyc12g014100.1	Homogentisate 1 2-dioxygenase (AHRD V1 ***- contains Interpro domain(s) IPR005708 Homogentisate 1,2-dioxygenase	-0.72	1.59E-03	Homogentisate -dioxygenase	GO:0004411; GO:0006559; GO:0006570; GO:0055114
Solyc02g071280.2	Ribosome maturation factor rimM (AHRD V1 A4CTC3_SYNPV); contains Interpro domain(s) IPR011961 16S rRNA processing protein RimM	-0.71	8.53E-04	16s rrna processing protein	GO:0005840; GO:0006364; GO:0043022
Solyc03g111800.2	Receptor like kinase, RLK	-0.71	7.29E-04	Leucine-rich repeat receptor-like serine threonine tyrosine-protein kinase sobir1	GO:0005515; GO:0004675
Solyc04g076820.1	Octicosapeptide/Phox/Bem1p domain-containing protein (AHRD V1 *--- contains Interpro domain(s) IPR000270 Octicosapeptide/Phox/Bem1p	-0.71	1.20E-03	Xin actin-binding repeat-containing protein	
Solyc08g007790.2	Hydroxymethylglutaryl-CoA synthase (AHRD V1 **** A9ZMZ7_HEVBR); Interpro domain(s) IPR010122 Hydroxymethylglutaryl-CoA synthase, eukaryotic IPR013746 Hydroxymethylglutaryl-coenzyme A synthase C-terminal IPR000590 Hydroxymethylglutaryl-coenzyme A	-0.71	6.22E-04	Hydroxymethylglutaryl- synthase-like	GO:0004421; GO:0008299
Solyc10g005370.2	Pyruvate, phosphate dikinase regulatory protein 2 V1 ***- PDRP2_ARATH); contains Interpro domain(s) IPR005177 Protein of unknown function DUF299	-0.71	1.45E-03	Probable phosphate dikinase regulatory chloroplastic	GO:0016772; GO:0004672; GO:0004721
Solyc01g008070.2	Alpha/beta superfamily hydrolase (AHRD V1 *--- contains Interpro domain(s) IPR010765 Protein of unknown function DUF1350	-0.7	1.44E-03	Unknown Protein	
Solyc03g116110.2	Alpha/beta hydrolase fold protein (AHRD V1 B2J569_NOSP7)	-0.7	5.73E-04	Alpha beta hydrolase family protein	
Solyc10g078600.1	Myrosinase-binding protein (Fragment) (AHRD V1 *--- contains Interpro domain(s) IPR001229 Mannose-binding lectin	-0.7	4.88E-04	Mannose-binding lectin superfamily	
Solyc01g009430.2	Os02g0448600 protein (Fragment) (AHRD V1 *---	-0.69	8.33E-04	Unknown Protein	
Solyc01g100660.2	Transcription factor (AHRD V1 ***- D7L4N5_ARALY)	-0.69	7.19E-04	Transcription factor bhlh147	
Solyc01g104720.2	Unknown Protein (AHRD V1)	-0.69	3.41E-04	Nodulin 21 -like transporter family	
Solyc01g105970.2	Magnesium-dependent phosphatase-1 family protein expressed (AHRD **** Q84R45_ORYSJ); contains Interpro domain(s) IPR010036 Magnesium-dependent phosphatase-1	-0.69	1.04E-03	Magnesium-dependent phosphatase 1	GO:0000287; GO:0016791
Solyc06g061090.2	LOC555512 protein (Fragment) (AHRD V1 *-*-	-0.69	1.14E-03	Tld protein	
Solyc11g069290.1	Glutamine amidotransferase subunit pdxT (AHRD V1 B6SNW4_MAIZE); contains Interpro domain(s) IPR002161 SNO glutamine amidotransferase	-0.69	2.62E-04	Pyridoxal biosynthesis protein pdx2	
Solyc01g086810.2	Cc-nbs-lrr, resistance protein	-0.68	2.52E-04	Disease resistance protein rpm1	GO:0006952
Solyc01g102860.2	Unknown Protein (AHRD V1)	-0.68	2.04E-03	Unknown Protein	
Solyc01g112100.2	Dimethylaniline monooxygenase 5 (AHRD V1 **** contains Interpro domain(s) IPR012143 Dimethylaniline monooxygenase, N-oxide-forming	-0.68	6.30E-04	Probable flavin-containing monooxygenase 1	GO:0004499; GO:0031227; GO:0050660; GO:0050661

Solyc02g083970.1	Chromosome 18 contig 1 DNA sequence V1 *-*- Q00SS6_OSTTA)	-0.68	5.11E-04	Unknown Protein	
Solyc03g121270.2	IAA-amino acid hydrolase (AHRD V1 **** contains Interpro domain(s) IPR017439 Peptidase M20D, mername-AA028/carboxypeptidase Ss1	-0.68	4.34E-04	Iaa-amino acid hydrolase ilr1	GO:0016787; GO:0010179
Solyc06g030490.2	Serine/threonine-protein phosphatase (AHRD V1 **** D7LDT2_ARALY); Interpro domain(s) IPR006186 Serine/threonine-specific protein phosphatase and bis(5-nucleosyl)-tetraphosphatase	-0.68	1.79E-03	Serine threonine-protein phosphatase pp1 isozyme 4	GO:0016787
Solyc07g007270.2	Unknown Protein (AHRD V1)	-0.68	1.22E-03	Unknown Protein	
Solyc07g065380.2	Zinc transporter 2 (AHRD V1 **** contains Interpro domain(s) IPR003689 Zinc/iron permease	-0.68	1.35E-03	Zinc transporter 11	GO:0016020; GO:0030001; GO:0046873
Solyc01g087030.2	Makorin RING finger protein (AHRD V1 Q7X7W8_PEA); contains Interpro domain(s) IPR000571 Zinc finger, CCCH-type	-0.67	2.21E-03	Zinc finger ccch domain-containing protein 69	GO:0005515; GO:0003682; GO:0008270
Solyc02g087060.2	Nodulin MtN21 family protein (AHRD V1 D7LPP0_ARALY)	-0.67	9.64E-04	Wat1-related protein	
Solyc03g031920.2	Yellow stripe-like protein 2.1 (Fragment) (AHRD ***- B4ZYE4_BRAJU); contains Interpro domain(s) IPR004813 Oligopeptide transporter OPT superfamily	-0.67	1.01E-03	Probable metal-nicotianamine transporter ysl7	GO:0015198; GO:0055085
Solyc04g048900.2	Calreticulin 2 calcium-binding protein (AHRD V1 A8HMC0_CHLRE); contains Interpro domain(s) IPR009169 Calreticulin	-0.67	1.63E-03	Calreticulin-3-like isoform	GO:0005509
Solyc04g080810.2	Ubiquitin-conjugating enzyme E2 W (AHRD V1 B6SI81_MAIZE); contains Interpro domain(s) IPR000608 Ubiquitin-conjugating enzyme, E2	-0.67	1.14E-03	Probable ubiquitin-conjugating enzyme e2 18	GO:0019787; GO:0043687; GO:0051246
Solyc07g043310.2	Aminotransferase (AHRD V1 ***- D2KZ08_WHEAT); contains domain(s) IPR005814 Aminotransferase class-III	-0.67	4.93E-04	Gamma aminobutyrate transaminase mitochondrial	GO:0003824; GO:0030170
Solyc09g082120.2	Glyoxalase/bleomycin resistance protein/dioxygenase (AHRD V1 ***- contains Interpro domain(s) IPR004360 Glyoxalase/bleomycin resistance protein/dioxygenase	-0.67	1.31E-03	Lactoylglutathione lyase	GO:0004462
Solyc01g102660.2	Maleylacetoacetate isomerase / glutathione S-transferase (AHRD ***- Q6MR10_BDEBA); contains Interpro domain(s) IPR005955 Maleylacetoacetate isomerase	-0.66	7.97E-04	Glutathione s-transferase zeta class	GO:0005737
Solyc01g104060.2	Aminomethyltransferase (AHRD V1 ***- B0JMM8_MICAN); contains domain(s) IPR006222 Glycine cleavage T-protein, N-terminal	-0.66	1.02E-03	Aminomethyltransferase	GO:0004047; GO:0005737; GO:0006546
Solyc02g084740.2	Cytochrome P450	-0.66	1.34E-03	3-epi-6-deoxocathasterone 23-monooxygenase	
Solyc03g112200.1	Unknown Protein (AHRD V1)	-0.66	2.42E-03	Hypothetical protein POPTR	
Solyc05g009780.2	Methionine aminopeptidase (AHRD V1 ***- B9SEJ2_RICCO); Interpro domain(s) IPR002467 Peptidase M24A, methionine aminopeptidase, subfamily 1	-0.66	1.16E-03	Methionine aminopeptidase chloroplastic	GO:0004177; GO:0006508; GO:0008235
Solyc09g082060.2	Cysteine synthase (AHRD V1 **** Q3LAG5_TOBAC); Interpro domain(s) IPR005859 Cysteine synthase A	-0.66	2.24E-03	Cysteine synthase	GO:0004124; GO:0006535
Solyc09g082730.2	Aldo/keto reductase family protein (AHRD V1 D7KWQ6_ARALY); contains Interpro domain(s) IPR001395 Aldo/keto reductase	-0.66	5.29E-04	Perakine reductase	GO:0016491; GO:0055114
Solyc09g083020.1	Myrosinase-binding protein-like protein (AHRD V1 **-* contains Interpro domain(s) IPR001229 Mannose-binding lectin	-0.66	8.25E-04	Inactive protein restricted tev movement 1	GO:0005529
Solyc09g090980.2	Major allergen Mal d 1 (AHRD ***- Q84LA7_MALDO); contains Interpro domain(s) IPR000916 Bet v I allergen	-0.66	1.33E-03	Pathogenesis-related protein sth-2	GO:0006952; GO:0009607
Solyc01g009020.2	Cysteine proteinase inhibitor (AHRD V1 **** contains Interpro domain(s) IPR000010 Proteinase inhibitor I25, cystatin	-0.65	1.32E-03	Cysteine proteinase inhibitor	GO:0004869
Solyc01g087640.2	Cinnamoyl CoA reductase-like protein (AHRD V1 A9PFK4_POPTR); contains Interpro domain(s) IPR016040 NAD(P)-binding domain	-0.65	5.22E-04	Cinnamoyl- reductase 1	GO:0003824; GO:0005488; GO:0008152
Solyc01g105420.2	Phospho-2-dehydro-3-deoxyheptonate aldolase 2 (AHRD V1 ***- contains Interpro domain(s) IPR002480 DAHP synthetase, class II	-0.65	1.37E-03	Phospho-2-dehydro-3-deoxyheptonate aldolase chloroplastic	GO:0003849; GO:0009073
Solyc02g089930.2	Homeobox protein LIM-3 (Fragment) (AHRD V1 O42286_DANRE); contains Interpro domain(s) IPR001781 Zinc finger, LIM-type	-0.65	1.39E-03	Protein da1-related 1	GO:0008270
Solyc03g123610.2	Alanine aminotransferase (AHRD V1 ***- Q6VEJ5_CAPAN); Interpro domain(s) IPR004839 Aminotransferase, class I and II	-0.65	5.49E-04	Alanine aminotransferase 2	GO:0003824; GO:0030170
Solyc05g006850.2	Thioredoxin H (AHRD V1 ***- Q4U0W0_NICAL); Interpro domain(s) IPR015467 Thioredoxin, core	-0.65	6.94E-04	Thioredoxin h2	GO:0045454

Solyc07g042190.2	Os10g0422600 protein (Fragment) (AHRD V1 *-*- contains Interpro domain(s) IPR007650 Protein of unknown function DUF581	-0.65	2.59E-03	Duf581 family protein	
Solyc12g042770.1	Chloroplast post-illumination chlorophyll fluorescence increase protein V1 **-- A1Y9I8_TOBAC)	-0.65	1.56E-03	Post-illumination chlorophyll fluorescence increase	
Solyc02g068240.2	Diacylglycerol acyltransferase family (AHRD V1 **** contains Interpro domain(s) IPR007130 Diacylglycerol acyltransferase	-0.64	2.45E-03	Diacylglycerol o-acyltransferase 2	GO:0016747
Solyc04g080010.2	UDP-glucosyltransferase (AHRD V1 **** B8QI32_9MAGN); contains domain(s) IPR002213 UDP-glucuronosyl/UDP-glucosyltransferase	-0.64	1.62E-03	Hydroquinone glucosyltransferase	GO:0008152; GO:0016758
Solyc05g052260.2	Appr-1-p processing domain protein (AHRD V1 B8FDL2_DESAA); contains Interpro domain(s) IPR002589 Appr-1-p processing	-0.64	2.27E-03	O-acetyl-adp-ribose deacetylase macrod2	
Solyc09g091840.2	Glutathione-disulfide reductase (AHRD V1 **** Q1D9Y5_MYXXD); Interpro domain(s) IPR006324 Glutathione reductase, plant	-0.64	1.14E-03	Glutathione chloroplastic	GO:0005737; GO:0016491; GO:0045454; GO:0050660
Solyc02g085310.2	Unknown Protein (AHRD V1)	-0.63	2.74E-03	Unknown Protein	
Solyc07g006140.2	Cytochrome P450	-0.63	2.32E-03	Cytochrome p450 cyp72a219	GO:0004497; GO:0005506; GO:0020037
Solyc07g042550.2	Sucrose synthase (AHRD V1 **** O82691_SOLLC); Interpro domain(s) IPR012820 Sucrose synthase, plants and cyanobacteria IPR000368 Sucrose synthase	-0.63	1.97E-03	Sucrose synthase	GO:0009058
Solyc07g045190.1	RING-H2 finger protein (AHRD V1 ***- contains Interpro domain(s) IPR018957 Zinc finger, C3HC4 RING-type	-0.63	2.22E-03	Probable e3 ubiquitin-protein ligase xerico	GO:0005515; GO:0008270
Solyc07g063010.2	Fatty acid oxidation complex subunit alpha V1 **-- FADB_ACIBC); contains Interpro domain(s) IPR006176 3-hydroxyacyl-CoA dehydrogenase, NAD binding	-0.63	1.74E-03	Probable 3-hydroxyacyl-dehydrogenase	GO:0006631; GO:0016491
Solyc01g100920.2	Nodulin-like protein (AHRD V1 ***- Q84TV1_GOSHI); Interpro domain(s) IPR000620 Protein of unknown function DUF6, transmembrane	-0.62	1.60E-03	Wat1-related protein	GO:0016020
Solyc04g076040.2	Cyclin d2 (AHRD V1 **** Q6T2Z7_SOYBN); Interpro domain(s) IPR015451 Cyclin D	-0.62	2.08E-03	D2 4-type cyclin	GO:0005634; GO:0005515; GO:0016538
Solyc07g063570.2	Cytochrome c biogenesis protein family (AHRD ***- D7MU78_ARALY); contains Interpro domain(s) IPR003834 Cytochrome c assembly protein, transmembrane region	-0.62	1.20E-03	Cytochrome c-type biogenesis ccda-like chloroplastic protein	GO:0016020; GO:0017004
Solyc01g099100.2	Long-chain-fatty-acid coa ligase (AHRD V1 ***- contains Interpro domain(s) IPR000873 AMP-dependent synthetase and ligase	-0.61	2.16E-03	Long chain acyl- synthetase peroxisomal	GO:0003824; GO:0008152
Solyc02g085640.2	Xaa-Pro aminopeptidase 1 (AHRD V1 ***- contains Interpro domain(s) IPR001131 Peptidase M24B, X-Pro dipeptidase/aminopeptidase P, conserved site IPR000994 Peptidase M24,	-0.61	1.30E-03	Probable xaa-pro aminopeptidase p	GO:0016787
Solyc03g114950.2	Lipid a export ATP-binding/permease protein msba V1 ***- Q17P67_AEDAE); contains Interpro domain(s) IPR001140 ABC transporter, transmembrane region	-0.61	1.68E-03	Abc transporter b family member 25	GO:0005524; GO:0016887
Solyc04g074750.2	Polyadenylate-binding protein 1-A (AHRD V1 **-* contains Interpro domain(s) IPR000504 RNA recognition motif, RNP-1	-0.61	1.64E-03	28 kda chloroplastic	GO:0000166
Solyc06g060790.1	3-isopropylmalate dehydratase small subunit (AHRD V1 Q9ZW85_ARATH); contains Interpro domain(s) IPR011827 3-isopropylmalate dehydratase, small subunit, subgroup	-0.61	2.50E-03	3-isopropylmalate dehydratase small subunit 3	GO:0008152
Solyc08g075860.2	Os06g0115800 protein (Fragment) (AHRD V1 *-*-	-0.61	2.74E-03	Unknown Protein	
Solyc08g077780.2	Serine/threonine protein kinase (AHRD V1 **** contains Interpro domain(s) IPR015740 Plant protein serine/threonine kinase-like	-0.61	2.47E-03	Serine threonine-protein kinase sapk3-	GO:0004674; GO:0005524; GO:0006468; GO:0006970
Solyc09g065300.2	Uncharacterized membrane protein (AHRD V1 *-*-	-0.61	2.54E-03	Unknown Protein	
Solyc04g011520.2	Serine/threonine kinase-like protein ABC1063 (AHRD V1 Q1KMP8_HORVD); contains Interpro domain(s) IPR002290 Serine/threonine protein kinase	-0.6	2.78E-03	Protein kinase chloroplastic	GO:0004672; GO:0005524; GO:0006468
Solyc10g006270.2	Autophagy-related protein 8 (AHRD V1 **-- contains Interpro domain(s) IPR004241 Light chain 3 (LC3)	-0.6	1.81E-03	Autophagy-related protein 8c	GO:0048487
Solyc10g045240.1	Beta-glucosidase D4 (AHRD V1 ***- B2ZUU0_LOTJA); Interpro domain(s) IPR001360 Glycoside hydrolase, family 1	-0.6	1.58E-03	Vicianin hydrolase-	GO:0003824; GO:0005975; GO:0043169
Solyc04g005650.1	Mitochondrial carrier family (AHRD V1 ***- contains Interpro domain(s) IPR001993 Mitochondrial substrate carrier	-0.59	2.26E-03	Peroxisomal nicotinamide adenine dinucleotide carrier	GO:0005515; GO:0016020; GO:0051724
Solyc08g029160.1	Membrane-associated progesterone receptor component 2 (AHRD **-- B6K303_SCHJY); contains Interpro domain(s) IPR001199 Cytochrome b5	-0.59	1.63E-03	Probable steroid-binding protein 3	GO:0020037

Solyc09g005620.2	Glutaredoxin (AHRD V1 *-*- D7G070_ECTSI); contains domain(s) IPR004480 Glutaredoxin-related protein	-0.59	2.34E-03	Monothiol glutaredoxin-chloroplastic	GO:0045454
Solyc09g059040.2	Alcohol dehydrogenase zinc-containing (AHRD V1 *-*- contains Interpro domain(s) IPR002085 Alcohol dehydrogenase superfamily, zinc-containing	-0.59	2.09E-03	Quinone-oxidoreductase chloroplastic	GO:0008270; GO:0016491
Solyc11g007590.1	OTU domain-containing protein 4 (AHRD V1 OTUD4_HUMAN); contains Interpro domain(s) IPR003323 Ovarian tumour, otubain	-0.59	2.41E-03	Otu domain-containing protein	
Solyc12g100200.1	Protein YdiU (AHRD V1 ***- B5N5F2_SALET); Interpro domain(s) IPR003846 Uncharacterised protein family UPF0061	-0.59	2.47E-03	Selenoprotein o-	
Solyc06g082010.2	Zinc finger CCCH domain-containing protein 66 V1 ***- C3H66_ARATH); contains Interpro domain(s) IPR002110 Ankyrin	-0.58	2.68E-03	Zinc finger ccch domain-containing protein 66	GO:0003700; GO:0008270
Solyc10g084400.1	Glutathione S-transferase (AHRD V1 ***- Q76KW1_PEA); Interpro domain(s) IPR017933 Glutathione S-transferase/chloride channel, C-terminal	-0.58	2.63E-03	Glutathione s-transferase l3	
Solyc12g096120.1	Ubiquitin-fold modifier 1 (AHRD V1 ***- contains Interpro domain(s) IPR005375 Ubiquitin-like, Ufm1	-0.58	1.62E-03	Ubiquitin-fold modifier 1	
Solyc02g063490.2	Malate dehydrogenase (AHRD V1 **** B9S7S1_RICCO); Interpro domain(s) IPR010097 Malate dehydrogenase, NAD-dependent, eukaryotes and gamma proteobacteria	-0.57	2.88E-03	Malate glyoxysomal	GO:0003824; GO:0005975
Solyc06g060260.2	Stromal ascorbate peroxidase 7	-0.57	2.41E-03	Probable l-ascorbate peroxidase chloroplastic	GO:0004601; GO:0006979; GO:0020037; GO:0055114
Solyc10g054820.1	Aquaporin (AHRD V1 ***- Q40266_MESCR); contains domain(s) IPR000425 Major intrinsic protein	-0.57	2.46E-03	X intrinsic protein	GO:0005215; GO:0006810; GO:0016020

Supplementary Table 5: Gene Set Enrichment Analysis of up-regulated genes based on the "Molecular Function" ontological domain.

GO term	Description	Number in input list	Number in BG/Ref	p-value	FDR
GO:0004674	protein serine/threonine kinase activity	39	625	1.40E-09	5.40E-07
GO:0004702	receptor signaling protein serine/threonine kinase activity	23	286	5.90E-08	7.70E-06
GO:0004672	protein kinase activity	41	772	4.00E-08	7.70E-06
GO:0005057	receptor signaling protein activity	23	292	8.30E-08	8.20E-06
GO:0004675	transmembrane receptor protein serine/threonine kinase activity	21	252	1.30E-07	1.00E-05
GO:0016773	phosphotransferase activity, alcohol group as acceptor	42	848	1.70E-07	1.10E-05
GO:0003824	catalytic activity	170	6061	3.40E-07	1.90E-05
GO:0016740	transferase activity	78	2180	5.40E-07	2.60E-05
GO:0016301	kinase activity	44	991	1.50E-06	6.40E-05
GO:0016772	transferase activity, transferring phosphorus-containing groups	47	1098	1.60E-06	6.40E-05
GO:0019199	transmembrane receptor protein kinase activity	22	333	2.50E-06	9.00E-05
GO:0060089	molecular transducer activity	27	477	3.00E-06	9.10E-05
GO:0004871	signal transducer activity	27	477	3.00E-06	9.10E-05
GO:0004888	transmembrane receptor activity	22	367	1.10E-05	3.00E-04
GO:0004872	receptor activity	23	417	2.40E-05	6.30E-04
GO:0070008	serine-type exopeptidase activity	5	31	0.00067	0.015
GO:0004185	serine-type carboxypeptidase activity	5	31	0.00067	0.015
GO:0004180	carboxypeptidase activity	5	32	0.00076	0.016
GO:0004003	ATP-dependent DNA helicase activity	5	35	0.0011	0.022
GO:0003677	DNA binding	46	1425	0.0013	0.026
GO:0004091	carboxylesterase activity	15	299	0.0015	0.027
GO:0003678	DNA helicase activity	5	38	0.0015	0.027
GO:0008094	DNA-dependent ATPase activity	5	41	0.0021	0.035
GO:0008238	exopeptidase activity	5	44	0.0027	0.045

Supplementary Table 6. Gene Set Enrichment Analysis of down-regulated genes based on the "Molecular Function" ontological domain.

GO term	Description	Number in input list	Number in BG/Ref	p-value	FDR
GO:0005215	transporter activity	45	989	4.10E-06	1.90E-03
GO:0016491	oxidoreductase activity	42	960	2.10E-05	4.90E-03
GO:0022892	substrate-specific transporter activity	35	786	7.60E-05	0.012
GO:0019825	oxygen binding	15	222	1.70E-04	0.019
GO:0019199	transmembrane receptor protein kinase activity	19	333	2.00E-04	0.019
GO:0015198	oligopeptide transporter activity	8	78	4.80E-04	0.028
GO:0015197	peptide transporter activity	8	78	4.80E-04	0.028
GO:0015103	inorganic anion transmembrane transporter activity	8	78	4.80E-04	0.028
GO:0008509	anion transmembrane transporter activity	10	127	6.90E-04	0.029
GO:0015114	phosphate transmembrane transporter activity	5	28	6.10E-04	0.029
GO:0004888	transmembrane receptor activity	19	367	6.40E-04	0.029
GO:0035251	UDP-glucosyltransferase activity	9	113	0.001	0.046
GO:0003824	catalytic activity	161	6061	0.001	0.048
GO:0022857	transmembrane transporter activity	31	786	0.001	0.048

Supplementary Table 7. KEGG analysis of the differentially expressed genes.

Mapping of enzymatic activities (Enzyme and Enzyme ID) of the DEGs (Sequence ID) in the KEGG pathways (Pathway).

Pathway	Seqs in Pathway	Enzyme	Ezyme ID	Seqs of Enzyme	Sequence ID
Starch and sucrose metabolism	17	synthase (UDP-forming)	ec:2.4.1.12	4	Solyc02g072240.2|Solyc02g072240.2.1, Solyc09g072820.2|Solyc09g072820.2.1, Solyc07g005840.2|Solyc07g005840.2.1, Solyc08g082640.2|Solyc08g082640.2.1
Starch and sucrose metabolism	17	synthase	ec:2.4.1.13	3	Solyc03g098290.2|Solyc03g098290.2.1, Solyc07g042520.2|Solyc07g042520.2.1, Solyc07g042550.2|Solyc07g042550.2.1
Starch and sucrose metabolism	17	synthase (GDP-forming)	ec:2.4.1.29	1	Solyc07g005840.2|Solyc07g005840.2.1
Starch and sucrose metabolism	17	synthase	ec:2.4.1.34	1	Solyc01g006370.2|Solyc01g006370.2.1
Starch and sucrose metabolism	17	4-alpha-galacturonosyltransferase	ec:2.4.1.43	1	Solyc05g009820.2|Solyc05g009820.2.1
Starch and sucrose metabolism	17	pectin demethoxylase	ec:3.1.1.11	3	Solyc03g123620.2|Solyc03g123620.2.1, Solyc12g008530.1|Solyc12g008530.1.1, Solyc12g099190.1|Solyc12g099190.1.1
Starch and sucrose metabolism	17	pectin depolymerase	ec:3.2.1.15	2	Solyc05g005170.2|Solyc05g005170.2.1, Solyc04g008230.2|Solyc04g008230.2.1
Starch and sucrose metabolism	17	endo-1,3-beta-D-glucosidase	ec:3.2.1.39	2	Solyc01g008620.2|Solyc01g008620.2.1, Solyc02g086700.2|Solyc02g086700.2.1
Starch and sucrose metabolism	17	endo-1,4-beta-D-glucanase	ec:3.2.1.4	1	Solyc08g083210.2|Solyc08g083210.2.1
Biosynthesis of antibiotics	16	reductase (NADPH)	ec:1.1.1.34	1	Solyc02g082260.2|Solyc02g082260.2.1
Biosynthesis of antibiotics	16	dehydrogenase	ec:1.1.1.35	1	Solyc07g063010.2|Solyc07g063010.2.1
Biosynthesis of antibiotics	16	dehydrogenase	ec:1.1.1.37	1	Solyc02g063490.2|Solyc02g063490.2.1
Biosynthesis of antibiotics	16	equilase	ec:1.11.1.6	1	Solyc12g094620.1|Solyc12g094620.1.1
Biosynthesis of antibiotics	16	dehydrogenase (NAD+)	ec:1.2.1.3	1	Solyc03g114150.2|Solyc03g114150.2.1
Biosynthesis of antibiotics	16	dehydrogenase	ec:1.2.1.41	2	Solyc12g044630.1|Solyc12g044630.1.1, Solyc06g019170.2|Solyc06g019170.2.1
Biosynthesis of antibiotics	16	dehydrogenase (NADP+, L-glutamate-forming)	ec:1.5.1.10	1	Solyc07g017610.2|Solyc07g017610.2.1
Biosynthesis of antibiotics	16	dehydrogenase (NADP+, L-lysine-forming)	ec:1.5.1.8	1	Solyc07g017610.2|Solyc07g017610.2.1
Biosynthesis of antibiotics	16	(Si)-synthase	ec:2.3.3.1	1	Solyc07g055840.2|Solyc07g055840.2.1
Biosynthesis of antibiotics	16	synthase	ec:2.3.3.10	1	Solyc08g007790.2|Solyc08g007790.2.1
Biosynthesis of antibiotics	16	synthase	ec:2.5.1.47	2	Solyc01g094790.2|Solyc01g094790.2.1, Solyc09g082060.2|Solyc09g082060.2.1
Biosynthesis of antibiotics	16	synthase	ec:2.5.1.54	1	Solyc01g105420.2|Solyc01g105420.2.1
Biosynthesis of antibiotics	16	5-kinase	ec:2.7.2.11	2	Solyc12g044630.1|Solyc12g044630.1.1, Solyc06g019170.2|Solyc06g019170.2.1
Biosynthesis of antibiotics	16	2,3-dihydro-2,3-dihydroxybenzoate synthase	ec:3.3.2.1	1	Solyc01g007920.2|Solyc01g007920.2.1
Biosynthesis of antibiotics	16	carboxylase	ec:4.1.1.39	1	Solyc02g085940.2|Solyc02g085940.2.1
Biosynthesis of antibiotics	16	synthase	ec:5.5.1.4	1	Solyc05g051850.2|Solyc05g051850.2.1
Amino sugar and nucleotide sugar metabolism	8	4-alpha-galacturonosyltransferase	ec:2.4.1.43	1	Solyc05g009820.2|Solyc05g009820.2.1

Amino sugar and nucleotide sugar metabolism	8	chitodextrinase	ec:3.2.1.14	7 Solyc02g082920.2|Solyc02g082920.2.1, Solyc02g061770.2|Solyc02g061770.2.1, Solyc07g005100.2|Solyc07g005100.2.1, Solyc05g050130.2|Solyc05g050130.2.1, Solyc10g055820.1|Solyc10g055820.1.1, Solyc10g055800.1|Solyc10g055800.1.1, Solyc10g055810.1|Solyc10g055810.1.1
Glutathione metabolism	7	peroxidase	ec:1.11.1.11	2 Solyc06g060260.2|Solyc06g060260.2.1, Solyc09g007270.2|Solyc09g007270.2.1
Phenylpropanoid biosynthesis	7	lactoperoxidase	ec:1.11.1.7	6 Solyc07g056460.2|Solyc07g056460.2.1, Solyc01g006300.2|Solyc01g006300.2.1, Solyc01g006290.2|Solyc01g006290.2.1, Solyc02g083480.2|Solyc02g083480.2.1, Solyc04g071900.2|Solyc04g071900.2.1, Solyc05g052280.2|Solyc05g052280.2.1
Glutathione metabolism	7	reductase	ec:1.8.1.7	1 Solyc09g091840.2|Solyc09g091840.2.1
Phenylpropanoid biosynthesis	7	glucosyltransferase	ec:2.4.1.128	1 Solyc01g107820.2|Solyc01g107820.2.1
Glutathione metabolism	7	transferase	ec:2.5.1.18	4 Solyc07g056460.2|Solyc07g056460.2.1, Solyc01g102660.2|Solyc01g102660.2.1, Solyc09g011590.2|Solyc09g011590.2.1, Solyc09g011580.2|Solyc09g011580.2.1
Cysteine and methionine metabolism	6	dehydrogenase	ec:1.1.1.37	1 Solyc02g063490.2|Solyc02g063490.2.1
Glyoxylate and dicarboxylate metabolism	6	dehydrogenase	ec:1.1.1.37	1 Solyc02g063490.2|Solyc02g063490.2.1
Glyoxylate and dicarboxylate metabolism	6	equilase	ec:1.11.1.6	1 Solyc12g094620.1|Solyc12g094620.1.1
Drug metabolism - cytochrome P450	6	monooxygenase	ec:1.14.13.8	1 Solyc01g112100.2|Solyc01g112100.2.1
Cysteine and methionine metabolism	6	oxidase	ec:1.14.17.4	2 Solyc07g049550.2|Solyc07g049550.2.1, Solyc07g049530.2|Solyc07g049530.2.1
Glyoxylate and dicarboxylate metabolism	6	dehydrogenase	ec:1.2.1.2	1 Solyc02g086880.2|Solyc02g086880.2.1
beta-Alanine metabolism	6	dehydrogenase (NAD+)	ec:1.2.1.3	1 Solyc03g114150.2|Solyc03g114150.2.1
Pentose and glucuronate interconversions	6	dehydrogenase (NAD+)	ec:1.2.1.3	1 Solyc03g114150.2|Solyc03g114150.2.1
Drug metabolism - cytochrome P450	6	dehydrogenase [NAD(P)+]	ec:1.2.1.5	1 Solyc06g060250.2|Solyc06g060250.2.1
beta-Alanine metabolism	6	dehydrogenase [NAD(P)+]	ec:1.2.1.5	1 Solyc06g060250.2|Solyc06g060250.2.1
beta-Alanine metabolism	6	oxidase	ec:1.4.3.21	1 Solyc08g079430.2|Solyc08g079430.2.1
Glyoxylate and dicarboxylate metabolism	6	(Si)-synthase	ec:2.3.3.1	1 Solyc07g055840.2|Solyc07g055840.2.1
Drug metabolism - cytochrome P450	6	transferase	ec:2.5.1.18	4 Solyc07g056460.2|Solyc07g056460.2.1, Solyc01g102660.2|Solyc01g102660.2.1, Solyc09g011590.2|Solyc09g011590.2.1, Solyc09g011580.2|Solyc09g011580.2.1
Cysteine and methionine metabolism	6	synthase	ec:2.5.1.47	2 Solyc01g094790.2|Solyc01g094790.2.1, Solyc09g082060.2|Solyc09g082060.2.1
Purine metabolism	6	DNA polymerase	ec:2.7.7.7	2 Solyc02g093300.2|Solyc02g093300.2.1, Solyc05g014540.2|Solyc05g014540.2.1
Pentose and glucuronate interconversions	6	pectin demethoxylase	ec:3.1.1.11	3 Solyc03g123620.2|Solyc03g123620.2.1, Solyc12g008530.1|Solyc12g008530.1.1, Solyc12g099190.1|Solyc12g099190.1.1
Cysteine and methionine metabolism	6	synthase	ec:3.1.3.77	1 Solyc10g007050.2|Solyc10g007050.2.1
Pentose and glucuronate interconversions	6	pectin depolymerase	ec:3.2.1.15	2 Solyc05g005170.2|Solyc05g005170.2.1, Solyc04g008230.2|Solyc04g008230.2.1

beta-Alanine metabolism	6	hydantoinase	ec:3.5.2.2	1	Solyc11g064920.1|Solyc11g064920.1.1
Purine metabolism	6	allantoinase	ec:3.5.2.5	1	Solyc02g078400.2|Solyc02g078400.2.1
Purine metabolism	6	adenylpyrophosphatase	ec:3.6.1.3	3	Solyc03g007690.1|Solyc03g007690.1.1, Solyc05g053600.2|Solyc05g053600.2.1, Solyc09g091660.2|Solyc09g091660.2.1
beta-Alanine metabolism	6	decarboxylase	ec:4.1.1.15	2	Solyc03g098240.2|Solyc03g098240.2.1, Solyc11g011920.1|Solyc11g011920.1.1
Glyoxylate and dicarboxylate metabolism	6	carboxylase	ec:4.1.1.39	1	Solyc02g085940.2|Solyc02g085940.2.1
Cysteine and methionine metabolism	6	1-phosphate dehydratase	ec:4.2.1.109	1	Solyc10g007050.2|Solyc10g007050.2.1
Glyoxylate and dicarboxylate metabolism	6	ligase	ec:6.2.1.8	1	Solyc03g025720.2|Solyc03g025720.2.1
Fatty acid degradation	5	dehydrogenase	ec:1.1.1.35	1	Solyc07g063010.2|Solyc07g063010.2.1
Ascorbate and aldarate metabolism	5	oxidase	ec:1.10.3.3	2	Solyc07g052240.2|Solyc07g052240.2.1, Solyc08g079090.2|Solyc08g079090.2.1
Ascorbate and aldarate metabolism	5	peroxidase	ec:1.11.1.11	2	Solyc06g060260.2|Solyc06g060260.2.1, Solyc09g007270.2|Solyc09g007270.2.1
Fatty acid degradation	5	1-monooxygenase	ec:1.14.15.3	1	Solyc01g094750.2|Solyc01g094750.2.1
Fatty acid degradation	5	dehydrogenase (NAD+)	ec:1.2.1.3	1	Solyc03g114150.2|Solyc03g114150.2.1
Ascorbate and aldarate metabolism	5	dehydrogenase (NAD+)	ec:1.2.1.3	1	Solyc03g114150.2|Solyc03g114150.2.1
Metabolism of xenobiotics by cytochrome P450	5	dehydrogenase [NAD(P)+]	ec:1.2.1.5	1	Solyc06g060250.2|Solyc06g060250.2.1
Metabolism of xenobiotics by cytochrome P450	5	transferase	ec:2.5.1.18	4	Solyc07g056460.2|Solyc07g056460.2.1, Solyc01g102660.2|Solyc01g102660.2.1, Solyc09g011590.2|Solyc09g011590.2.1, Solyc09g011580.2|Solyc09g011580.2.1
Alanine, aspartate and glutamate metabolism	5	asparaginase II	ec:3.5.1.1	1	Solyc04g078460.2|Solyc04g078460.2.1
Alanine, aspartate and glutamate metabolism	5	glutaminase I	ec:3.5.1.2	1	Solyc11g069290.1|Solyc11g069290.1.1
Alanine, aspartate and glutamate metabolism	5	decarboxylase	ec:4.1.1.15	2	Solyc03g098240.2|Solyc03g098240.2.1, Solyc11g011920.1|Solyc11g011920.1.1
Fatty acid degradation	5	isomerase	ec:5.3.3.8	1	Solyc06g053670.1|Solyc06g053670.1.1
Fatty acid degradation	5	ligase	ec:6.2.1.3	1	Solyc01g099100.2|Solyc01g099100.2.1
Alanine, aspartate and glutamate metabolism	5	synthase (glutamine-hydrolysing)	ec:6.3.5.4	1	Solyc06g007180.2|Solyc06g007180.2.1
Phenylalanine metabolism	4	dehydrogenase	ec:1.1.1.157	1	Solyc07g063010.2|Solyc07g063010.2.1
Butanoate metabolism	4	dehydrogenase	ec:1.1.1.157	1	Solyc07g063010.2|Solyc07g063010.2.1
Valine, leucine and isoleucine degradation	4	dehydrogenase	ec:1.1.1.35	1	Solyc07g063010.2|Solyc07g063010.2.1
Butanoate metabolism	4	dehydrogenase	ec:1.1.1.35	1	Solyc07g063010.2|Solyc07g063010.2.1
Tyrosine metabolism	4	1,2-dioxygenase	ec:1.13.11.5	1	Solyc12g014100.1|Solyc12g014100.1.1
Valine, leucine and isoleucine degradation	4	dehydrogenase (NAD+)	ec:1.2.1.3	1	Solyc03g114150.2|Solyc03g114150.2.1
Phenylalanine metabolism	4	dehydrogenase [NAD(P)+]	ec:1.2.1.5	1	Solyc06g060250.2|Solyc06g060250.2.1
Tyrosine metabolism	4	dehydrogenase [NAD(P)+]	ec:1.2.1.5	1	Solyc06g060250.2|Solyc06g060250.2.1
Valine, leucine and isoleucine degradation	4	dehydrogenase	ec:1.3.99.12	1	Solyc06g073560.2|Solyc06g073560.2.1
Phenylalanine metabolism	4	oxidase	ec:1.4.3.21	1	Solyc08g079430.2|Solyc08g079430.2.1
Tyrosine metabolism	4	oxidase	ec:1.4.3.21	1	Solyc08g079430.2|Solyc08g079430.2.1

Valine, leucine and isoleucine degradation	4	synthase	ec:2.3.3.10	1	Solyc08g007790.2|Solyc08g007790.2.1
Butanoate metabolism	4	synthase	ec:2.3.3.10	1	Solyc08g007790.2|Solyc08g007790.2.1
Pyrimidine metabolism	4	DNA polymerase	ec:2.7.7.7	2	Solyc02g093300.2|Solyc02g093300.2.1, Solyc05g014540.2|Solyc05g014540.2.1
Phenylalanine metabolism	4	hydrolase	ec:3.5.1.32	1	Solyc03g121270.2|Solyc03g121270.2.1
Pyrimidine metabolism	4	hydantoinase	ec:3.5.2.2	1	Solyc11g064920.1|Solyc11g064920.1.1
Pyrimidine metabolism	4	diphosphatase	ec:3.6.1.23	1	Solyc01g100030.2|Solyc01g100030.2.1
Butanoate metabolism	4	decarboxylase	ec:4.1.1.15	2	Solyc03g098240.2|Solyc03g098240.2.1, Solyc11g011920.1|Solyc11g011920.1.1
Tyrosine metabolism	4	isomerase	ec:5.2.1.2	1	Solyc01g102660.2|Solyc01g102660.2.1
Tryptophan metabolism	3	dehydrogenase	ec:1.1.1.35	1	Solyc07g063010.2|Solyc07g063010.2.1
Lysine degradation	3	dehydrogenase	ec:1.1.1.35	1	Solyc07g063010.2|Solyc07g063010.2.1
Pyruvate metabolism	3	dehydrogenase	ec:1.1.1.37	1	Solyc02g063490.2|Solyc02g063490.2.1
Pyruvate metabolism	3	dehydrogenase (oxaloacetate-decarboxylating)	ec:1.1.1.38	1	Solyc05g050120.2|Solyc05g050120.2.1
Tryptophan metabolism	3	equilase	ec:1.11.1.6	1	Solyc12g094620.1|Solyc12g094620.1.1
Arginine and proline metabolism	3	dehydrogenase (NAD+)	ec:1.2.1.3	1	Solyc03g114150.2|Solyc03g114150.2.1
Pyruvate metabolism	3	dehydrogenase (NAD+)	ec:1.2.1.3	1	Solyc03g114150.2|Solyc03g114150.2.1
Tryptophan metabolism	3	dehydrogenase (NAD+)	ec:1.2.1.3	1	Solyc03g114150.2|Solyc03g114150.2.1
Lysine degradation	3	dehydrogenase (NAD+)	ec:1.2.1.3	1	Solyc03g114150.2|Solyc03g114150.2.1
Arginine and proline metabolism	3	dehydrogenase	ec:1.2.1.41	2	Solyc12g044630.1|Solyc12g044630.1.1, Solyc06g019170.2|Solyc06g019170.2.1
Glycine, serine and threonine metabolism	3	oxidase	ec:1.4.3.21	1	Solyc08g079430.2|Solyc08g079430.2.1
Lysine degradation	3	dehydrogenase (NADP+, L-glutamate-forming)	ec:1.5.1.10	1	Solyc07g017610.2|Solyc07g017610.2.1
Lysine degradation	3	dehydrogenase (NADP+, L-lysine-forming)	ec:1.5.1.8	1	Solyc07g017610.2|Solyc07g017610.2.1
Glycine, serine and threonine metabolism	3	S-aminomethyldihydrolipoylprotein:(6S)-tetrahydrofolate aminomethyltransferase (ammonia-forming)	ec:2.1.2.10	1	Solyc01g104060.2|Solyc01g104060.2.1
Arginine and proline metabolism	3	5-kinase	ec:2.7.2.11	2	Solyc12g044630.1|Solyc12g044630.1.1, Solyc06g019170.2|Solyc06g019170.2.1
Glycine, serine and threonine metabolism	3	aldolase	ec:4.1.2.5	1	Solyc04g009960.2|Solyc04g009960.2.1
Fatty acid biosynthesis	2	reductase	ec:1.1.1.100	1	Solyc12g045030.1|Solyc12g045030.1.1
Terpenoid backbone biosynthesis	2	reductase (NADPH)	ec:1.1.1.34	1	Solyc02g082260.2|Solyc02g082260.2.1
Carbon fixation pathways in prokaryotes	2	dehydrogenase	ec:1.1.1.35	1	Solyc07g063010.2|Solyc07g063010.2.1
Caprolactam degradation	2	dehydrogenase	ec:1.1.1.35	1	Solyc07g063010.2|Solyc07g063010.2.1
Carbon fixation pathways in prokaryotes	2	dehydrogenase	ec:1.1.1.37	1	Solyc02g063490.2|Solyc02g063490.2.1
Methane metabolism	2	dehydrogenase	ec:1.1.1.37	1	Solyc02g063490.2|Solyc02g063490.2.1
Carbon fixation in photosynthetic organisms	2	dehydrogenase	ec:1.1.1.37	1	Solyc02g063490.2|Solyc02g063490.2.1
Citrate cycle (TCA cycle)	2	dehydrogenase	ec:1.1.1.37	1	Solyc02g063490.2|Solyc02g063490.2.1
Styrene degradation	2	1,2-dioxygenase	ec:1.13.11.5	1	Solyc12g014100.1|Solyc12g014100.1.1

Caprolactam degradation	2	1-monooxygenase	ec:1.14.15.3	1	Solyc01g094750.2|Solyc01g094750.2.1
Retinol metabolism	2	1-monooxygenase	ec:1.14.15.3	1	Solyc01g094750.2|Solyc01g094750.2.1
Methane metabolism	2	dehydrogenase	ec:1.2.1.2	1	Solyc02g086880.2|Solyc02g086880.2.1
Glycolysis / Gluconeogenesis	2	dehydrogenase (NAD+)	ec:1.2.1.3	1	Solyc03g114150.2|Solyc03g114150.2.1
Histidine metabolism	2	dehydrogenase (NAD+)	ec:1.2.1.3	1	Solyc03g114150.2|Solyc03g114150.2.1
Retinol metabolism	2	dehydrogenase	ec:1.2.1.36	1	Solyc03g114150.2|Solyc03g114150.2.1
Carbapenem biosynthesis	2	dehydrogenase	ec:1.2.1.41	2	Solyc12g044630.1|Solyc12g044630.1.1, Solyc06g019170.2|Solyc06g019170.2.1
Glycolysis / Gluconeogenesis	2	dehydrogenase [NAD(P)+]	ec:1.2.1.5	1	Solyc06g060250.2|Solyc06g060250.2.1
Histidine metabolism	2	dehydrogenase [NAD(P)+]	ec:1.2.1.5	1	Solyc06g060250.2|Solyc06g060250.2.1
Porphyrin and chlorophyll metabolism	2	oxidase	ec:1.3.3.5	1	Solyc05g008290.2|Solyc05g008290.2.1
Citrate cycle (TCA cycle)	2	(Si)-synthase	ec:2.3.3.1	1	Solyc07g055840.2|Solyc07g055840.2.1
Terpenoid backbone biosynthesis	2	synthase	ec:2.3.3.10	1	Solyc08g007790.2|Solyc08g007790.2.1
Galactose metabolism	2	3-alpha-galactosyltransferase	ec:2.4.1.123	1	Solyc01g079170.2|Solyc01g079170.2.1
Sulfur metabolism	2	synthase	ec:2.5.1.47	2	Solyc01g094790.2|Solyc01g094790.2.1, Solyc09g082060.2|Solyc09g082060.2.1
Inositol phosphate metabolism	2	5-kinase	ec:2.7.1.68	1	Solyc04g015470.2|Solyc04g015470.2.1
Phosphatidylinositol signaling system	2	5-kinase	ec:2.7.1.68	1	Solyc04g015470.2|Solyc04g015470.2.1
Phosphatidylinositol signaling system	2	kinase C	ec:2.7.11.13	1	Solyc02g087510.2|Solyc02g087510.2.1
Carbapenem biosynthesis	2	5-kinase	ec:2.7.2.11	2	Solyc12g044630.1|Solyc12g044630.1.1, Solyc06g019170.2|Solyc06g019170.2.1
Drug metabolism - other enzymes	2	ali-esterase	ec:3.1.1.1	1	Solyc05g013690.2|Solyc05g013690.2.1
Galactose metabolism	2	lactase (ambiguous)	ec:3.2.1.23	1	Solyc03g019890.2|Solyc03g019890.2.1
Cyanoamino acid metabolism	2	asparaginase II	ec:3.5.1.1	1	Solyc04g078460.2|Solyc04g078460.2.1
Drug metabolism - other enzymes	2	hydantoinase	ec:3.5.2.2	1	Solyc11g064920.1|Solyc11g064920.1.1
Taurine and hypotaurine metabolism	2	decarboxylase	ec:4.1.1.15	2	Solyc03g098240.2|Solyc03g098240.2.1, Solyc11g011920.1|Solyc11g011920.1.1
Carbon fixation in photosynthetic organisms	2	carboxylase	ec:4.1.1.39	1	Solyc02g085940.2|Solyc02g085940.2.1
Nitrogen metabolism	2	dehydratase	ec:4.2.1.1	1	Solyc02g067750.2|Solyc02g067750.2.1
Nitrogen metabolism	2	cyanate lyase	ec:4.2.1.104	1	Solyc09g090430.2|Solyc09g090430.2.1
Cyanoamino acid metabolism	2	synthase	ec:4.4.1.9	1	Solyc01g094790.2|Solyc01g094790.2.1
Styrene degradation	2	isomerase	ec:5.2.1.2	1	Solyc01g102660.2|Solyc01g102660.2.1
Inositol phosphate metabolism	2	synthase	ec:5.5.1.4	1	Solyc05g051850.2|Solyc05g051850.2.1
Fatty acid biosynthesis	2	ligase	ec:6.2.1.3	1	Solyc01g099100.2|Solyc01g099100.2.1
Porphyrin and chlorophyll metabolism	2	chelatase	ec:6.6.1.1	1	Solyc11g007200.1|Solyc11g007200.1.1
Biosynthesis of unsaturated fatty acids	1	reductase	ec:1.1.1.100	1	Solyc12g045030.1|Solyc12g045030.1.1
Biotin metabolism	1	reductase	ec:1.1.1.100	1	Solyc12g045030.1|Solyc12g045030.1.1
Benzoate degradation	1	dehydrogenase	ec:1.1.1.157	1	Solyc07g063010.2|Solyc07g063010.2.1
Toluene degradation	1	dehydrogenase	ec:1.1.1.35	1	Solyc07g063010.2|Solyc07g063010.2.1

Geraniol degradation	1	dehydrogenase	ec:1.1.1.35	1	Solyc07g063010.2|Solyc07g063010.2.1
Primary bile acid biosynthesis	1	dehydrogenase	ec:1.1.1.35	1	Solyc07g063010.2|Solyc07g063010.2.1
Benzoate degradation	1	dehydrogenase	ec:1.1.1.35	1	Solyc07g063010.2|Solyc07g063010.2.1
Fatty acid elongation	1	dehydrogenase	ec:1.1.1.35	1	Solyc07g063010.2|Solyc07g063010.2.1
Vitamin B6 metabolism	1	4-dehydrogenase	ec:1.1.1.65	1	Solyc09g082730.2|Solyc09g082730.2.1
alpha-Linolenic acid metabolism	1	13S-lipoxygenase	ec:1.13.11.12	1	Solyc01g006540.2|Solyc01g006540.2.1
Linoleic acid metabolism	1	13S-lipoxygenase	ec:1.13.11.12	1	Solyc01g006540.2|Solyc01g006540.2.1
Arachidonic acid metabolism	1	1-monooxygenase	ec:1.14.15.3	1	Solyc01g094750.2|Solyc01g094750.2.1
Chloroalkane and chloroalkene degradation	1	dehydrogenase (NAD+)	ec:1.2.1.3	1	Solyc03g114150.2|Solyc03g114150.2.1
Limonene and pinene degradation	1	dehydrogenase (NAD+)	ec:1.2.1.3	1	Solyc03g114150.2|Solyc03g114150.2.1
Glycerolipid metabolism	1	dehydrogenase (NAD+)	ec:1.2.1.3	1	Solyc03g114150.2|Solyc03g114150.2.1
Tropane, piperidine and pyridine alkaloid biosynthesis	1	oxidase	ec:1.4.3.21	1	Solyc08g079430.2|Solyc08g079430.2.1
Isoquinoline alkaloid biosynthesis	1	oxidase	ec:1.4.3.21	1	Solyc08g079430.2|Solyc08g079430.2.1
Lysine biosynthesis	1	dehydrogenase (NADP+, L-glutamate-forming)	ec:1.5.1.10	1	Solyc07g017610.2|Solyc07g017610.2.1
Zeatin biosynthesis	1	dehydrogenase	ec:1.5.99.12	1	Solyc04g016430.2|Solyc04g016430.2.1
One carbon pool by folate	1	S-aminomethyldihydrolipoylprotein:(6S)-tetrahydrofolate aminomethyltransferase (ammonia-forming)	ec:2.1.2.10	1	Solyc01g104060.2|Solyc01g104060.2.1
Synthesis and degradation of ketone bodies	1	synthase	ec:2.3.3.10	1	Solyc08g007790.2|Solyc08g007790.2.1
Anthocyanin biosynthesis	1	3-O-glucosyltransferase	ec:2.4.1.115	1	Solyc10g083860.1|Solyc10g083860.1.1
Glycosaminoglycan biosynthesis - heparan sulfate / heparin	1	3-beta-galactosyltransferase	ec:2.4.1.134	1	Solyc08g069060.2|Solyc08g069060.2.1
Glycosaminoglycan biosynthesis - chondroitin sulfate / dermatan sulfate	1	3-beta-galactosyltransferase	ec:2.4.1.134	1	Solyc08g069060.2|Solyc08g069060.2.1
Phenylalanine, tyrosine and tryptophan biosynthesis	1	synthase	ec:2.5.1.54	1	Solyc01g105420.2|Solyc01g105420.2.1
T cell receptor signaling pathway	1	protein-tyrosine kinase	ec:2.7.10.2	1	Solyc04g011520.2|Solyc04g011520.2.1
Indole alkaloid biosynthesis	1	esterase	ec:3.1.1.78	1	Solyc03g044790.2|Solyc03g044790.2.1
Ether lipid metabolism	1	D	ec:3.1.4.4	1	Solyc03g116620.2|Solyc03g116620.2.1
Glycerophospholipid metabolism	1	D	ec:3.1.4.4	1	Solyc03g116620.2|Solyc03g116620.2.1
Other glycan degradation	1	lactase (ambiguous)	ec:3.2.1.23	1	Solyc03g019890.2|Solyc03g019890.2.1
Glycosphingolipid biosynthesis - ganglio series	1	lactase (ambiguous)	ec:3.2.1.23	1	Solyc03g019890.2|Solyc03g019890.2.1
Sphingolipid metabolism	1	lactase (ambiguous)	ec:3.2.1.23	1	Solyc03g019890.2|Solyc03g019890.2.1
Glycosaminoglycan degradation	1	lactase (ambiguous)	ec:3.2.1.23	1	Solyc03g019890.2|Solyc03g019890.2.1
Biosynthesis of siderophore group nonribosomal peptides	1	2,3-dihydro-2,3-dihydroxybenzoate synthase	ec:3.3.2.1	1	Solyc01g007920.2|Solyc01g007920.2.1
Nicotinate and nicotinamide metabolism	1	nicotinamide deaminase	ec:3.5.1.19	1	Solyc01g106000.2|Solyc01g106000.2.1
Arginine biosynthesis	1	glutaminase I	ec:3.5.1.2	1	Solyc11g069290.1|Solyc11g069290.1.1
D-Glutamine and D-glutamate metabolism	1	glutaminase I	ec:3.5.1.2	1	Solyc11g069290.1|Solyc11g069290.1.1
Pantothenate and CoA biosynthesis	1	hydantoinase	ec:3.5.2.2	1	Solyc11g064920.1|Solyc11g064920.1.1

Valine, leucine and isoleucine biosynthesis	1	dehydratase	ec:4.2.1.33	1	Solyc06g060790.1|Solyc06g060790.1.1
Streptomycin biosynthesis	1	synthase	ec:5.5.1.4	1	Solyc05g051850.2|Solyc05g051850.2.1
Flavonoid biosynthesis	1	isomerase	ec:5.5.1.6	1	Solyc05g010320.2|Solyc05g010320.2.1

Supplementary table 8. Primer name, sequence, amplicon length, name and accession number of the genes used in this study.

Primer	Sequence (5'→3')	Amplicon length (bp)	Name	Accession Number
EF Fw Rt	CTCCATTGGGTCGTTTTGCT	101	Elongation Factor 1 alpha	X53043
EF Rv Rt	GGTCACCTTGGCACCAGTTG			
AOS Fw	GATCGGTTCGTCGGAGAAGAA	101	Allene oxide synthase	AF230371
AOS Rv	GCGCACTGTTTATTCCCCACT			
Pin I Fw	GAAACTCTCATGGCACGAAAAG	114	Proteinase inhibitor I	K03290
Pin I Rv	CACCAATAAGTTCTGGCCACAT			
Pin II Fw	CCAAAAAGGCCAAATGCTTG	116	Proteinase inhibitor II	K03291
Pin II Rv	CACAAGACGATTGTTCTGGTGG			
GCS Fw	TTGGTGAAGCCTTAACTCAGCC	102	Germacrene-C-synthase	AF03563
GCS Rv	GCAAATGGTGGTGTGCATCAT			
AP2 Fw	GCCATTTGATAACACCGCCC	101	AP2-like ethylene-responsive transcription factor	Solyc11g010710.1
AP2 Rv	TGGTTGTAGCGTTGGAAGCA			
Perox Fw	GCCAAGAGTTGTCCAAAAGCA	82	Peroxidase 3	Solyc05g046020.2.1
Perox I Rv	CAGCAGCAAGAGATGGAGCA			
PRO Fw	AGAGTCGCTTCCCCTTTTCG	87	Proline dehydrogenase	Solyc02g089620.2.1
PRO Rv	TCACGCTCTTCCTCTACCGA			
Oleosin Fw	TCGGTTTGTGGATTTGGGGT	100	Oleosin Bn-V	Solyc03g119820.1.1
Oleosin Rv	CGTAATCAACCCGGTCCGAA			
ARF9 Fw	TCCTGACAGCCTCCGATACA	103	Auxin responsive factor 9	Solyc08g082630.2.1
ARF9 Rv	GTCGGAGTCTGCTGGTTCAA			
Knotted Fw	TGGACTGTCAAAAGGTGGGT	80	Knotted-like homeobox protein	Solyc04g077210.2.1
Knotted Rv	TTGCCTCGCCTCAAACTCTT			
Al Mar Fw	TGTGGAAAAGAAGGGGAGCC	128	Aluminum-activated malate transporter	Solyc01g007080.2.1
Al Mar Rv	GCCACAGAGAACATGCAACC			
Kunitz Fw	TTGTTGGAGACGGAAGGAAGC	128	Kunitz-type proteinase inhibitor	X73986
Kunitz Rv	CGGCAAAATGGACAAAGCAC			
PPO Fw	TTTGATAGGCGGAGTTTGCG	111	Polyphenoloxydase	BI925947
PPO Rv	CCACCAGTTCAGTTATCGCCA			
WRKY40 Fw	GAAAGACAGGCAGCCACTAGGA	103	WRKY40	AK326455
WRKY40 Rv	GCCCATCCCATTTTCACGT			

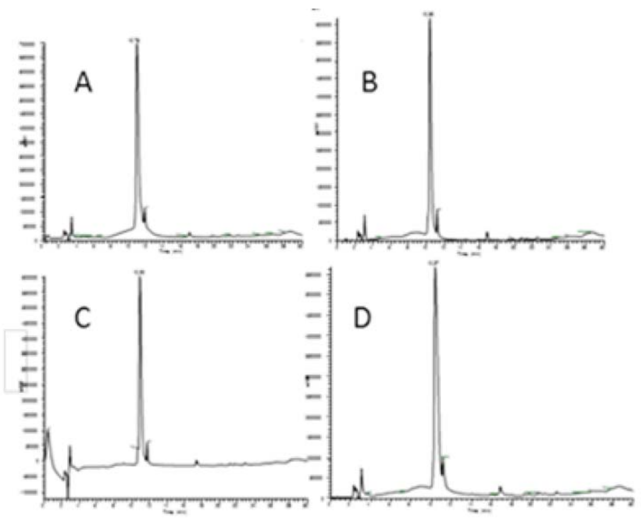

Figure S1. Stability of the systemin peptide. HPLC profiles of the systemin peptide incubated in acetic acid/acetate buffer at pH 5 at different time-points. A: reference peptide (0h); B: 6h; C: 24h; D: 48h.

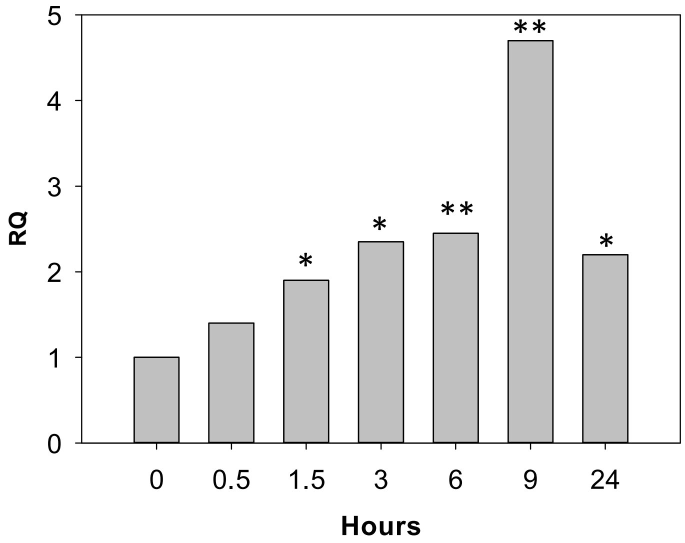

Figure S2. Time course analysis of the systemin expression in S3 plants. *Spodoptera littoralis* fourth instar larvae fed for one hour on tomato plants. Subsequently, leaves were harvested at different time points. The relative quantification (RQ) of gene expression by qRT-PCR is expressed relative to the first time of harvesting (0 hours) at end of the larva feeding. Asterisks indicate statistically significant differences compared to 0 hours (*: $p<0.05$; **: $p<0.01$; t-test).
